# Targeting intracellular cholesterol imbalance rescues sarcomere–ER contact site signaling and ER remodeling in dilated cardiomyopathy

**DOI:** 10.1038/s41392-026-02731-3

**Published:** 2026-06-17

**Authors:** Nadezda Ignatyeva, Cleophas Cheruiyot, Hafiza Nosheen Saleem, Ruheen Wali, Daria Plota, Wenjing Zhang, Sophie Schön, Soeren Brandenburg, Zhengyi Yang, Anna Steyer, Henning Urlaub, Torsten Rasmussen, Jens Mogensen, Julius Ryan D. Pronto, Yannic Döring, Michael H. Radke, Halyna Shcherbata, Stephan E. Lehnart, Andreas Janshoff, Samuel Sossalla, Torben Ruhwedel, Wiebke Moebius, Karl Toischer, Britta Brügger, Volker Haucke, Michael Gotthardt, Niels Voigt, Antje Ebert

**Affiliations:** 1 Department of Cardiology and Pneumology, Heart Research Center Goettingen, University Medical Center Goettingen, University of Goettingen, Goettingen, Germany; 2 DZHK (German Centre for Cardiovascular Research), Partner Site Lower Saxony, Goettingen, Germany; 3 Heart Center, Clinic for Cardiology and Pneumology, University Medical Center Goettingen, University of Goettingen, Goettingen, Germany; 4 EMBL Imaging Centre, European Molecular Biology Laboratory, Heidelberg, Germany; 5 Department of Clinical Chemistry, University Medical Center Goettingen, University of Goettingen, Goettingen, Germany; 6 Bioanalytical Mass Spectrometry, Max Planck Institute for Multidisciplinary Sciences, Goettingen, Germany; 7 Department of Cardiology, Aarhus University Hospital, Aarhus, Denmark; 8 Trinity College Dublin and St. Jame’s University Hospital Dublin, Dublin, Ireland; 9 Institute of Pharmacology and Toxicology, University Medical Center Goettingen, Georg-August University Goettingen, Goettingen, Germany; 10 Translational Cardiology and Functional Genomics, Max Delbrück Center for Molecular Medicine in the Helmholtz Association, Berlin, Germany; 11 German Center for Cardiovascular Research (DZHK), Partner Site Berlin, Berlin, Germany; 12 Hannover Medical School, Institute of Cell Biochemistry, Hannover, Germany; 13 Mount Desert Island Biological Laboratory, Bar Harbor, ME USA; 14 Cluster of Excellence “Multiscale Bioimaging: from Molecular Machines to Networks of Excitable Cells”, University of Goettingen, Goettingen, Germany; 15 Institute for Physical Chemistry, University of Goettingen, Goettingen, Germany; 16 Max Planck School Matter to Life, Max Planck Institute for Medical Research, Heidelberg, Germany; 17 Department of Internal Medicine II, University Medical Center Regensburg, Regensburg, Germany; 18 Department of Neurogenetics, Electron Microscopy Unit, Max Planck Institute for Multidisciplinary Sciences, Goettingen, Germany; 19 Heidelberg University Biochemistry Center (BZH), University of Heidelberg, Heidelberg, Germany; 20 Leibniz-Forschungsinstitut für Molekulare Pharmakologie (FMP), Berlin, Germany; 21 Department of Biology, Chemistry, Pharmacy, Freie Universität Berlin, Berlin, Germany; 22 Charité Universitätsmedizin Berlin, Berlin, Germany

**Keywords:** Cell biology, Cardiology, Target identification

## Abstract

Dysregulated intracellular lipid metabolism emerged as a driver of heart failure (HF), a leading cause of death worldwide that is frequently caused by dilated cardiomyopathy (DCM). Yet, how defective lipid signaling destabilizes failing cardiomyocytes (CMs) at the molecular level remains elusive. We utilized induced pluripotent stem cell-derived cardiomyocytes (iPSC-CMs), patient-derived heart tissue, and living adult CMs to elucidate lipid-dependent endoplasmic reticulum (ER) dysregulation in DCM and HF and identify new therapeutic targets. Lipidomics revealed abnormal intracellular cholesterol in iPSC-CMs carrying DCM-causing mutations in sarcomere proteins (tropomyosin; troponin T). Elevated cholesterol in CMs was found linked to abnormal ER architecture and dysfunction. STED microscopy, electron tomography, and biochemical analyses demonstrated that pathological ER remodeling and abnormal curvature are triggered by loss of ER-sarcomere/cytoskeleton contacts due to sarcomere misalignment in DCM CMs. Mechanistically, this signaling axis levers ER membrane dysfunctions and abnormal cholesterol levels via ER-sarcomere contact sites and operates in a bidirectional manner. Restoring intracellular cholesterol balance rescued ER membrane remodeling, sarcomere-ER contact site signaling, and sarcomere disarray in DCM iPSC-CMs. This ultimately improved the defective contractility of DCM iPSC-CMs, a key feature of failing CMs that contain a misaligned, dysfunctional sarcomere cytoskeleton. We validated this pathomechanism in end-stage DCM hearts and living atrial cardiomyocytes. Our findings suggest that intracellular cholesterol functions as a conserved ER membrane modulator and structural determinant across human CMs. Taken together, we present the lipidomic landscape of DCM and identify defective ER/cholesterol signaling as a disease driver whose therapeutic targeting rescues key functions in failing CMs.

## Introduction

Dilated cardiomyopathy (DCM) is the most common cause of heart failure (HF) and cardiac transplantation in young individuals. The condition is characterized by left ventricular dilatation, impaired systolic function and nonspecific histological abnormalities dominated by myocardial fibrosis. Patients may experience severe disease complications, including arrhythmia, thromboembolic events and sudden death.^1^ Despite the progress made in understanding the underlying molecular mechanisms and therapeutic delivery, DCM remains a critical cause of morbidity and mortality in patients, and there is a need for new therapeutic directions.

In DCM, impaired contractility of cardiomyocytes (CMs) ultimately leads to reduced left ventricular ejection fraction (LVEF).^2^ Key subcellular dysfunctions in DCM, such as reduced contractile force generation and altered myofilament calcium sensitivity, are recapitulated in patient-derived, human induced pluripotent stem cell-derived cardiomyocyte (iPSC-CM) models carrying inherited DCM mutations.^3–6^ Of note, recent evidence indicates lipid imbalance in end-stage DCM, suggestive of bioenergetic defects,^7–9^ yet the consequences of intracellular alterations of specific lipid species, such as cholesterol, on cardiomyocyte metabolism and contractile function in DCM and HF are not well understood.

Cholesterol contributes vitally to a variety of intracellular functions,^10^ including the membrane organization of the endoplasmic reticulum (ER), the intracellular hub of lipid synthesis, and the sarcoplasmic reticulum (SR), a specialized type of smooth ER in CMs that regulates Ca^2+^ handling and excitation-contraction coupling.^11^ However, it is not well known how lipids, especially cholesterol, regulate ER-dependent intracellular homeostasis in CMs and how this regulation contributes to pathological CM dysfunctions in DCM and HF. To investigate these questions, we employed a patient-specific and CRISPR/Cas9-edited iPSC-CM model platform of three inherited DCM mutations (troponin T, TnT-R173W and TnT-R141W; tropomyosin, TPM1-L185F).^3,5,12,13^ This allowed us to produce firstly, patient-specific iPSC-CMs (TnT-R173W, TPM1-L185F); secondly, isogenic controls by introducing DCM mutations (TnT-R141W; TPM1-L185F) into WT control iPSCs; and thirdly, DCM mutation-corrected iPSCs (TPM1-L185F) in the patient-specific background.^5,12,13^ DCM mutations in sarcomere proteins (DCM, MUT) resulted in sarcomere dysfunction, loss of sarcomere protein organization, and reduced contractility compared to WT controls. With high-content, ultrasensitive lipidomic profiling via LC‒MS/MS, we uncovered defective lipid signaling in DCM mutation–carrying iPSC-CMs. High-resolution stimulated emission depletion (STED) imaging, cryo-electron tomography (cryo-ET) mapping, and biochemical studies revealed a pathological topology and structural alteration of the endoplasmic reticulum (ER) membrane system, forming the basis for ER functional defects, including impaired processing of cholesterol in DCM iPSC-CMs. At the molecular level, structural ER destabilization was triggered by the loss of ER contact sites with the sarcomere cytoskeleton, specifically through impaired interactions mediated by the ER-transmembrane protein Cytoskeleton-Associated Protein 4 (CKAP4), as a consequence of misaligned and dysfunctional sarcomeres in DCM and HF CMs. We demonstrated that pathological ER/cholesterol signaling defects and contractile function were rescued in DCM iPSC-CMs using drugs and small molecules that lower intracellular cholesterol levels, such as pitavastatin, which is already in clinical use. Genetic rescue applying CRISPR/Cas9-mediated correction of the DCM mutation also reversed sarcomere misalignment and consequent ER/cholesterol signaling defects. Importantly, we found that this molecular pathomechanism is conserved in myocardial tissue from DCM patients with end-stage heart failure (DCM-HF).

Our results suggest that cholesterol modulation may be a promising direction to investigate in a subgroup of DCM-HF patients with inherited sarcomere protein mutations. Moreover, our findings highlight cholesterol as a critical modulator of ER membrane integrity, suggesting that its role as a structural determinant influencing intracellular lipid signaling and ER homeostasis is conserved in diverse mammalian cell types. Overall, we identified a novel molecular disease pathway triggered by sarcomere disorganization, resulting in loss of sarcomere/ER interactions, defective ER architecture, and abnormal cholesterol signaling, which ultimately exacerbates contractile defects in DCM. Our findings demonstrate that this pathway offers new therapeutic targets that may provide a basis for developing additional treatment strategies for patients suffering from DCM and HF caused by genetic mutations.

## Results

### Dysregulated lipid homeostasis is a hallmark of iPSC-derived cardiomyocytes from DCM patients harboring sarcomeric protein mutations

To assess lipid regulation of molecular patho-phenotypes in DCM patients with inherited DCM mutations at the subcellular level, we employed a platform of patient-derived iPSCs carrying heterozygous DCM mutations in cardiac troponin T (TnT-R173W) and tropomyosin 1 (TPM1-L185F), as well as healthy family controls (WT) and mutation-introduced homozygous, isogenic controls for which DCM mutations (TnT-R141W, TPM1-L185F) were introduced by CRISPR/Cas9 gene editing into WT controls (MUT)^3,5,12,13^ (Supplementary Fig. 1). Following differentiation into beating iPSC-CMs, comparable levels of cardiac markers were observed in DCM patient-specific (PAT1, PAT2), CRISPR/Cas9 mutation-introduced (MUT1, MUT2), and WT control iPSC-CMs (WT1, WT2) (Supplementary Fig. 2).

**Fig. 1:**
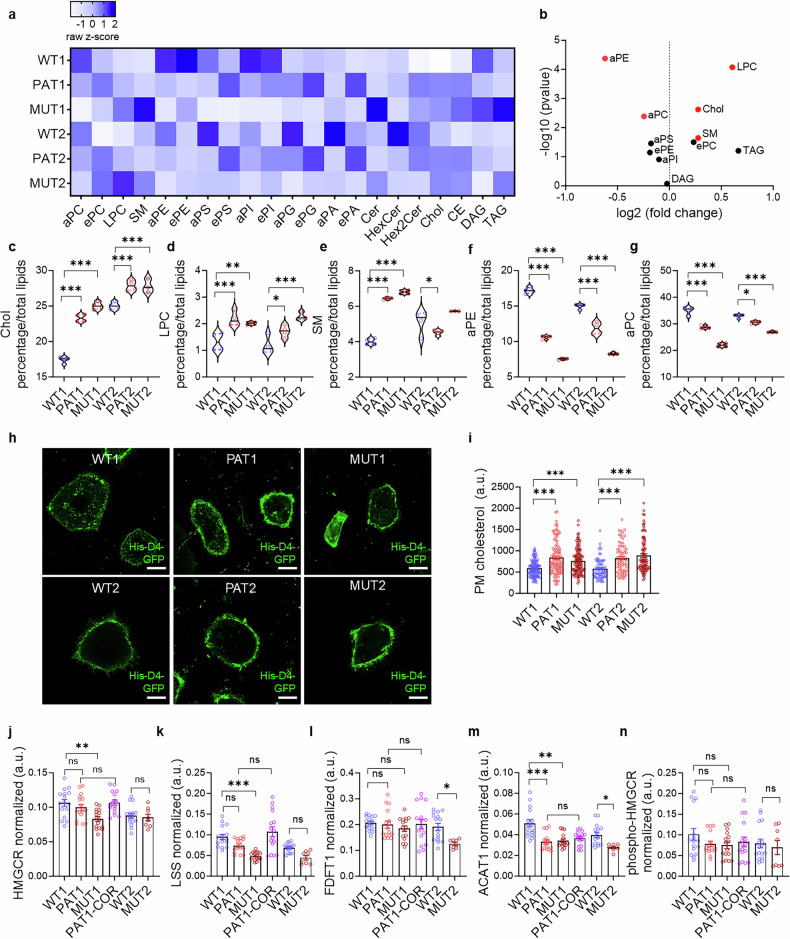
Sarcomere protein mutations cause impaired lipid signaling in induced pluripotent stem cell–derived cardiomyocytes (iPSC-CMs) from patients with dilated cardiomyopathy (DCM). **a–g** Quantitative lipidomics analyses in DCM (PAT1, MUT1, PAT2, MUT2) iPSC-CMs compared with WT (WT1, WT2) iPSC-CMs. **a** Heatmap of relative lipid amounts. **b** Volcano plot for highly abundant lipids. The top five dysregulated lipids in DCM iPSC-CMs vs WT iPSC-CMs are marked in red. **c** Cholesterol levels. ***P < 0.001 for PAT1 vs WT1, MUT1 vs WT1, PAT2 vs WT2, and MUT2 vs WT2 (one-way analysis of variance (ANOVA) with Šídák’s multiple-comparisons test). **d** Lysophosphatidylcholine levels. ***P < 0.001 for PAT1 vs WT1 and MUT2 vs WT2, **P < 0.01 for MUT1 vs WT1, *P < 0.05 for PAT2 vs WT2 (one-way analysis of variance (ANOVA) with Šídák’s multiple-comparisons test). **e** Sphingomyelin levels. ***P < 0.001 for PAT1 vs WT1 and MUT1 vs WT1, *P < 0.05 for PAT2 vs WT2 (one-way analysis of variance (ANOVA) with Šídák’s multiple-comparisons test). **f** Diacyl-phosphatidylethanolamine levels. ***P < 0.001 for PAT1 vs WT1, PAT2 vs WT2, MUT1 vs WT1, and MUT2 vs WT2 (one-way analysis of variance (ANOVA) with Šídák’s multiple-comparisons test). **g** Diacyl-phosphatidylcholine levels. ***P < 0.001 for PAT1 vs WT1, MUT1 vs WT1, and MUT2 vs WT2, *P < 0.05 for PAT2 vs WT2 (one-way analysis of variance (ANOVA) with Šídák’s multiple-comparisons test). Per group, *n* = 4 technical replicates except for MUT2 iPSC-CMs, which had *n* = 3 technical replicates. **h**, **i** Increased plasma membrane (PM) cholesterol in DCM (PAT1, MUT1, PAT2, MUT2) iPSC-CMs compared with WT (WT1, WT2) iPSC-CMs. **h** Representative images of cells stained with His-GFP-D4. Scale bar, 20 µm. **i** Quantification of (**h**). ***P < 0.001 for PAT1 vs WT1, MUT1 vs WT1, PAT2 vs WT2, MUT2 vs WT2 (Kruskal‒Wallis test, Dunn’s post-hoc comparisons). Per group, *n* = 3 experiments except for PAT2 and MUT2, which have *n* = 2 and *n* = 4 experiments, respectively; *n* = 125 cells (WT1), 103 cells (PAT1), 118 cells (MUT1), 74 cells (WT2), 71 cells (PAT2), 93 cells (MUT2). Immunoblot assessment of protein levels for key enzymes involved in cholesterol biosynthesis and turnover in DCM (PAT1, PAT2, MUT1, MUT2) iPSC-CMs compared with WT (WT1, WT2) iPSC-CMs and CRISPR/Cas9 TPM1-L185F mutation-corrected (PAT1-COR) iPSC-CMs. Quantification of HMGCR (**j**), LSS (**k**), FDFT1 (**l**), and ACAT1 (**m**). **n** Immunoblot assessment of phospho-HMGCR. Data are normalized to GAPDH. ***P < 0.001; **P < 0.01; *P < 0.05; ns not significant (Kruskal‒Wallis test, Dunn’s post-hoc comparisons). Per group, *n* = 4 experiments, *n* = 4 technical replicates except for MUT2 iPSC-CMs, which had *n* = 2 experiments. The results are reported as the mean ± SEM. Each independent experiment corresponds to a separate cardiac differentiation. A.u. indicates arbitrary units; PAT1, DCM patient-specific TPM1-L185F iPSC-CMs; PAT2, DCM patient-specific TnT-R173W iPSC-CMs; MUT1, iPSC-CMs containing CRISPR-Cas9-introduced TPM1-L185F-mutation; MUT2, iPSC-CMs containing CRISPR-Cas9-introduced TnT-R141W-mutation; PAT1-COR, CRISPR/Cas9 TPM1-L185F mutation-corrected iPSC-CMs; WT, wild-type; aPC, diacyl-phosphatidylcholine; ePC, acyl-phosphatidylcholine; LPC, lysophosphatidylcholine; SM, sphingomyelin; aPE, diacyl-phosphatidylethanolamine; ePE, acyl-phosphatidylethanolamine; aPS, diacyl-phosphatidylserine; aPI, diacyl-phosphatidylinositol; Chol, cholesterol; CE, cholesterol ester; DAG, diglyceride; TAG, triglyceride; HMGCR, 3-hydroxy-3-methylglutaryl-coenzyme A reductase; FDFT1, squalene synthase 1; LSS, lanosterol synthase; ACAT1, acyl-coenzyme A:cholesterol acyltransferase 1; phospho-HMGCR, 3-hydroxy-3-methylglutaryl-coenzyme A reductase phosphorylated at Ser872

To analyze lipid signaling in DCM patient-derived and CRISPR/Cas9-engineered iPSC-CMs, we utilized nanoscale liquid chromatography tandem mass spectrometry-based lipidome profiling (lipidomics) (Fig. 1a–g, supplementary Fig. 3, Supplementary Table 1). We discovered an imbalance of various critical lipids, including cholesterol (Fig. 1c), lysophosphatidylcholine (Fig. 1d), sphingomyelin (Fig. 1e), diacyl-phosphatidylethanolamine (Fig. 1f), and diacyl-phosphatidylcholine (Fig. 1g), in DCM patient-derived and CRISPR/Cas9-generated mutant iPSC-CMs. Strikingly, we found that the relative levels of cholesterol were significantly increased in DCM patient-specific (PAT) and mutation-introduced (MUT) iPSC-CMs compared to WT controls (Fig. 1c). Cholesterol, a key building block for cell membranes and hormones, is critically dysregulated in several cardiac diseases, such as peripheral vascular disease (PVD), coronary artery disease (CAD), arteriosclerosis and diabetes. Because of its crucial importance, minor alterations in cholesterol levels can trigger substantial changes in molecular functions in cardiomyocytes. Therefore, to validate our findings, we next analyzed the plasma membrane (PM) cholesterol content in iPSC-CMs of the DCM platform, as the PM contains the bulk of cellular cholesterol.^14^ We visualized cholesterol via a cholesterol biosensor, the GFP-labeled domain (D4) of perfringolysin O^15,16^ in patient-specific (MUT) iPSC-CMs versus wild-type controls (Fig. 1h, i). These experiments confirmed elevated cholesterol levels at the PM in DCM iPSC-CM lines (PAT1, PAT2, MUT1, and MUT2) compared to WT controls.

To understand the basis of elevated cholesterol content in DCM (MUT) iPSC-CMs, we assessed key proteins of the cholesterol biosynthetic pathway.^17^ We first quantified the protein levels of critical enzymes involved in cholesterol synthesis: HMG-CoA reductase (HMGCR), squalene synthase (FDFT1), and lanosterol synthase (LSS) (Fig. 1j–l, supplementary Fig. 4). We also assessed acyl-coenzyme A: cholesterol acyltransferase 1 (ACAT1), which creates cholesteryl esters from free cholesterol^18^ (Fig. 1m, supplementary Fig. 4). To address this comprehensively, we included in the DCM platform isogenic CRISPR/Cas9-corrected iPSC-CMs derived from iPSCs following genomic editing of the pathogenic mutation that causes DCM, TPM1-L185F (PAT1-COR) reported earlier.^5^ Patient-derived iPSC-CMs in which CRISPR/Cas9 mutation correction was performed (PAT1-COR) display molecular functions comparable to WT controls,^5^ including standard levels of pluripotency and cardiac markers, respectively (supplementary Fig. 5). We compared DCM (PAT1, MUT1, MUT2) iPSC-CMs to healthy WT controls (WT1, WT2) as well as isogenic, genome-corrected iPSC-CMs (PAT1-COR). A reduction in HMGCR (Fig. 1j) and LSS (Fig. 1k) protein levels was observed in MUT1 iPSC-CMs, whereas FDFT1 (Fig. 1l) was reduced in MUT2 iPSC-CMs compared with their respective isogenic WT controls. PAT-COR iPSC-CMs demonstrated a trend toward WT. In addition, lower ACAT1 levels (Fig. 1m) were detected in iPSC-CMs carrying DCM mutations than in WT controls. To ascertain whether the activity of HMGCR is altered, we further analyzed DCM (MUT) iPSC-CMs and controls using a phospho-HMGCR antibody, as phosphorylation is a type of posttranslational modification that activates this enzyme.^19^ The levels of phospho-HMGCR detected were similar across all groups (Fig. 1n, supplementary Fig. 4). Together, these studies addressed two potential sources of cholesterol accumulation: elevated protein levels of cholesterol-producing enzymes and enhanced enzymatic activity. Our findings indicate that in the presence of sarcomere protein mutations, increased intracellular cholesterol is unlikely to result directly from either of these mechanisms but may instead involve other regulatory factors.

### Sarcomere protein mutations cause disrupted ER network architecture in iPSC-derived cardiomyocytes from DCM patients

**Fig. 2:**
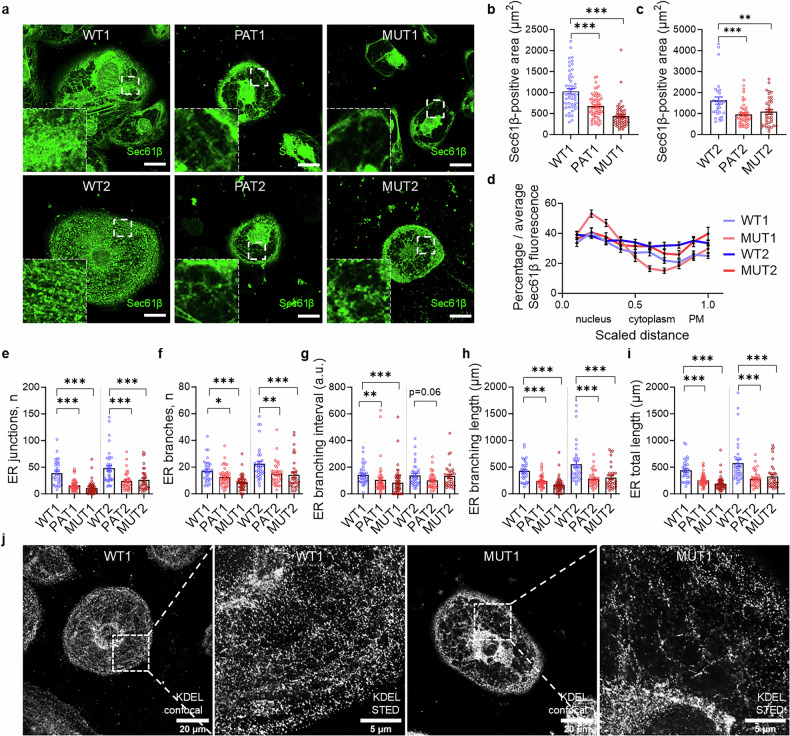
Sarcomere protein mutations cause disrupted ER network architecture in iPSC–derived cardiomyocytes from patients with DCM. **a**–**c** Decreased ER-positive intracellular area in DCM (PAT1, MUT1, PAT2, MUT2) iPSC-CMs compared with WT (WT1, WT2) iPSC-CMs. **a** Representative images of cells immunostained with an anti-Sec61β antibody. Scale bar, 20 µm. Selected regions are magnified 8.3×. **b**, **c** Quantification of (**a**). ***P < 0.001 for PAT1 vs WT1, MUT1 vs WT1, PAT2 vs WT2; **P < 0.01 for MUT2 vs WT2 (Kruskal‒Wallis test, Dunn’s post-hoc comparisons). Per group, *n* = 2 experiments except for WT1 and PAT1, which have *n* = 3 experiments; *n* = 54 cells (WT1), 68 cells (PAT1), 54 cells (MUT1), 31 cells (WT2), 49 cells (PAT2), 37 cells (MUT2). **d** Impaired ER distribution in DCM (MUT1, MUT2) iPSC-CMs compared with WT (WT1, WT2) iPSC-CMs. Per group, *n* = 2 experiments; *n* = 20 cells. **e**–**i** Quantitative analyses of ER organization in DCM (PAT1, MUT1, PAT2, MUT2) iPSC-CMs compared with WT (WT1, WT2) iPSC-CMs using images shown in (**a**). **e** Reduced number of ER junctions in the presence of DCM mutations. ***P < 0.001 for PAT1 vs WT1, MUT1 vs WT1, PAT2 vs WT2, MUT2 vs WT2 (Kruskal‒Wallis test, Dunn’s post-hoc comparisons). **f** Reduced number of ER branches in the presence of DCM mutations. ***P < 0.001 for MUT1 vs WT1, MUT2 vs WT2; **P < 0.01 for PAT2 vs WT2; *P < 0.05 for PAT1 vs WT1 (Kruskal‒Wallis test, Dunn’s post-hoc comparisons). **g** Reduced ER branching interval in the presence of DCM mutations. ***P < 0.001 for MUT1 vs WT1; **P < 0.01 for PAT1 vs WT1 (Kruskal‒Wallis test, Dunn’s post-hoc comparisons). **h** Reduced ER branching length in the presence of DCM mutations. ***P < 0.001 for PAT1 vs WT1, MUT1 vs WT1, PAT2 vs WT2, MUT2 vs WT2 (Kruskal‒Wallis test, Dunn’s post-hoc comparisons). **i** Reduced ER total length in the presence of DCM mutations. ***P < 0.001 for PAT1 vs WT1, MUT1 vs WT1, PAT2 vs WT2, MUT2 vs WT2 (Kruskal‒Wallis test, Dunn’s post-hoc comparisons). Per group, *n* = 2 experiments; *n* = 35 cells (WT1), 47 cells (PAT1), 54 cells (MUT1), 37 cells (WT2), 38 cells (PAT2), 34 cells (MUT2). **j** STED nanoscopy for KDEL immunostaining reveals patho-phenotypic ER architecture in DCM (MUT1) iPSC-CMs compared with WT1 iPSC-CMs. Representative confocal images and magnified STED images (zoom factor=5×). Per group, *n* = 1 experiment; *n* = 5 cells (WT1), 4 cells (MUT1). The results are reported as the mean ± SEM. Each independent experiment corresponds to a separate cardiac differentiation. A.u. indicates arbitrary units, PAT1 DCM patient-specific TPM1-L185F iPSC-CMs, PAT2 DCM patient-specific TnT-R173W iPSC-CMs, MUT1 iPSC-CMs containing CRISPR-Cas9-introduced TPM1-L185F-mutation, MUT2 iPSC-CMs containing CRISPR-Cas9-introduced TnT-R141W-mutation, WT wild type, PM plasma membrane

The ER is a membrane-bound system critical for lipid production and protein biosynthesis, in contrast to the SR, which is specifically adapted for Ca^2+^ handling in CMs. To reveal how imbalanced lipids contribute to pathological signaling in DCM iPSC-CMs, we assessed whether functional ER organization is dependent on altered cholesterol levels in CMs containing DCM mutations. First, we conducted quantitative image-based assessment of ER architecture using the DCM iPSC-CM platform. We analyzed the ER network-positive intracellular area as a critical parameter for ER organization, employing confocal imaging for a well-established ER marker, protein transport protein Sec61 subunit beta (Sec61β)^20^ (Fig. 2a). Strikingly, we observed a disorganized ER in iPSC-CMs with DCM mutations, with a prominent absence of the cytoplasmic ER network and only remnants of ER membranes attached to the plasma membrane. Quantification of these data measuring the Sec61β-positive intracellular area confirmed a significantly reduced ER-positive area in DCM mutation–carrying iPSC-CMs relative to wild-type controls (Fig. 2b, c). To gain further insight into the extraordinary absence of regular ER membrane organization in DCM mutation–carrying iPSC-CMs, we assessed the relative distribution of the ER network via quantification of Sec61β fluorescence in intracellular segments corresponding to the plasma membrane-adjacent region, the cytoplasm, and the nucleus-proximal area (Fig. 2d). This validated a condensation of the ER membranes in the perinuclear area and its loss in the cytoplasm in DCM mutation–carrying iPSC-CMs. We further substantiated this by analyzing intracellular network formation as reported,^21,22^ which revealed a significant reduction in ER junctions and branches (Fig. 2e, f), diminished ER branching intervals, length of branching ER (Fig. 2g, h), and total ER length (Fig. 2i) in DCM mutation–carrying iPSC-CMs. This observation was further supported by STED super-resolution microscopy (nanoscopy), which resolved the irregular distribution of ER compartments in DCM (MUT1) iPSC-CMs relative to isogenic wild-type controls (Fig. 2j).

While closely interconnected, previous studies suggest that the SR and ER represent functionally distinct compartments in CMs.^11^ Notably, contrary to the ER, we observed no significant differences in the intracellular SR area in DCM mutation–carrying iPSC-CMs versus WT control (supplementary Fig. 6a, b). Moreover, high-resolution STED imaging to detect overlap of SR and ER markers employing sarcoplasmic reticulum Ca^2+^-ATPase (SERCA2) (SR) versus KDEL (ER)^23^ or receptor accessory protein 5 (REEP5) (ER)^24^ revealed only a minor extent of overlap (supplementary Fig. 6c, d), as shown by Mander’s correlation coefficients^25^ in colocalization analysis (supplementary Fig. 6e). These observations suggest that the dysregulated architecture of the ER network but not the SR is a key pathomechanistic feature connected to disturbed cholesterol regulation in DCM cardiomyocytes. Collectively, our data demonstrate that dysregulated homeostasis of key lipids, such as cholesterol, is linked with ER architectural disorganization and defective signaling in DCM iPSC-CMs. To further assess this novel signaling pathway, we investigated whether ER structural and curvature-defining proteins contributed to the abrogation of ER architecture in DCM iPSC-CMs.

### The ER architecture of DCM (MUT) iPSC-CMs is dysfunctional due to loss of membrane curvature

**Fig. 3:**
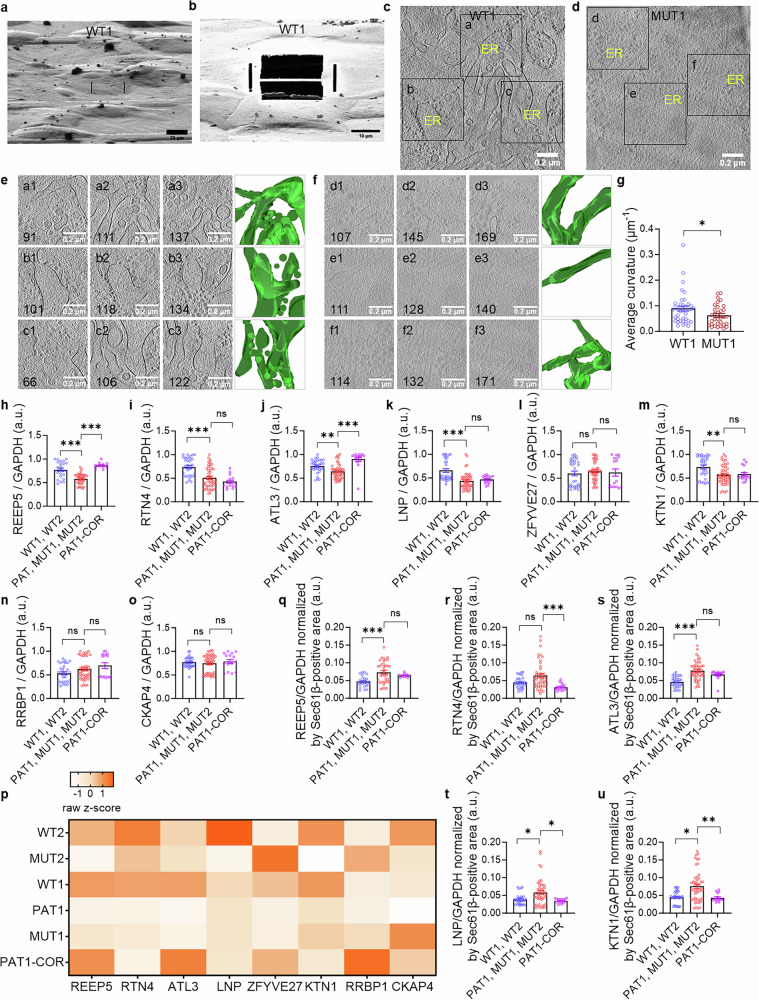
The ER architecture of DCM (MUT) iPSC-CMs is dysfunctional due to loss of membrane curvature. **a**–**e** Visualization of the ER membrane curvature in TPM1-L185F (MUT1) iPSC-CMs compared with isogenic control iPSC-CMs by cryo-electron microscopy. **a** Vitrified healthy control (WT1) iPSC-CM attached to the electron microscopy grid. Scale bar, 20 µm. **b** Cross-sectional lamella preparation using cryo-focused ion beam scanning electron microscopy (FIB-SEM). Scale bar, 10 µm. **c**, **d** Cryo-electron tomography (cryo-ET) reconstruction resolves ER membrane curvature in DCM (MUT1) iPSC-CMs compared with WT (WT1) iPSC-CMs. Representative z-slices of the three-dimensional tomograms showing the ER of WT1 iPSC-CMs (**c**) and MUT1 iPSC-CMs (**d**). Scale bar, 0.2 µm. **e**, **f** Enlarged sections (zoomed in 2.7×) of the different z-slices through the reconstructed tomograms shown in **c**, **d**, respectively, and the corresponding segmentation for the ER. The numbers in the left bottom corners of the enlarged sections indicate the serial numbers of z-slices. Scale bars, 0.2 µm. Per group, *n* = 1 experiment; *n* = 68 tilt series (WT1), *n* = 17 tilt series (MUT1). **g** Average ER membrane curvature is reduced in DCM (MUT1) iPSC-CMs compared with WT (WT1) iPSC-CMs. *P < 0.05 for MUT1 vs WT1 (Mann‒Whitney test). Per group, *n* = 1 experiment; *n* = 11 tomograms and 39 curves (WT1), *n* = 5 tomograms and 32 curves (MUT1). **h**–**p** High-content immunoblot analysis of ER structural and curvature-defining proteins in DCM (PAT1, MUT1, MUT2) iPSC-CMs compared with WT (WT1, WT2) iPSC-CMs and PAT-COR (PAT1-COR) iPSC-CMs. **h** REEP5 protein levels. ***P < 0.001 for DCM vs WT, DCM vs PAT-COR (Kruskal‒Wallis test, Dunn’s post-hoc comparisons). **i** RTN4 protein levels. ***P < 0.001 for DCM vs WT; ns, no significant differences for DCM vs PAT-COR (Kruskal‒Wallis test, Dunn’s post-hoc comparisons). **j** ATL3 protein levels. ***P < 0.001 for DCM vs PAT-COR; **P < 0.01 for DCM vs WT (Kruskal‒Wallis test, Dunn’s post-hoc comparisons). **k** LNP protein levels. ***P < 0.001 for DCM vs WT; ns, no significant differences for DCM vs PAT-COR (Kruskal‒Wallis test, Dunn’s post-hoc comparisons). **l** ZFYVE27 protein levels. ns, no significant differences for DCM vs WT and DCM vs PAT-COR (Kruskal‒Wallis test, Dunn’s post-hoc comparisons). **m** KTN1 protein levels. **P < 0.01 for DCM vs WT; ns no significant differences for DCM vs PAT-COR (Kruskal‒Wallis test, Dunn’s post-hoc comparisons). **n** RRBP1 protein levels. ns, no significant differences for DCM vs WT and DCM vs PAT-COR (Kruskal‒Wallis test, Dunn’s post-hoc comparisons). **o** CKAP4 protein levels. ns, no significant differences for DCM vs WT and DCM vs PAT-COR (Kruskal‒Wallis test, Dunn’s post-hoc comparisons). **p** Heatmap of relative protein levels. Data in **h**–**p** are normalized to GAPDH. Per group, *n* = 4 experiments, *n* = 4 technical replicates, except for REEP5 (**h**), which has *n* = 3 experiments. **q**–**u**, Protein levels detected via immunoblot (**h**–**k**, **m**) in DCM (PAT1, MUT1, MUT2) iPSC-CMs compared with WT (WT1, WT2) iPSC-CMs and PAT-COR (PAT1-COR) iPSC-CMs following normalization by the respective Sec61β-positive area analyzed by immunostaining and confocal imaging (data in Fig. 2b, c, and Fig. 6d). **q** REEP5 protein levels normalized by ER-positive area. ***P < 0.001 for DCM vs WT; ns, no significant differences for DCM vs PAT-COR (ordinary one-way ANOVA and Tukey’s multiple comparisons test). **r** RTN4 protein levels normalized by ER-positive area. ***P < 0.001 for DCM vs PAT-COR; ns, no significant differences for DCM vs WT (Kruskal‒Wallis test, Dunn’s post-hoc comparisons). **s** ATL3 protein levels normalized by ER-positive area. ***P < 0.001 for DCM vs WT; ns, no significant differences for DCM vs PAT-COR (Kruskal‒Wallis test, Dunn’s post-hoc comparisons). **t** LNP protein levels normalized by ER-positive area. *P < 0.05 for DCM vs WT and for DCM vs PAT-COR (Kruskal‒Wallis test, Dunn’s post-hoc comparisons). **u** KTN1 protein levels normalized by ER-positive area. **P < 0.01 for DCM vs PAT-COR; *P < 0.05 for DCM vs WT (Kruskal‒Wallis test, Dunn’s post-hoc comparisons). The results are reported as the mean ± SEM. A.u. indicates arbitrary units; PAT1, DCM patient-specific TPM1-L185F iPSC-CMs; MUT1, iPSC-CMs containing CRISPR-Cas9-introduced TPM1-L185F mutation; MUT2, iPSC-CMs containing CRISPR-Cas9-introduced TnT-R141W mutation; PAT1-COR, CRISPR/Cas9 TPM1-L185F mutation-corrected iPSC-CMs; WT, wild-type; ER, endoplasmic reticulum; REEP5, receptor expression-enhancing protein 5; RTN4, reticulon-4; ATL3, atlastin-3; LNP, ER junction formation protein lunapark; KTN1, kinectin-1; ZFYVE27, protrudin; RRBP1, ribosome-binding protein 1; CKAP4, cytoskeleton-associated protein 4

To assess an alteration in membrane curvature, which is key to functional ER architecture at the ultrastructural level,^26^ we employed cryo-electron tomography (cryo-ET) in DCM (MUT) iPSC-CMs versus WT controls (Fig. 3a–g, supplementary Fig. 7). Cryo-ET visualized a substantial loss of ER membrane curvature in DCM (MUT1) iPSC-CMs relative to wild-type controls (Fig. 3c–f, supplementary Fig. 7), which was quantified by analysis of reconstructed 3D tomograms (Fig. 3g). Thus, ultrastructural analysis confirmed a novel finding, severe ER remodeling, to be linked to defective cholesterol homeostasis and signaling in DCM iPSC-CMs with sarcomere protein mutations.

To assess whether disrupted ER architecture in cells harboring DCM mutations arises from altered levels of ER-shaping proteins, which regulate ER membrane curvature and the formation of ER tubules and sheets,^27,28^ we performed a high-content mini-screen employing the DCM platform (Fig. 3h–p, supplementary Fig. 8). Five proteins (REEP5, RTN4, ATL3, LNP, and KTN1) were significantly lower in the DCM groups than in the WT controls. Of note, REEP5, an ER-shaping protein recently found to be associated with heart failure,^29^ and ATL3 levels were restored after CRISPR-Cas9 genome editing correcting the DCM mutation TPM1-L185F (PAT-COR). However, DCM iPSC-CMs display a severe overall loss of ER-positive intracellular area (Fig. 2), which prompted us to normalize the levels of ER architecture-regulating proteins for the relative ER amount detected in the DCM and WT groups, respectively. After normalizing for the relative ER-positive area, no significant difference in the levels of curvature-regulating proteins was observed between the DCM (MUT) and WT groups, or the levels were increased in the DCM group (Fig. 3q–u). Therefore, decreased amounts of curvature-regulating proteins due to general signaling defects in DCM CMs may not present the basis for dysregulated cholesterol homeostasis and ER membrane architecture in CMs harboring DCM mutations. Rather, we propose that sarcomere protein DCM mutations are structurally causative for the observed pathologically disturbed ER membrane architecture and cholesterol imbalance in DCM patient-derived iPSC-CMs.

### DCM mutations impair sarcomere–ER interactions, leading to loss of ER organization in iPSC-CMs

**Fig. 4:**
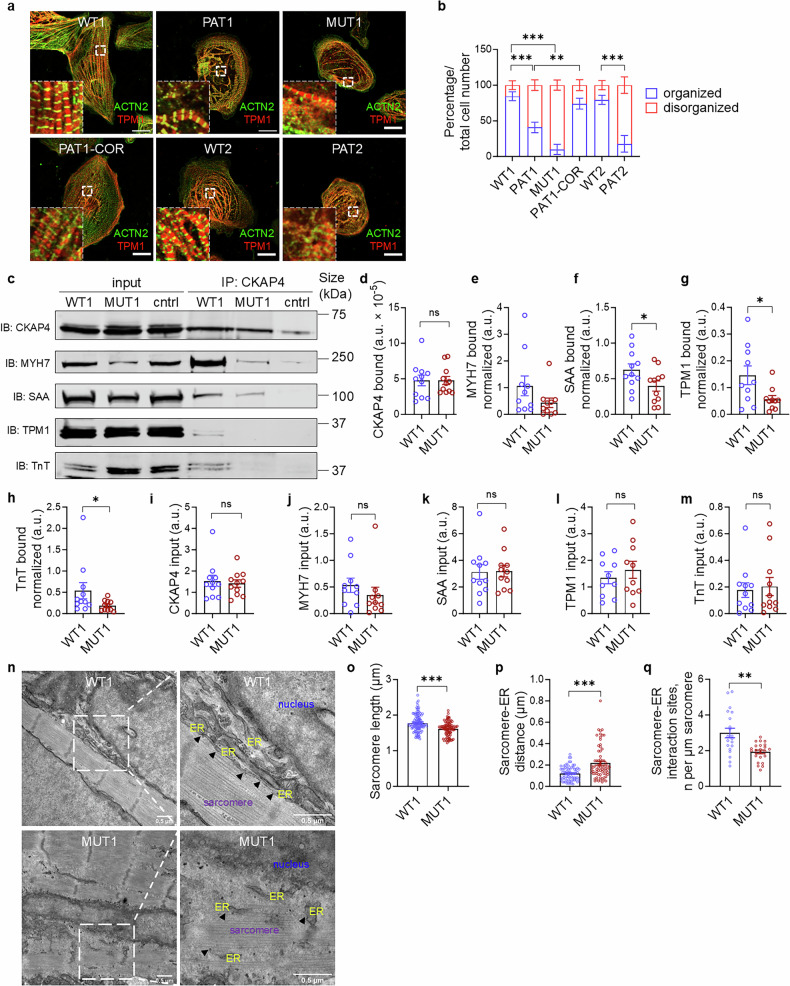
DCM mutations in sarcomere proteins disrupt sarcomere/ER connections in DCM (MUT) iPSC-CMs. **a**, **b** DCM (PAT1, MUT1, PAT2) iPSC-CMs are disorganized compared with WT (WT1, WT2) iPSC-CMs and PAT-COR (PAT1-COR) iPSC-CMs. **a** Representative images of cells immunostained with anti-TPM1 and anti-ACTN2 antibodies. Scale bar, 20 µm. Selected regions are magnified 2.3×. **b** Quantification of (**a**). Unbiased assessment was generated by three individuals independently assessing de-identified data. Data are presented as the mean ± SD. Per group, *n* = 2 experiments, except for WT1, which has *n* = 3 experiments; *n* = 63 cells (WT1), *n* = 30 cells (WT2), *n* = 27 cells (PAT1), *n* = 44 cells (MUT1), *n* = 28 cells (PAT2). ***P < 0.001 for PAT1 vs WT1, MUT1 vs WT1, and PAT2 vs WT2. **P < 0.01 for PAT1-COR vs PAT1 (ordinary one-way ANOVA followed by Tukey’s multiple comparison test). **c**–**m** Co-immunoprecipitation of sarcomere proteins with CKAP4 using cell lysates from DCM (MUT1) iPSC-CMs vs WT (WT1). Beads decorated with CKAP4-specific antibody were used. **c** Representative membrane scans for input and eluted fractions for individual proteins are shown. As a negative control, pooled lysates from MUT1 iPSC-CMs and WT1 iPSC-CMs were coupled to non-decorated beads. **d** Comparable amounts of CKAP4 were pulled down by immunoprecipitation from lysates of WT1 iPSC-CMs and DCM (MUT1) iPSC-CMs. Not significant (ns) for MUT1 vs WT1 (Mann‒Whitney test). The capacity of the sarcomere proteins MYH7 (**e**), SAA (**f**), TPM1 (**g**), and TnT (**h**) to bind CKAP4 immobilized on CKAP4 antibody-decorated beads was reduced in DCM iPSC-CMs compared to WT control iPSC-CMs. Bar graphs show the normalized signal (bound sarcomere protein divided by bound CKAP4 and normalized by input values in the respective groups). *P < 0.05 for MUT1 vs WT1 (Mann‒Whitney test). The protein levels of CKAP4 (**i**), MYH7 (**j**), SAA (**k**), TPM1 (**l**), and TnT (**m**) showed no significant difference in DCM (MUT1) iPSC-CMs compared with WT (WT1) iPSC-CMs in input fractions. Not significant (ns) for MUT1 vs WT1 (Mann‒Whitney test). Per group, *n* = 10 independent experiments (MYH7, TPM1) or *n* = 11 independent experiments (SAA, TnT). **n**–**q** Transmission electron microscopy (TEM) reveals reduced sarcomere length as well as limited contact area and interaction sites of sarcomeres and the ER in DCM (MUT1) iPSC-CMs compared with WT (WT1) iPSC-CMs. **n** Representative electron microphotographs for comparable intracellular areas in WT iPSC-CMs and DCM mutation–carrying iPSC-CMs. Selected regions are magnified 2.4×. The arrows indicate sarcomere-ER interaction sites. **o** Sarcomere length is reduced in DCM (MUT1) iPSC-CMs compared with WT (WT1) iPSC-CMs. ***P < 0.001 for MUT1 vs WT1 (Mann‒Whitney test). WT1: *n* = 7 fields of view, *n* = 105 sarcomeres; MUT1: *n* = 4 fields of view, *n* = 96 sarcomeres. **p** The distance between the Z-line of the sarcomeres and the adjoining ER domains is increased in DCM (MUT1) iPSC-CMs compared with WT (WT1) iPSC-CMs. ***P < 0.001 for MUT1 vs WT1 (Mann‒Whitney test). Per group, *n* = 1 experiment, *n* = 14 fields of view and 70 ROIs (WT1), *n* = 12 fields of view and 72 ROIs (MUT1). **q** Number of interaction sites between ER tubules and sarcomeres, normalized per 1 µm of myofibril length. **P < 0.01 for MUT1 vs WT1 (Mann‒Whitney test). Per group, *n* = 1 experiment, *n* = 14 fields of view and 20 ROIs (WT1), *n* = 12 fields of view and 24 ROIs (MUT1). The results are reported as the mean ± SEM. Each independent experiment corresponds to a separate cardiac differentiation. A.u. indicates arbitrary units, TPM1 tropomyosin-1, ACTN2 sarcomeric α-actinin, PAT1 DCM patient-specific TPM1-L185F iPSC-CMs, MUT1 iPSC-CMs containing CRISPR-Cas9-introduced TPM1-L185F mutation, PAT2 patient-specific TnT-R173W iPSC-CMs, PAT1-COR CRISPR/Cas9 TPM1-L185F mutation-corrected iPSC-CMs, WT wild-type, IP immunoprecipitation, CKAP4 cytoskeleton-associated protein 4, MYH7 myosin heavy chain 7, SAA sarcomeric α-actinin, TnT troponin T, ER endoplasmic reticulum

In cardiomyocytes, the ER is closely interconnected with sarcomeres.^30,31^ Thus, we surmised that disrupted sarcomere organization due to DCM mutations in sarcomere proteins (TPM1, TnT) may present a basis for ER dysfunctions in DCM (MUT) iPSC-CMs. Previously, we demonstrated that DCM mutations lead to the disrupted interaction of sarcomeres with other cytoskeleton elements in DCM patient-specific iPSC-CMs.^5,12^ Consistent with prior studies,^4,5,12,13^ we observed an increase in disorganized sarcomeres in the DCM (MUT) group compared to WT iPSC-CMs and DCM iPSC-CMs after CRISPR/Cas9 mutation correction (PAT-COR) (Fig. 4a, b). We hypothesized that abnormal sarcomere organization in CMs harboring DCM mutations could result in disrupted connections of sarcomeres with the ER. Therefore, we conducted co-immunoprecipitation (IP) in DCM (MUT1) iPSC-CMs relative to the isogenic wild-type control to test whether the ER-resident protein CKAP4^32,33^ interacts with sarcomere proteins (Fig. 4c–m). CKAP4-antibody-decorated beads were incubated with cell lysates from MUT iPSC-CMs or isogenic WT control iPSC-CMs and confirmed an interaction of ER-localized CKAP4 with sarcomere-resident myosin heavy chain 7 (MYH7), SAA, TPM1, and TnT. Importantly, we established a trend for a reduced interaction of CKAP4 with MYH7 and a significantly reduced interaction of CKAP4 with SAA, TPM1, and TnT in CMs with the DCM TPM1-L185F mutation (Fig. 4d–h). We also noted strong intra-experimental variation when using different batches of iPSC-CM cardiac differentiation, in line with previous reports.^5,34^ Notably, the normalized protein levels of CKAP4, MYH7, SAA, TPM1, and TnT in the input were not significantly altered in DCM mutation–carrying iPSC-CMs relative to the wild-type control (Fig. 4i–m). We next sought to confirm the loss of sarcomere/ER interaction in the presence of sarcomere protein mutations causing DCM by a different approach, ultrastructural analysis via conventional transmission electron microscopy (TEM) in DCM (MUT) iPSC-CMs and WT controls (Fig. 4n–q). Analyzing sarcomeres interacting with the ER in comparable intracellular regions, in WT controls, we found sarcomere structures to be well aligned with the ER, facilitating close interactions between the compartments. In contrast, DCM (MUT) iPSC-CMs exhibited reduced sarcomere length (Fig. 4o), in line with previous reports,^5,13^ and poor alignment of the sarcomeres with the ER. Moreover, we noted a substantial increase in the relative distance between the sarcomeres and the ER, as well as fewer sarcomere-ER interaction sites (Fig. 4p, q). These findings revealed that DCM mutations in sarcomere proteins result in defective interactions of sarcomeres with the ER in DCM (MUT) iPSC-CMs. Together, these data indicate misalignment of the sarcomere pattern and, consequently, defective sarcomere/ER dynamics to present a basis for the loss of spatial organization of ER membranes and defective cholesterol signaling in iPSC-derived CMs from DCM patients.

### Overloading cholesterol in wild-type control iPSC-CMs phenocopies the pathological ER dysfunctions observed in DCM iPSC-CMs

**Fig. 5:**
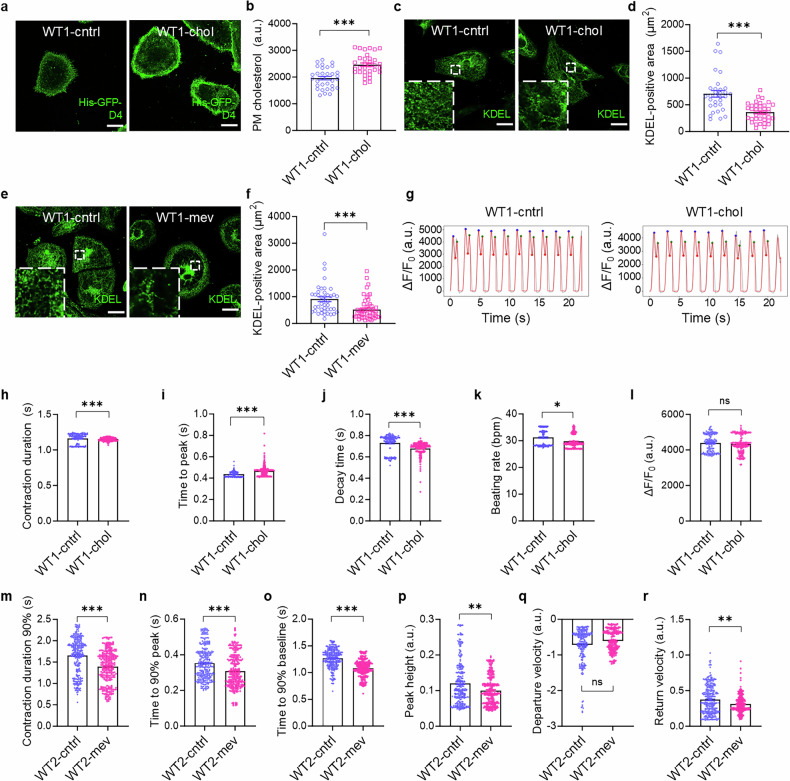
An increase in cholesterol levels phenocopies disturbed lipid signaling and disrupted ER architecture in WT control iPSC-CMs. **a**, **b** Increased plasma membrane (PM) cholesterol in healthy control iPSC-CMs treated with 2 µM water soluble cholesterol (WT1-chol) compared with control vehicle (WT1-cntrl). **a** Representative images of cells stained with His-GFP-D4. Scale bar, 20 µm. **b** Quantification of (**a**). ***P < 0.001 for WT1-chol vs WT1-cntrl (unpaired t-test). Per group, *n* = 2 experiments; *n* = 32 cells (WT1-cntrl), 33 cells (WT1-chol). **c**, **d** Decreased ER-positive area in healthy control iPSC-CMs treated with 10 µM water soluble cholesterol (WT1-chol) compared with control vehicle (WT1-cntrl). **c** Representative images of cells immunostained with an anti-KDEL antibody. Scale bar, 20 µm. Selected regions are magnified 10×. **d** Quantification of (**c**). ***P < 0.001 for WT1-chol vs WT1-cntrl (Mann‒Whitney test). Per group, *n* = 2 experiments; *n* = 32 cells (WT1-cntrl), 38 cells (WT1-chol). **e**, **f** Decreased ER-positive area in healthy control iPSC-CMs treated with 1 mM mevalonolactone (WT1-mev) compared with control vehicle (WT1-cntrl). **e** Representative imag**e**s of cells immunostained with an anti-KDEL antibody. Scale bar, 20 µm. Selected regions are magnified 10×. **f** Quantification of (**e**). ***P < 0.001 for WT1-mev vs WT1-cntrl (Mann‒Whitney test). Per group, *n* = 2 experiments; *n* = 43 cells (WT1-cntrl), 56 cells (WT1-mev). **g**–**l** Impaired contractility in healthy control iPSC-CMs treated with 10 µM water soluble cholesterol (WT1-chol) compared with control vehicle (WT1-cntrl). **g** Representative contractility traces for WT1-cntrl iPSC-CMs and WT1-chol iPSC-CMs. **h** Contraction duration was reduced after treatment. ***P < 0.001 for WT1-chol vs WT1-cntrl (Mann‒Whitney test). **i** Time to peak was increased after treatment. ***P < 0.001 for WT1-chol vs WT1-cntrl (Mann‒Whitney test). **j** Decay time was reduced after treatment. ***P < 0.001 for WT1-chol vs WT1-cntrl (Mann‒Whitney test). **k** The beating rate (beats per minute, bpm) was reduced after treatment. *P < 0.05 for WT1-chol vs WT1-cntrl (Mann‒Whitney test). **l** Amplitude was not significantly different after treatment. ns, no significant differences for WT1-chol vs WT1-cntrl (Mann‒Whitney test). Per group, *n* = 4 experiments; *n* = 161 contractility recordings (WT1-cntrl); 150 contractility recordings (WT1-chol). **m**-**r** Impaired contractility (IonOptix recording system) in healthy control iPSC-CMs treated with 1 mM mevalonolactone (WT2-mev) compared with control vehicle (WT2-cntrl). **m** Contraction duration was reduced after treatment. ***P < 0.001 for WT2-mev vs WT2-cntrl (Mann‒Whitney test). **n** Time to 90% peak was reduced after treatment. ***P < 0.001 for WT2-mev vs WT2-cntrl (Mann‒Whitney test). **o** Time to 90% baseline was reduced after treatment. ***P < 0.001 for WT2-mev vs WT2-cntrl (Mann‒Whitney test). **p** Peak height was reduced after treatment. **P < 0.01 for WT2-mev vs WT2-cntrl (Mann‒Whitney test). **q** Departure velocity remained unchanged after treatment. ns, not significant for WT2-mev vs WT2-cntrl (Mann‒Whitney test). **r** Return velocity was decreased after treatment. **P < 0.01 for WT2-mev vs WT2-cntrl (Mann‒Whitney test). Per group, *n* = 4 experiments; *n* = 306 contractility recordings (WT2-cntrl); 282 contractility recordings (WT2-mev). The results are reported as the mean ± SEM. Each independent experiment corresponds to a separate cardiac differentiation. A.u. indicates arbitrary units, WT wild-type, cntrl control, chol cholesterol, mev mevalonolactone

We next sought to further investigate the interplay of defective ER membrane architecture and altered cholesterol homeostasis occurring in DCM cells due to sarcomere protein mutations. We tested whether the pathological phenotypes observed in iPSC-CMs with DCM mutations could be introduced into wild-type control CMs by modulation of cholesterol levels. Utilizing a water-soluble form of cholesterol (chol) to increase the intracellular cholesterol levels in WT control iPSC-CMs, we monitored the resulting adverse consequences for cardiomyocyte functions. Treatment of healthy WT control iPSC-CMs (WT1) with chol resulted in elevation of the plasma membrane (PM) cholesterol levels (Fig. 5a, b), accompanied by a significant reduction in the KDEL-positive ER intracellular area in chol-treated WT iPSC-CMs compared to control vehicle (Fig. 5c, d). Importantly, this finding was reinforced at the ultrastructural level by using cryo-ET and segmentation of the ER. These experiments revealed a considerable reduction in ER tubule abundance following chol treatment (supplementary Fig. 9).

To further corroborate the pathomechanistic dysregulation of cholesterol-dependent ER dysfunction, we cultured WT control iPSC-CMs in the presence of mevalonolactone (mev), a critical intermediate in the cholesterol biosynthesis pathway,^35^ at a concentration of 1 mM.^36,37^ Like cholesterol, mev treatment also led to pathological ER remodeling in WT control iPSC-CMs (Fig. 5e, f), as observed previously in DCM mutation–carrying iPSC-CMs (Fig. 2a–c). Of note, neither chol nor mev treatment significantly altered the cellular viability of WT iPSC-CMs (supplementary Fig. 10).

Next, we explored the consequences of disrupted cholesterol/ER signaling on critical cardiomyocyte functions, such as contractility. WT iPSC-CMs treated with chol revealed a significantly decreased contraction duration, decay time, and beating rate, along with an increased time to peak (Fig. 5g–l). Moreover, IonOptix contractility analysis of WT iPSC-CMs treated with mev revealed a significant reduction in contraction duration, time to peak, and time to baseline. Altered contractile properties also included a smaller peak height (maximum amplitude of the transient) and a decreased return velocity of WT-chol-treated iPSC-CMs versus control vehicle (Fig. 5m–r). Importantly, the amplitude of WT-mev-treated CMs was reduced, reflecting a key patho-phenotypic feature of DCM patient-specific or isogenic, CRISPR/Cas mutation-introduced iPSC-CMs carrying different sarcomere protein mutations.^5,13,38^ Overall, our data demonstrated a novel pathological crosstalk of cholesterol/ER dysfunction in the presence of sarcomere DCM mutations that directly impacts a key function of cardiomyocytes, contractility.

### Pathological ER/cholesterol signaling is reversed by genetic or chemical rescue in DCM (MUT) iPSC-CMs

**Fig. 6:**
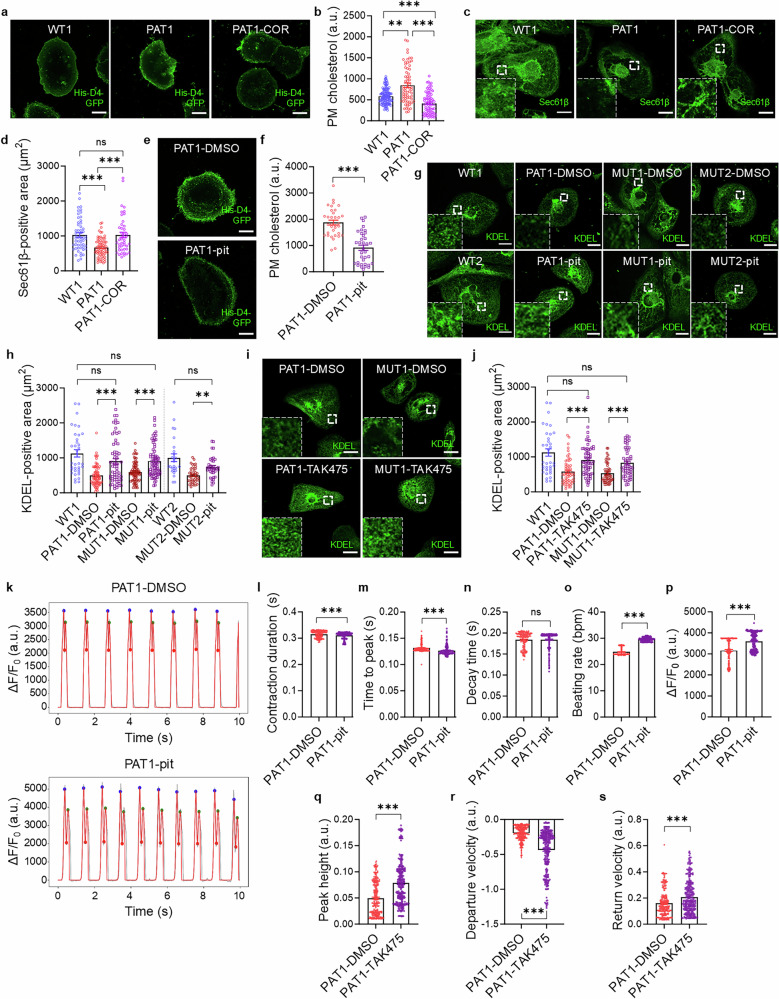
Pathological ER/cholesterol signaling is reversed by genetic or chemical rescue in DCM (MUT) iPSC-CMs. **a**, **b** Decreased plasma membrane (PM) cholesterol in patient-derived iPSC-CMs with subsequent CRISPR/Cas9-mediated correction of the mutation TPM1-L185F (PAT1-COR) relative to DCM patient-specific (PAT1) iPSC-CMs and wild-type control iPSC-CMs (WT1). **a** Representative images of cells stained with His-GFP-D4. Scale bar, 20 µm. **b** Quantification of (**a**). ***P < 0.001 for PAT1-COR vs PAT1 and for PAT1-COR vs WT1; **P < 0.01 for PAT1 vs WT1 (Kruskal‒Wallis test, Dunn’s post-hoc comparisons). Per group, *n* = 2 experiments, except for WT1 iPSC-CMs, which had *n* = 3 experiments; *n* = 125 cells (WT1), n = 67 cells (PAT1), 74 cells (PAT1-COR). Data for PAT1 and WT1 iPSC-CMs are the same as presented in Fig. 1i. **c**, **d** Increased ER-positive area in DCM patient-specific iPSC-CMs with corrected mutation TPM1-L185F (PAT1-COR) compared with DCM patient-specific (PAT1) iPSC-CMs. **c**, Representative images of cells immunostained with an anti-Sec61β antibody. Scale bar, 20 µm. Selected regions are magnified 15×. **d** Quantification of (**c**). ***P < 0.001 for PAT1-COR vs PAT1 and for PAT1 vs WT1; ns not significant for PAT1-COR vs WT1 (Kruskal‒Wallis test, Dunn’s post-hoc comparisons). Per group, *n* = 3 experiments, except for PAT1-COR iPSC-CMs, which had *n* = 2 experiments; *n* = 54 cells (WT1), *n* = 68 cells (PAT1), *n* = 44 cells (PAT1-COR). Data for WT1 and PAT1 iPSC-CMs are the same as presented in Fig. 2b. **e**, **f** Decreased plasma membrane (PM) cholesterol in DCM patient-specific (PAT1) iPSC-CMs treated with 200 nM HMG-CoA reductase inhibitor pitavastatin (PAT1-pit) compared with control vehicle (PAT1-DMSO). **e** Representative images of cells stained with His-GFP-D4. Scale bar, 20 µm. **f** Quantification of (**e**). ***P < 0.001 for PAT1-pit vs PAT1-DMSO (Mann‒Whitney test). Per group, *n* = 2 experiments; *n* = 34 cells (PAT1-DMSO), 35 cells (PAT1-pit). **g**, **h** Increased ER-positive area in DCM (PAT1, MUT1, MUT2) iPSC-CMs treated with 200 nM pitavastatin (PAT1-pit, MUT1-pit, MUT2-pit) compared with control vehicle (PAT1-DMSO, MUT1-DMSO, MUT2-DMSO) and WT iPSC-CMs (WT1, WT2). **g** Representative images of cells immunostained with an anti-KDEL antibody. Scale bar, 20 µm. Selected regions are magnified 15×. **h** Quantification of (**g**). ***P < 0.001 for PAT1-pit vs PAT1-DMSO, MUT1-pit vs MUT1-DMSO; **P < 0.01 for MUT2-pit vs MUT2-DMSO; ns not significant for PAT1-pit and MUT1-pit vs WT1, and MUT2-pit vs WT2 (Kruskal‒Wallis test, Dunn’s post-hoc comparisons). Per group, *n* = 3 experiments, except for WT1, WT2, and MUT2, which have *n* = 2 experiments; *n* = 36 cells (WT1), *n* = 76 cells (PAT1-DMSO), 63 cells (PAT1-pit), 81 cells (MUT1-DMSO), 71 cells (MUT1-pit), *n* = 37 cells (WT2), 45 cells (MUT2-DMSO), 37 cells (MUT2-pit). **i**, **j** Increased ER-positive area in DCM (PAT1, MUT1) iPSC-CMs treated with 250 nM squalene synthase inhibitor TAK475 (PAT1-TAK475, MUT1-TAK475) compared with control vehicle (PAT1-DMSO, MUT1-DMSO). **i** Representative images of cells immunostained with an anti-KDEL antibody. Scale bar, 20 µm. Selected regions are magnified 15×. **j** Quantification of (**i**). ***P < 0.001 for PAT1-TAK475 vs PAT1-DMSO and MUT1-TAK475 vs MUT1-DMSO; ns not significant for PAT1-TAK475 and MUT1-TAK475 vs WT1 (Kruskal‒Wallis test, Dunn’s post-hoc comparisons). Per group, *n* = 2 experiments; *n* = 36 cells (WT1), *n* = 59 cells (PAT1-DMSO), 59 cells (PAT1-TAK475), 52 cells (MUT1-DMSO), 54 cells (MUT1-TAK475). Data for WT1 iPSC-CMs are the same as presented in (**h**). **k**–**p** Contractile properties are rescued in DCM (PAT1) iPSC-CMs treated with 200 nM pitavastatin (PAT1-pit) compared with control vehicle (PAT1-DMSO). **k** Representative contractility traces for PAT1-DMSO iPSC-CMs and PAT1-pit iPSC-CMs. **l** Contraction duration was reduced after treatment. ***P < 0.001 for PAT1-DMSO vs PAT1-pit (Mann‒Whitney test). **m** Time to peak was reduced after treatment. ***P < 0.001 for PAT1-DMSO vs PAT1-pit (Mann‒Whitney test). **n** Decay time was not changed after treatment. ns not significant for PAT1-DMSO vs PAT1-pit (Mann‒Whitney test). **o** The beating rate was increased after treatment. ***P < 0.001 for PAT1-DMSO vs PAT1-pit (Mann‒Whitney test). **p** Amplitude was increased after treatment. ***P < 0.001 for PAT1-DMSO vs PAT1-pit (Mann‒Whitney test). Per group, *n* = 6 experiments; *n* = 158 recordings (PAT1-DMSO), 171 recordings (PAT1-pit). **q**–**s** Improved contractility (IonOptix recording system) in DCM (PAT1) iPSC-CMs treated with 250 nM squalene synthase inhibitor TAK475 (PAT1-TAK475) compared with control vehicle (PAT1-DMSO). **q** Peak height was increased after treatment. ***P < 0.001 for PAT1-TAK475 vs PAT1-DMSO (Mann‒Whitney test). **r** Departure velocity became more negative after treatment. ***P < 0.001 for PAT1-TAK475 vs PAT1-DMSO (Mann‒Whitney test). **s** Return velocity was increased after treatment. ***P < 0.001 for PAT1-TAK475 vs PAT1-DMSO (Mann‒Whitney test). Per group, *n* = 3 experiments; *n* = 282 contractility recordings (PAT1-DMSO); 285 contractility recordings (PAT1-TAK475). The results are reported as the mean ± SEM. Each independent experiment corresponds to a separate cardiac differentiation. A.u. indicates arbitrary units, PAT1 DCM patient-specific TPM1-L185F iPSC-CMs, MUT1 iPSC-CMs containing CRISPR-Cas9-introduced TPM1-L185F mutation, MUT2 iPSC-CMs containing CRISPR-Cas9-introduced TnT-R141W mutation, PAT1-COR CRISPR/Cas9 TPM1-L185F mutation-corrected iPSC-CMs, WT wild-type, pit pitavastatin

We next tested whether the newly identified patho-phenotypic ER/cholesterol dysregulation due to sarcomeric DCM mutations could be reversed by genetic or chemical targeting. First, we employed DCM mutation-corrected iPSC-CMs generated by CRISPR/Cas9 editing of the mutation TPM1-L185F (PAT-COR). We observed a restored plasma membrane cholesterol content in PAT-COR iPSC-CMs, approaching levels detected in WT controls (Fig. 6a, b). Likewise, we noted a rescue of the Sec61β-positive area, indicating recovered ER organization in PAT-COR iPSC-CMs versus isogenic patient-specific iPSC-CMs (PAT1) (Fig. 6c, d). These data are in line with inherited sarcomeric DCM mutations causing pathologically altered ER/cholesterol balance, which is recovered by genome editing to correct the DCM-specific mutation.

We next applied chemical modulation of cholesterol biosynthesis in DCM (MUT) iPSC-CMs. Statins, molecular blockers of HMGCR, are known to decrease excessive levels of cholesterol. We first confirmed that treatment with a moderate-intensity statin, pitavastatin (pit), did not alter cell viability in either WT or DCM (MUT) iPSC-CMs when administered in a dose-dependent manner (supplementary Fig. 11). However, pit treatment indeed reduced intracellular cholesterol levels in patient-specific iPSC-CMs (Fig. 6e, f). Moreover, pitavastatin rescued the dysfunctional ER morphology in DCM (MUT) iPSC-CMs carrying different DCM mutations, as shown by a significant increase in the KDEL-positive area compared with a vehicle-treated group (Fig. 6g, h), as well as improved ER architectural parameters, confirmed at the nanoscale level by super-resolution STED imaging (supplementary Fig. 12a–f). Furthermore, cryo-electron tomography confirmed that pit treatment elicited a pronounced positive effect on the ER tubule network of DCM (MUT) iPSC-CMs, compared to the control vehicle (supplementary Fig. 12g, h).

We next investigated whether pitavastatin (pit) recovered defective sarcomere-ER interactions in DCM iPSC-CMs. We evaluated the colocalization of the ER membrane-associated protein CKAP4 and sarcomeric α-actinin (ACTN2) in pit-treated DCM iPSC-CMs, given the unchanged expression levels of these proteins (Fig. 4i, k) and their reduced interaction and colocalization in the presence of sarcomeric DCM mutations versus WT controls (Fig. 4f, supplementary Fig. 13a, b). Notably, pitavastatin treatment substantially restored CKAP4-ACTN2 colocalization, indicating recovery of ER interactions with the sarcomeric cytoskeleton (supplementary Fig. 13c, d).

To further verify our findings, we utilized TAK475 (Lapaquistat),^39^ a selective inhibitor of squalene synthase. TAK475, unlike statins, acts further downstream in the synthesis of cholesterol and therefore is unlikely to affect pathways other than cholesterol-related signaling. We administered TAK475 to patient-derived (PAT) and mutation-introduced (MUT) iPSC-CMs (Fig. 6i, j). Similar to the experiments with pit-treated cells, TAK475 rescued the KDEL-positive area in PAT and MUT iPSC-CMs, confirming a link between intracellular cholesterol levels and ER architecture.

Finally, we anticipated that a disturbed ER architecture and altered lipid homeostasis due to DCM mutations in the sarcomere proteins would also negatively affect the contractile function of sarcomeres. Because sarcomeres are linked to the ER through direct contacts (Fig. 4c–h), we considered that defective sarcomere/ER interactions may exacerbate contractile defects observed in DCM iPSC-CMs. Indeed, pitavastatin enhanced contractility in DCM patient-derived iPSC-CMs relative to the control vehicle, as shown by an enhanced contraction amplitude (Fig. 6k–p). The effect of TAK-475 on DCM iPSC-CM contractile performance was also favorable, as illustrated by an elevated peak height and increased return velocity, along with a more negative departure velocity of contraction, measured using the IonOptix recording system (Fig. 6q–s). Together, these results demonstrate that defective ER/cholesterol signaling due to sarcomeric DCM mutations presents a novel molecular pathomechanism directly regulating the contractile function of iPSC-CMs, which can be rescued by chemical or drug treatments.

### Cardiac tissues from DCM patients with end-stage heart failure recapitulate lipid imbalance and defective ER organization

We next sought to explore whether this alternative pathomechanism of loss of cholesterol homeostasis and dysfunctional ER architecture is also evident in the myocardial tissues from DCM patients diagnosed with heart failure (DCM-HF). We assessed this in adult left ventricular (LV) tissues of DCM patients undergoing heart transplantation for end-stage HF. LV tissues from donor hearts that were rejected for transplantation were included as the control group^5,40^ (Supplementary Table 2). Clinical information for these patients is summarized in supplementary Table 3. One patient (DCM-HF8) received lipid-lowering therapy.

**Fig. 7:**
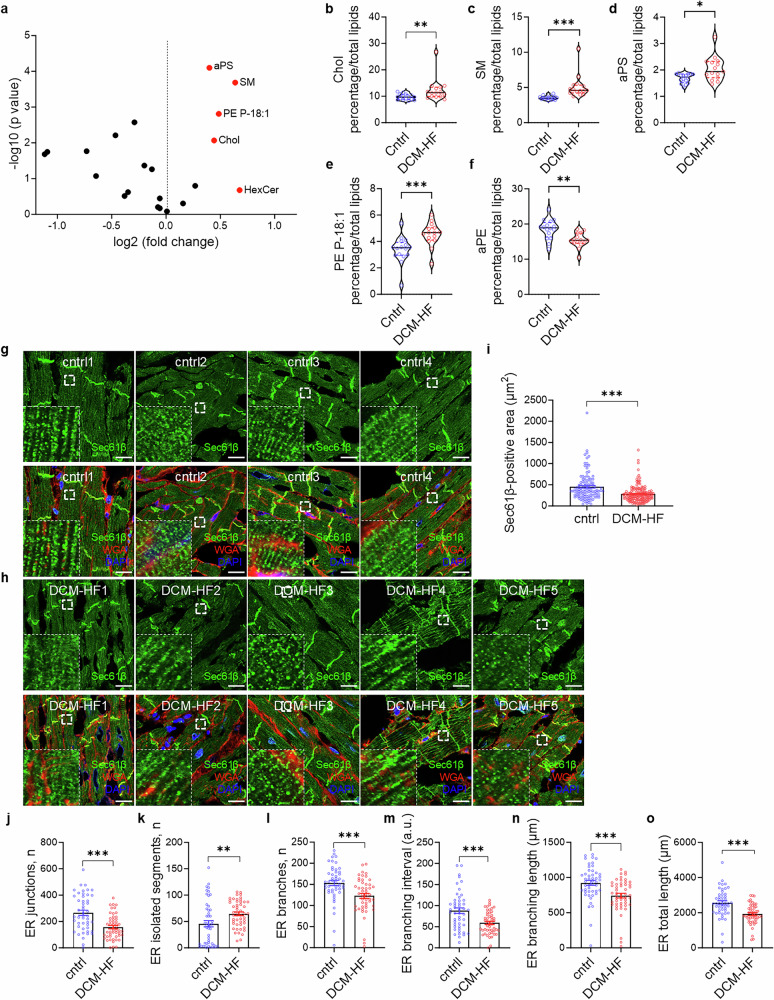
Myocardial tissue from patients with DCM and end-stage heart failure recapitulate lipid imbalance and defective ER organization. Left ventricular (LV) heart tissues from DCM patients with end-stage heart failure (DCM-HF) were analyzed compared to LV heart tissues from donor hearts (cntrl) without heart disease that failed transplantation.^40^ **a** Lipidomics results are presented as a volcano plot. The top five dysregulated lipids in DCM-HF vs control are marked in red. **b** Cholesterol levels. **P < 0.01 for DCM-HF vs cntrl (Mann‒Whitney test). **c** Sphingomyelin levels. ***P < 0.001 for DCM-HF vs cntrl (Mann‒Whitney test). **d** Diacyl-phosphatidylserine levels. *P < 0.05 for DCM-HF vs cntrl (Mann‒Whitney test). **e** Phosphatidylethanolamine with plasmalogen 18:1 moiety levels. ***P < 0.001 for DCM-HF vs cntrl (Mann‒Whitney test). **f** Diacyl-phosphatidylethanolamine levels. **P < 0.01 for DCM-HF vs cntrl (Mann‒Whitney test). Samples from 4 patients (DCM-HF2, DCM-HF3, DCM-HF7, DCM**-**HF11) and 4 control donors (cntrl) were analyzed. Per group, *n* = 4 technical replicates. **g**–**i** Analysis of ER organization in adult human heart tissue was performed by immunostaining and confocal imaging. LV tissues from DCM-HF patients display decreased ER-positive areas compared with controls. Representative images of control (**g**) vs DCM-HF (**h**) LV tissues following Sec61β-specific immunostaining (green) together with Alexa Fluor 594–conjugated wheat germ agglutinin (WGA, red) and DAPI (blue) staining. Scale bar, 20 µm. Selected regions are magnified 12×. **i** Quantification of **g** vs **h**. ***P < 0.001 for DCM-HF vs cntrl (Mann‒Whitney test). Per group, *n* = 4 donors and 110 cells (cntrl), *n* = 5 patients and 165 cells (DCM-HF). j-o, Quantitative analyses of ER organization in DCM-HF LV tissue compared with control LV tissue using images shown in (**g**, **h**). **j** Reduced number of ER junctions in DCM-HF LV tissue. ***P < 0.001 for DCM-HF vs cntrl (Mann‒Whitney test). **k** Increased number of ER-isolated segments in DCM-HF LV tissue. **P < 0.01 for DCM-HF vs cntrl (Mann‒Whitney test). **l** Reduced number of ER branches in DCM-HF LV tissue. ***P < 0.001 for DCM-HF vs cntrl (Mann‒Whitney test). **m** Reduced ER branching interval in DCM-HF LV tissue. ***P < 0.001 for DCM-HF vs cntrl (Mann‒Whitney test). **n** Reduced ER branching length in DCM-HF LV tissue. ***P < 0.001 for DCM-HF vs cntrl (Mann‒Whitney test). **o** Reduced ER total length in DCM-HF LV tissue. ***P < 0.001 for DCM-HF vs cntrl (Mann‒Whitney test). Per group, *n* = 4 donors and 48 images (cntrl), *n* = 5 patients and 50 images (DCM-HF). The results are reported as the mean ± SEM. A.u. indicates arbitrary units, cntrl control, DCM-HF heart failure, Chol cholesterol, SM sphingomyelin, aPS diacyl-phosphatidylserine, PE (P-18:1) phosphatidylethanolamine with plasmalogen 18:1 moiety, aPE diacyl-phosphatidylethanolamine

We first subjected LV samples from four DCM-HF patients, none of whom were diagnosed with hyperlipidemia or diabetes, to lipidomic analyses. Similar to findings in DCM iPSC-CMs, our data revealed a substantial lipid imbalance in DCM hearts (Fig. 7a–f), with higher percentages of cholesterol (Fig. 7b), sphingomyelin (Fig. 7c), diacyl-phosphatidylserine (Fig. 7d), and phosphatidylethanolamine plasmalogen (Fig. 7e), while diacyl-phosphatidylethanolamine levels were reduced (Fig. 7f). To further investigate a potential alteration of ER organization in the LV tissues from DCM-HF patients (*n* = 5) compared to donors (*n* = 4), we established antibody-based detection of ER-localized Sec61β together with WGA.^41^ Donor tissues displayed a well-organized and aligned ER pattern (Fig. 7g), which was substantially disrupted in the tissues of DCM-HF patients (Fig. 7h). The Sec61β-positive ER area was significantly reduced in LV tissues from DCM-HF patients versus donors (Fig. 7i). We also quantified ER organization based on the Sec61β-positive area in confocal imaging (Fig. 7j–o) and observed a significant decrease in the number of ER junctions and branches in DCM-HF patient tissues, as well as more isolated ER segments. Moreover, the ER branching interval, branching length and total length were reduced in the CMs of DCM-HF patients. Negative controls for human heart tissue staining are presented in supplementary Fig. 14.

### DCM heart tissues exhibit dysregulation of ER structural and curvature-defining proteins

**Fig. 8:**
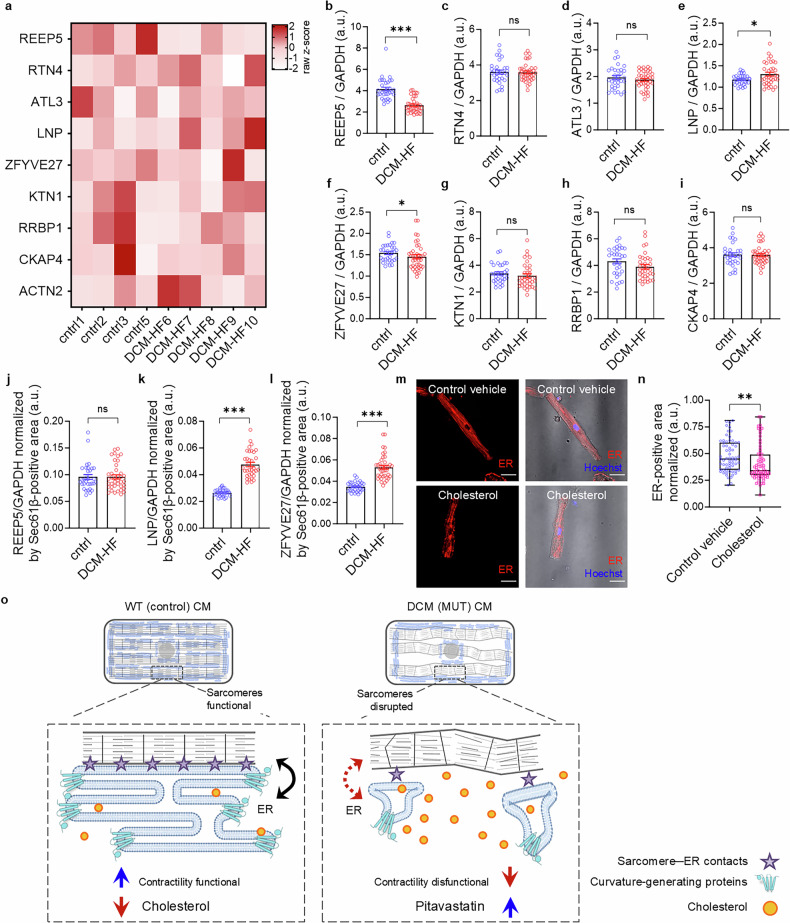
Analysis of structural and curvature-defining ER proteins in DCM heart tissue and analysis of the ER following cholesterol-overload in adult cardiomyocytes. **a**–**i** High-content immunoblot analysis of ER structural and curvature-defining proteins in left ventricular (LV) heart tissues from DCM patients with end-stage heart failure (DCM-HF) compared to LV heart tissues from donor hearts (cntrl) without heart disease that failed transplantation.^40^ Patient DCM-HF8 received lipid-lowering therapy (simvastatin). **a** Heatmap of relative protein levels. Data are normalized to GAPDH and to respective protein values in the cntrl1 group. **b** REEP5 protein levels. ***P < 0.001 for DCM-HF vs cntrl (Mann‒Whitney test). **c** RTN4 protein levels. ns, not significant for DCM-HF vs cntrl (unpaired t-test). **d** ATL3 protein levels. ns, not significant for DCM-HF vs cntrl (Mann‒Whitney test). **e** LNP prot**e**in levels. *P < 0.05 for DCM-HF vs cntrl (unpaired t-test). **f** ZFYVE27 protein levels. *P < 0.05 for DCM-HF vs cntrl (Mann‒Whitney test). **g** KTN1 protein levels. ns not si**g**nificant for DCM-HF vs cntrl (Mann‒Whitney test). **h** RRBP1 protein levels. ns, not significant for DCM-HF vs cntrl (Mann‒Whitney test). **i** CKAP4 protein levels. ns, not s**i**gnificant for DCM-HF vs cntrl (unpaired t-test). Data in **b**–**i** are normalized to GAPDH. Per group, *n* = 2 experiments, *n* = 4 technical replicates, except for DCM-HF9, which has *n* = 1 experiment for proteins RTN4, LNP, KTN1, RRBP1, ACTN2. **j**–**l** Protein levels detected via immunoblot in human LV heart tissue from patients with DCM-HF compared with cntrl following normalization by the respective Sec61β-positive area analyzed by immunostaining and confocal imaging. **j** REEP5 protein levels normalized to the ER-positive area. ns no significant differences for DCM-HF vs cntrl (Mann‒Whitney test). k Lunapark protein levels normalized to the ER-positive area. ***P < 0.001 for DCM-HF vs cntrl (Mann‒Whitney test). **l** ZFYVE27 protein levels normalized by ER-positive area. ***P < 0.001 for DCM-HF vs cntrl (Mann‒Whitney test). The results are reported as the mean ± SEM. **m**, **n** Activity of the ER/cholesterol-dependent signaling pathway was identified in living adult cardiomyocytes obtained from the atrial tissue of patients undergoing coronary artery bypass grafting. Cardiomyocytes were overloaded with 50 µM cholesterol for 45 min, and the ER was then detected in living cells. **m** Representative microphotographs. Scale bar, 20 µm. **n** Quantification of (**m**). To account for variability in cell size, the ER-positive area was normalized to the respective cell area. **P < 0.01 for cells treated with cholesterol vs. control vehicle (Mann‒Whitney test). Data are shown for *n* = 3 independent donors; *n* = 65 cells (control vehicle), *n* = 69 cells (cholesterol-treated). **o** Schematic model of pathological ER/cholesterol signaling due t**o** sarcomere disorganization in the presence of DCM mutations. In DCM (MUT) CMs, disrupted sarcomeres fail to form contact sites (denoted by stars) with the ER. Consequently, this results in defective ER architecture, diminished ER curvature, decreased levels of curvature-generating proteins, as well as dysregulated cholesterol (denoted by yellow circles) homeostasis. Ultimately, this molecular disease mechanism exacerbates compromised contractile function in patient-derived iPSC-CMs, which is already depressed due to sarcomere protein mutations. Our findings suggest this new pathomechanism to be active in the left ventricular tissue from patients suffering from dilated cardiomyopathy and end-stage heart failure. Correcting the DCM mutation via CRISPR/Cas9 in patient-specific iPSC-CMs or recovering cholesterol levels using pitavastatin rescues pathological ER/cholesterol signaling and improves contractile function in DCM mutation–carrying iPSC-CMs. A.u. indicates arbitrary units, cntrl control, DCM-HF dilated cardiomyopathy with end-stage heart failure, REEP5 receptor expression-enhancing protein 5, RTN4 reticulon-4, ATL3 atlastin-3, LNP ER junction formation protein lunapark, ZFYVE27 protrudin, KTN1 kinectin-1, RRBP1 ribosome-binding protein 1, CKAP4 cytoskeleton-associated protein 4, ACTN2 sarcomeric α-actinin, ER endoplasmic reticulum

We next tested whether the ER shape-modulating proteins in DCM-HF tissues would display a similar dysregulation to that observed in DCM iPSC-derived CMs relative to donor controls (Fig. 8a–i, Supplementary Fig. 15). Remarkably, when averaged among all DCM-HF patients, REEP5 was significantly diminished in the heart tissues from DCM patients (Fig. 8b), consistent with the results observed in DCM iPSC-CMs (Fig. 3h). However, interestingly, in the LV tissue of the patient DCM-HF8, who had received lipid-lowering therapy (simvastatin), REEP5 protein levels were not significantly different from those of donor controls (Supplementary Fig. 15c). While LNP and ZFYVE27 also exhibited significant changes in values in the overall DCM-HF group compared to controls (Fig. 8e, f), we found a strong variation between the tissues of DCM-HF patients (Supplementary Fig. 12e, h), reflecting the strong intra-individual variation observed in human disease models.^5,34^ Similar to observations in the DCM iPSC-CM model platform, the levels of the affected proteins were not significantly altered or even showed an increase after normalization to the relative ER-positive area (Fig. 8j–l).

We next sought to validate this newly identified mechanism of ER regulation through cholesterol signaling in human cardiac cells. We established a quantitative assessment of ER organization after cholesterol loading in living adult cardiomyocytes obtained from the right atrial appendages of three patients undergoing coronary artery bypass surgery. Clinical information for these patients is summarized in Supplementary Table 4. Our findings documented a reduced ER-positive area, normalized to cell area, in living adult CMs subjected to cholesterol treatment compared with vehicle controls (Fig. 8m, n). These experiments revealed that, strikingly, manipulating cholesterol levels offers a viable approach to modulate ER organization in living adult CMs isolated from adult patient tissue.

Overall, these findings recapitulate features of the newly described pathomechanism, dysregulated ER/cholesterol signaling (Fig. 8o), also in the heart tissues of DCM-HF patients. This confirms the clinical relevance of this new signaling pathway for the subgroup of patients with DCM due to inherited mutations in sarcomere proteins, offering new therapeutic avenues for DCM patients.

## Discussion

Despite improved treatments, dilated cardiomyopathy (DCM), a major cause of heart failure (HF) with high morbidity and mortality, remains a significant healthcare problem. Identifying novel molecular targets for pharmacological interventions in DCM may facilitate the development of new treatment strategies and improve patient outcomes.

Here, we revealed a novel molecular pathomechanism in DCM using a human patient-derived and iPSC-CM-based DCM platform. A key pathological feature of iPSC-CMs with inherited DCM-specific mutations is the abnormal organization of sarcomeres compared to WT controls.^5,12,34^ Here, utilizing biochemical experiments and transmission electron microscopy (TEM), we identified that sarcomere disorganization due to inherited DCM mutations leads to reduced sarcomere interactions with the ER, a nexus for intracellular signal transduction. Consequently, defective sarcomere/ER interactions result in loss of structural ER support and present a basis for severely disorganized ER architecture in DCM iPSC-CMs. Impaired ER organization was further confirmed by concomitantly reduced membrane curvature in DCM iPSC-CMs, as revealed by STED nanoscopy and nanoscale cryo-electron tomography (cryo-ET). In line with this, recent research has shown that ER curvature-generating proteins, such as REEP5, are critical for cardiac function.^42,43^ Using high-content protein analyses, we confirmed that the ER curvature proteins likely do not make a major contribution to the DCM molecular patho-phenotype. Instead, our findings suggest defective sarcomere/ER interactions due to disorganized sarcomeres in DCM to present a basis for the loss of ER membrane organization and architecture. Consequently, this results in an imbalance of lipid homeostasis, particularly of cholesterol. We confirmed this by ultrasensitive lipidomics analyses in iPSC-CMs, which revealed altered lipid profiles in DCM iPSC-CMs, specifically, elevated cholesterol levels. Our findings are supported by previous studies that suggested altered lipid metabolism in DCM patients.^9,44,45^ Here, we provide a potential mechanistic basis for these observations: defective ER/cholesterol signaling due to the disrupted sarcomeres in DCM patient cardiomyocytes not delivering structural support to the ER. Thus, sarcomere disorganization in DCM induces pathological ER/cholesterol signaling and exacerbates contractile dysfunction in DCM iPSC-CMs, as our findings show. Therefore, ER/cholesterol signaling is a critical modulator of subcellular pathological dysfunctions in DCM. Of note, we demonstrated the rescue of this new disease mechanism using two different strategies. First, CRISPR-Cas genomic editing of the DCM TPM1-L185F variant resulted in normalized sarcomere organization, recovered ER architecture, and normalized intracellular cholesterol levels. Next, the administration of cholesterol-lowering drugs to DCM (MUT) iPSC-CMs confirmed that correcting cholesterol levels is necessary and sufficient to restore ER membrane architecture. Importantly, we demonstrated that normalizing cholesterol levels significantly improved contractile function in DCM (MUT) iPSC-derived CMs. Previously, the use of methyl-β-cyclodextrin (MβCD), an acute cholesterol-reducing agent,^46^ was shown to increase twitch amplitude and the rates of shortening and relaxation as well as raise diastolic and systolic [Ca^2+^]I in isolated rat CMs.^47^ Here, we used pitavastatin, an effective HMG-CoA reductase inhibitor approved for clinical use in hyperlipidemia and familial hypercholesterolemia,^48^ and the squalene synthase inhibitor TAK-475 (lapaquistat acetate) to reverse defective ER/cholesterol signaling and deficient sarcomere contractile performance in DCM iPSC-CMs. Importantly, previous clinical trials that suggested that statins lack beneficial effects in patients with ischemic, systolic heart failure did not investigate subgroups of DCM-HF patients genotype-positive for inherited mutations causing DCM.^49–51^ However, other studies showed that clinical outcomes in DCM patients receiving statins were improved, including reduced end-diastolic and end-systolic left ventricular volumes, increased LVEF, reduced inflammation, and reduced mortality.^52,53^ Here, using a DCM patient-specific model platform, we showed that modulating cholesterol levels via pitavastatin significantly restored ER organization, cholesterol signaling, and ultimately contractile properties in DCM mutation–carrying iPSC-CMs. This is highly relevant, as reduced contractile function is a critical endpoint in DCM patients with HF. Moreover, consistent with previous studies,^54^ our findings suggest that cholesterol plays a role in modulating the ER membrane and its functions, which we reveal to be potentially conserved across different mammalian cell types.

We identified a new patho-mechanism triggered by sarcomere disorganization–dysfunctional ER/cholesterol regulation – in left ventricular (LV) samples obtained from DCM patients suffering from end-stage HF. These patients who received heart transplants, however, were not genetically tested for inherited DCM mutations. Our findings suggest that the high degree of sarcomere disarray in DCM-HF patient tissues is the prime driver of disrupted ER organization and defective cholesterol signaling in DCM patient tissues. Our findings imply that disorganized ER architecture also results in a significant imbalance of curvature-generating proteins such as REEP5 in DCM-HF tissues. This was further supported by the fact that no significant differences in REEP5 levels were observed following normalization of REEP5 values for the ER-positive intracellular area. Additionally, our study covered LV tissue samples from a DCM patient (DCM-HF8) who had received lipid-lowering therapy (simvastatin). While REEP5 was significantly diminished in the other DCM-HF tissues, REEP5 levels in DCM-HF8 were comparable to those of control donors, indicating that ER architecture may be restored at the molecular level following cholesterol modulation in patients. Importantly, using living atrial cardiomyocytes, we revealed that acute cholesterol loading may directly affect ER organization. Additional translational model systems, such as long-term cultured human myocardial slices,^55^ could assist future studies in further developing our findings toward additional preclinical evaluations of their clinical relevance. The newly identified mechanism could be validated by analyzing the contractile forces and intracellular calcium handling in slices isolated from failing hearts treated with drugs that lower cholesterol levels, such as statins and squalene synthase inhibitors, followed by subsequent analyses of ER architecture and function.

Together, our data suggest that the newly described pathomechanism, dysfunctional ER/cholesterol signaling, might be prevalent generally in the pathophysiology associated with defective sarcomere organization observed in DCM. We note that while statins are widely prescribed for preventing cardiovascular events in many diseases, including coronary artery disease and diabetes, they may induce myopathies in patients.^56^ Thus, fine-tuned drug regimens will be critical for the further preclinical evaluation of cholesterol-lowering medications as a novel treatment strategy in subgroups of DCM patients. Moreover, besides genetic cardiomyopathies, HF is also strongly associated with unfavorable lifestyle factors and conditions such as obesity, hypertension, and diabetes, which also result in altered lipid metabolism.^57^ Therefore, in future studies, genotype-positive groups of DCM patients should be investigated for defects in the ER/cholesterol signaling pathway. To further evaluate the clinical relevance and translational potential of our findings, future studies should focus on subgroups of DCM patients with familial sarcomere mutations but no confounding cardiac conditions, such as coronary artery disease. Therefore, our study further highlights the importance of genetic screening in DCM patients.^58^ However, other etiologies, such as ischemic DCM leading to concomitant sarcomere disorganization, could also display dysfunctional ER/cholesterol signaling, suggesting that these patient groups may also profit from therapeutic directions targeting this pathway.

Of note, alternative therapies to statins are currently under development.^59^ Cholesterol-lowering drugs, affecting not only liver- but also cardiac cholesterol production, include molecular blockers of lanosterol synthase and squalene synthase. In our study, we applied one of the known squalene synthase inhibitors, TAK475, to rescue pathological ER organization and concomitant cholesterol imbalance-related defective signaling. Our findings suggest that squalene synthase inhibitors may present promising candidates for correcting defective ER/cholesterol functions caused by disorganized sarcomeres due to inherited DCM mutations. Therefore, we propose that biochemical modulation reversing pathological ER/cholesterol signaling in DCM patients may present a promising direction for future clinical treatment strategies in DCM.

## Methods

### Ethics statements

The protocols involving iPSC studies were approved by the Goettingen University Ethical Board (No. 7/5/24, 15/2/20, 20/9/16An) and the Odense University Ethical Board (Projekt ID S-20140073HLP). Informed consent was obtained from all participants, and all research was performed in accordance with relevant guidelines and regulations. Approval for the study of human myocardial samples was granted by the Goettingen University Ethical Board (No. 21/10/00 and 31/9/00), and written informed consent was obtained from all patients. For all patient samples used for human atrial myocyte isolation, all experimental protocols were authorized by the Ethics Committee of the University Medical Center Göttingen (No. 4/11/18). The procedures used in this study adhere to the tenets of the Declaration of Helsinki.

### Generation, culture and CRISPR/Cas9 genome editing of human iPSCs

The investigation conforms to the principles outlined in the Declaration of Helsinki. The institutional ethics committee approved the study. Informed consent was obtained from all participants, and all research was performed in accordance with relevant guidelines and regulations. The following abbreviations were used: DCM patient-specific TPM1-L185F iPSC – PAT1, corresponding family control – WT1, WT1 iPSC containing CRISPR-Cas9-introduced TPM1-L185F-mutation – MUT1. DCM patient-specific TnT-R173W iPSC – PAT2, corresponding family control – WT2, WT2 iPSC containing CRISPR-Cas9-introduced TnT-R141W-mutation – MUT2. Patient-specific TPM1-L185F (PAT1) and family control iPSCs (WT1) were generated before^5^ by non-integrating reprogramming of fibroblasts using the CytoTuneTM-iPS 2.0 Sendai Reprogramming Kit (Thermo Fisher Scientific, USA). The patient-specific TnT-R173W iPSC line (PAT2) and family control iPSC line (WT2) were a kind gift from Joseph C. Wu (Stanford University, USA).^3^ To generate iPSC lines containing isogenic homozygous mutations (TPM1-L185F, MUT1; TnT-R141W, MUT2), as well as iPSCs from a DCM patient in which the TPM1-L185F mutation was corrected (PAT1-COR), CRISPR/Cas9 editing via homology-directed repair was applied as described.^5,13^ In short, iPSCs were subjected to transfection of new sequences subcloned and inserted into the pSpCas9(BB)-2A-Puro plasmid together with sgRNAs and introduced into iPSCs using a cell electroporation system (Lonza, Switzerland). Single clones were isolated and sequence verified. Human iPSCs were cultured at 37 °C with 5% (vol/vol) CO_2_ on Matrigel (BD Biosciences, USA)-coated plates using chemically defined Essential 8 medium^60^ that was refreshed daily. Human iPSCs were dissociated with EDTA and passaged every four days (Life Technologies, USA).

### Differentiation of human iPSCs to cardiomyocytes

Cardiac differentiation from patient-specific iPSCs and from CRISPR/Cas9-engineered iPSCs was conducted via a small molecule-based monolayer protocol described elsewhere.^12,61–63^ In brief, the GSK inhibitor CHIR 99021 (Selleckchem, USA) was applied for 24–48 h, followed by modulation of the Wnt signaling pathway using IWR1 (Selleckchem). Starting from day 7 of cardiac differentiation, beating cardiomyocytes were observed. Human iPSC-derived CMs were cultured in RPMI medium supplemented with B-27 (Thermo Fisher Scientific) at 37 °C with 5% (vol/vol) CO_2_. On days 20–25 of cardiac differentiation, beating iPSC-CMs were dissociated using trypleE (Life Technologies) and plated in suitable assay formats.

### Lipidomic analyses

Human iPSC-CM pellets (ca. 1 million cells per technical replicate) were subjected to acidic liquid‒liquid extraction and lipidomics analyses according to previous reports.^15,64,65^ Prior to extraction, internal lipid standards were added using a master mix containing 100 pmol deuterated cholesterol (D6-cholesterol or D7-cholesterol, Cambridge Isotope Laboratory, UK) (all standards are listed in ref. ^15^). A test extraction was performed to estimate the concentration of the bulk membrane lipid species phosphatidylcholine. Sample volumes were adjusted to obtain similar total lipid concentrations for different samples that were used for extraction and to ensure a linear range of standard-to-endogenous lipid ratios. Within these criteria, endogenous lipid species were calculated relative to internal standards. Lipid extracts were resuspended with 10 mM NH_4_CH_3_COO in MeOH before measurement. For cholesterol determination, extracts were evaporated and acetylated as reported.^66^ Extracts were infused and ionized using electrospray technology (Triversa Nanomate (Advion Biosciences, USA) and analyzed with LC‒MS/MS System QTRAP5500 (Sciex, USA) (for cholesterol determination). For other lipid species, QTRAP6500+ (Sciex) was employed. Quantification of endogenous lipids was conducted with LipidView (Sciex) and homemade ShinyLipids software.

### Measurements of plasma membrane cholesterol levels

Human iPSC-derived CMs were seeded onto 10-mm glass coverslips (Epredia, USA) 3–4 days before the experiment. Purified recombinant GFP-tagged D4 domain of perfringolysin O (PFO, θ-toxin) (a kind gift from Volker Haucke, Berlin, Germany)^15^ was applied to live iPSC-CMs (6 µg/ml) for 15 min at 37 °C. Coverslips were fixed with 4% PFA (Thomas Scientific) in PBS, mounted with FluroMount medium (Thermo Fisher Scientific) and imaged using a confocal microscope (Carl Zeiss, LSM 710, Goettingen, Germany) equipped with a 63× plan apochromat oil immersion objective using ZEN software (Carl Zeiss). Relative fluorescence units (RFUs) were calculated in ImageJ as the mean gray value of the region of interest (ROI) corresponding to the plasma membrane outlines, following the subtraction of background fluorescence intensity for individual cells.

### High-content protein mini-screening

Human iPSC-CM lysates were applied directly onto Immobilon PVDF membranes (Merck, Germany) as described.^67^ Membranes were incubated with primary antibodies overnight at 4 °C. To study cholesterol biosynthesis and turnover enzymes, we used antibodies against HMGCR (13533-1-AP, Proteintech), LSS (13715-1-AP, Proteintech), FDFT1 (13128-1-AP, Proteintech), ACAT1 (35695, Cell Signaling), and phospho-HMGCR (Ser872) (BS-4063R, Thermo Fisher Scientific). To study ER-shaping proteins, we used antibodies against REEP5 (14643-1-AP, Proteintech), RTN4 (10950-1-AP, Proteintech), ATL3 (16921-1-AP, Proteintech), RRBP1 (22015-1-AP, Proteintech), KTN1 (19841-1-AP, Proteintech), ZFYVE27 (12680-1-AP, Proteintech), CKAP4 (16686-1-1AP, Proteintech) and LNP (80637, Novus Biologicals, USA). GAPDH antibody (sc-47724, Santa Cruz Biotechnology) was used as a loading control. The secondary antibodies IRDye 680RD donkey anti-rabbit IgG or IRDye 680RD goat anti-mouse IgG (LI-COR Biosciences, USA) were added afterwards. Membranes were scanned and quantified with an Odyssey XF Imaging System (LI-COR Biosciences) and Image Studio Lite Ver 5.2. software. Normalization of protein levels to GAPDH levels was performed.

### Immunohistochemistry and confocal imaging

Human iPSC-derived CMs were cultured on 10-mm glass coverslips (Epredia) 3–4 days before fixation with 4% paraformaldehyde (PFA) (Thomas Scientific) in PBS (Gibco, USA) or 4% glyoxal (Sigma‒Aldrich, Germany).^68^ The cells were permeabilized with 0.2% Triton X-100 (Carl Roth, Germany) in PBS and blocked with 5% BSA (Sigma‒Aldrich) in PBS at room temperature. Incubation with primary antibodies was conducted overnight at 4 °C. Antibodies against sarcomeric α-actinin (ACTN2, 14221-1-AP, Proteintech, USA) and tropomyosin-1 (TPM1, ab7785, Abcam, UK) were used for assessment of iPSC-CM subcellular organization. Antibodies against Sec61β (rabbit, a kind gift from Blanche Schwappach, Goettingen, Germany) or KDEL (sc-58774, mouse, Santa Cruz Biotechnology, USA) were used to label the ER. Secondary antibodies (Alexa Fluor 488 or 568 goat anti-rabbit or goat anti-mouse; Thermo Fisher Scientific) were subsequently used. Slides were mounted using Fluoromount-G (Thermo Fisher Scientific). Confocal images were captured as described above. The organization of human iPSC-CMs was assessed using immunofluorescence imaging for tropomyosin (TPM1) and cardiac α-actinin (ACTN2). Three independent, blinded operators evaluated the images according to standardized criteria. Cardiac cells exhibiting well-distributed sarcomeres with typical protein arrangement throughout the cell, along with regular fluorescence levels of TPM1 and ACTN2, were classified as organized. Cells were classified as “disorganized” if more than 50% of visible sarcomeres lacked the characteristic striated pattern, displayed disrupted Z-line alignment, irregular spacing, or showed an absence of sarcomeres in the central region of the cell. Cells that could not be clearly categorized as organized or disorganized, were only partially visible, or displayed nonstandard sizes were excluded from the analysis. ER-positive intracellular area was measured in ImageJ as follows. Individual cells were selected and outlined manually. An automated threshold setting was applied to the Sec61β or KDEL channel in ImageJ; afterwards, Sec61β- or KDEL-positive regions of interest (ROIs) were defined, and their area was quantified. ER distribution was measured as a percentage of average Sec61β relative fluorescence values along different intracellular compartments. Analysis of the ER junctions and branch sites, branching interval and length, and ER total length was performed in ImageJ using a previously published automated tool for the quantitative assessment of the tube networks.^21,22^

### STED imaging

Human iPSC-CMs were subjected to immunostaining with the KDEL antibody, followed by incubation with a secondary anti-mouse Abberior STAR 635 P antibody (Abberior GmbH, Germany). Slides were then mounted using Fluoromount medium (Thermo Fisher Scientific), and images were captured using a STED microscope (Leica, TCS SP5, Goettingen, Germany) equipped with a 100× plan apochromat oil immersion STED objective and Leica LAS AF imaging software as described.^68^

### Cryo-electron microscopy and ER curvature analysis

The EM grids (Au 200 mesh R1.2/20 SiO_2_ support (Quantifoil Micro Tools GmbH)) were plasma cleaned (NanoClean 1070) with an oxygen and argon mix (ratio 25:75) at 100% power for 2 min. Then, the EM grids were coated with Matrigel before human iPSC-CMs were seeded with a cell density of 0.2 × 10^6^ cells in 35 mm Petri dishes. The cells on EM grids were incubated for 3–4 days to allow them to firmly attach to the EM grid support before plunge freezing. The grids were plunged into liquid ethane with a Leica GP2 plunger (Leica Microsystems). The frozen grids were clipped into custom-made AutoGrid cartridges modified for focused ion beam scanning electron microscopy milling. The clipped AutoGrids were screened for ice thickness on a 200 kV Cryo-TEM Glacios (Thermo Fisher Scientific). Lamellae were prepared with cryo-FIB-SEMs (Crossbeam 550 Zeiss equipped with Quorum cryo-stage or ThermoFisher Scientific Aquilos2). Tilt series for the images shown in Fig. 3c–f were collected on Titan Krios G4 (Thermo Fisher Scientific) equipped with a cold-field emission gun, Selectris X energy filter, and Falcon 4 camera. Data acquisition was performed with serialEM software at 33000× magnification, producing a tilt series of 4096 × 4096 pixels with a nominal pixel size of 3.77 Å. The tilt series collection scheme was dose-symmetric with a pretilt of −12° (which fits the milling angle). The tilt step was 2° and the tilt range was −60° to +48°, with a total accumulated dose of 120 e − /Å^2^ for each tilt series. The target defocus range was −3 µm to −5 µm. Reconstructed tomograms were acquired via IMOD software.^69^ Analysis of ER membrane curvature was performed by profiling tangential curvature along individual ER tubules in a semi-automated manner using reconstructed cryo-ET tomograms and an open-source Fiji plugin, Kappa - Curvature analysis (version 1.7.3). A single z-stack from each tomogram containing a clearly defined ER structure was opened via the plugin. Using a point-click tool, we created initialization open B-spline curves following curved ER membrane regions (typically 1–2 μm long). The curve was then fitted to the membrane contour by the Kappa module using a minimization algorithm. Computed averaged data points (μm⁻¹) for each curve were extracted from the plugin and plotted.

### Co-immunoprecipitation and western blotting

CKAP4-specific antibody (16686-1-1AP, rabbit, Proteintech) was coupled to Protein G Sepharose 4 Fast Flow beads (Cytiva, USA) as recommended by the manufacturer. Non-decorated beads were used as negative controls. Human iPSC-CM lysates were prepared for immunoprecipitation binding with IP buffer (25 mM Tris, 150 mM NaCl, 1 mM EDTA, pH = 7.5, with Roche protease inhibitor cocktail (Sigma‒Aldrich)). Eluted protein solutions and lysate inputs were subjected to SDS‒PAGE and transferred to Immobilon PVDF membranes (Merck). Membranes were incubated with primary antibodies against CKAP4 (16686-1-1AP, rabbit, Proteintech), MYH7 (22280-1-AP, rabbit, Proteintech), SAA (14221-1-AP, rabbit, Proteintech), TPM1 (ab7785, mouse, Abcam), troponin T (MS-295-P1, mouse, Epredia), GAPDH (sc-47724, Santa Cruz Biotechnology), and LI-COR Biosciences secondary antibodies for the sarcomere-ER interaction study. Signals were detected and quantified via an Odyssey XF Imaging System (LI-COR Biosciences) and Image Studio Lite Ver 5.2. Representative membrane scans for individual proteins are shown.

### Transmission electron microscopy

Human iPSC-CMs were fixed with 2% formaldehyde and 2.5% glutaraldehyde, post-fixed with 1% OsO_4_ and embedded in the culture dish in Epon resin after en bloc staining as detailed previously.^70^ Ultrathin sections were produced with an UC7 Ultramicrotome and stained with UranyLess Contrast stain solution. Transmission electron micrographs were obtained using LEO EM912 (Carl Zeiss Microscopy GmbH, Oberkochen, Germany). Quantification of the sarcomere length, sarcomere-ER distance and number of contact sites was performed using ImageJ. Myofibrils were identified based on periodic electron-dense Z-lines, and ER tubules were visually traced. The sarcomere-ER distance was defined as the shortest straight line between the Z-line of a sarcomere and the ER tubular domain when present in close apposition (≤0.8 µm). A single linear region of interest (ROI) was defined per Z-line. Interaction sites were defined as discrete contact points where ER tubules were closely aligned (≤0.3 µm) with sarcomeres. For each field of view, the number of interaction sites along individual myofibrils was counted and normalized to myofibril length.

### Drug treatments

Human iPSC-derived CMs were cultured on 10-mm coverslips (Epredia) 2 days before the experiment and treated either with water-soluble cholesterol (Sigma‒Aldrich), DL-mevalonolactone (the δ-lactone of mevalonic acid, Santa Cruz Biotechnology), pitavastatin calcium (Santa Cruz Biotechnology), or TAK-475 (Hycultec, Germany) for 24 h. For sarcomere-ER colocalization studies, human iPSC-CMs were treated with 200 nM pitavastatin calcium for 72 h. Water-soluble cholesterol contains 40 mg of cholesterol per gram of material, balanced with methyl-β-cyclodextrin (MβCD); concentrations used for treatments were calculated based on the amount of pure cholesterol.

### Assessment of contractile function

Human iPSC-derived CMs were seeded onto 10-mm coverslips (Epredia) 5 days before the measurements and transferred to imaging chambers (ibidi, Germany) 3 days before the measurements. Recording was performed using an Olympus microscope and Olympus IX2-UCB software (frame rate of 50 frames/second). Raw data were processed with the Python non-negative blind deconvolution-based method as reported earlier.^13^ Alternatively, iPSC-CM contractility was assessed using an IonOptix MyoCam system and IonWizard 7.8 software (IonOptix LLC, USA) in line with previous reports.^71–73^

### Human myocardial tissue preparation, lipidomics, and immunoblotting

The investigation conforms to the principles outlined in the Declaration of Helsinki. The institutional ethics committee approved the study, and all patients provided written informed consent for the use of cardiac tissue samples. Clinical data for DCM patients with end-stage heart failure (HF) who received a heart transplant are presented in supplementary Table 3. Patient DCM-HF8 received 40 mg simvastatin once a day in the evening. Left ventricular (LV) tissues from healthy donor hearts that had failed transplantation served as controls and were described earlier.^40^ Healthy human hearts (no pre-existing cardiac disease and noncardiac reason for death) were initially planned for transplantation but were ultimately rejected due to organ mismatch, elevated serum C-reactive protein levels, and local hypokinesia. Heart biopsies were placed in a cardioprotective solution, and LV tissue samples were excised as detailed in a prior study and stored at -80°C. Human heart tissue chunks (ca. 1 mg per technical replicate) were subjected to lipidomics analyses as described above for iPSC-CM samples. To perform immunoblotting, we subjected LV heart tissues to homogenization and lysis, as described.^5^ Supernatants were applied onto Immobilon PVDF membranes (Merck) as previously described.^67^ Membranes were incubated overnight at 4 °C with primary antibodies against REEP5 (14643-1-AP, Proteintech), RTN4 (10950-1-AP, Proteintech), ATL3 (16921-1-AP, Proteintech), RRBP1 (22015-1-AP, Proteintech), KTN1 (19841-1-AP, Proteintech), ZFYVE27 (12680-1-AP, Proteintech), CKAP4 (16686-1-1AP, Proteintech) and LNP (80637, Novus Biologicals, USA). ACTN2 antibody (14221-1-AP, Proteintech) was used as a cardiac marker. GAPDH antibody (sc-47724, Santa Cruz Biotechnology) was used as a loading control. The following secondary antibodies were used: IRDye 680RD donkey anti-rabbit IgG or IRDye 680RD goat anti-mouse IgG (LI-COR Biosciences). Membranes were scanned and analyzed with an Odyssey XF Imaging System (LI-COR Biosciences) and Image Studio Lite Ver 5.2. software. Normalization to GAPDH levels was performed.

### Human myocardial tissue immunostaining

The investigation conforms to the principles outlined in the Declaration of Helsinki. The institutional ethics committee of Goettingen University Medical Center approved the study, and all patients provided written informed consent for the use of cardiac tissue samples. Adult human heart tissue samples were prepared and stored at −80 °C before embedding, as described above. Clinical data for DCM patients with end-stage HF are presented in supplementary Table 3. The LV tissue samples were immunostained following a published protocol.^74^ Briefly, the samples were mounted in Tissue-Tek OCT Compound (Sakura Finetek, USA). Then the samples were cut into 4-µm sections. Tissue slices were fixed with ice-cold acetone, permeabilized with 0.2% Triton X-100 (Carl Roth) in PBS, blocked with 5% BSA (Sigma‒Aldrich) in PBS and incubated with the primary antibody against Sec61β (15087-1-AP, Proteintech) and the secondary antibody Alexa Fluor 488 goat anti-rabbit (Thermo Fisher Scientific). Cell nuclei and plasma membranes were visualized with DAPI (Sigma‒Aldrich) and wheat germ agglutinin (WGA) Alexa Fluor 594 conjugate (Thermo Fisher Scientific), respectively. Slides were mounted with Fluoromount-G (Thermo Fisher Scientific). Staining specificity for the Sec61β rabbit antibody was confirmed using a primary IgG rabbit antibody (2729S, Cell Signaling Technology, USA) or a secondary Alexa Fluor 488 goat anti-mouse antibody (Thermo Fisher Scientific) (Supplementary Fig. 14). An LSM 710 microscope (Carl Zeiss, Goettingen, Germany) with a 63× plan apochromat oil immersion objective and ZEN software (Carl Zeiss) was employed to acquire confocal images of LV tissues derived from control donors and DCM-HF patients. Analysis was performed using ImageJ. The WGA channel was used to identify individual cardiomyocytes. An automated threshold setting was applied for the Sec61β channel to determine and measure the Sec61β-positive area for individual cells.

### Human adult atrial myocyte isolation and cholesterol overloading

For all patient samples, all experimental protocols were authorized by the ethics committee of the University Medical Center Göttingen (No. 4/11/18) and performed following the Declaration of Helsinki. Right atrial appendages were excised as part of routine right atrial cannulation for extracorporeal circulation in patients who were receiving open-heart coronary artery bypass grafting (CABG). Corresponding clinical information can be found in Supplementary Table 4. The excised tissues were transported in a sterile cardioplegic-based solution (30 mM 2,3-butanedione monoxime, 20 mM glucose, 10 mM KCl, 1.2 mM KH_2_PO_4_, 5 mM MgSO_4_, 5 mM MOPS, 100 mM NaCl, 50 mM taurine; pH 7.0) for subsequent atrial myocyte isolation with a previously established protocol.^75^ In brief, the atrial samples were trimmed of adipose material and finely cut into fragments approximately 1 mm³ in size in a pre-cooled calcium-free buffer (20 mM glucose, 50 mM taurine, 100 mM NaCl, 10 mM KCl, 1.2 mM KH_2_PO_4_, 5 mM MgSO_4_, 5 mM MOPS; pH 7.4). The minced tissue was incubated in a calcium-free solution containing collagenase type I (286 U/mL, Worthington) and protease XXIV (5 U/mL, Sigma‒Aldrich) enzymes for digestion for 45 min at 37 °C in a jacketed beaker and maintained under continuous bubbling of 100% O₂. 10 min after the start of digestion, CaCl₂ was added to a final concentration of 20 μM. After 45 min, digestion was continued with a newly prepared 20 mL solution containing collagenase type I (286 U/mL) and 20 μM CaCl₂. Dissociated myocytes were monitored microscopically by inspecting the supernatant every 3 min. Once myocytes began to dissociate, the remaining tissue was gently triturated in storage buffer to release further cells. The supernatant was passed through a nylon mesh to remove debris, followed by centrifugation at 90 × *g* for 7 min. The pelleted myocytes were then resuspended in a newly prepared storage buffer (1% (w/v) albumin, 10 mM glucose, 10 mM taurine, 10 mM β-hydroxybutyric acid, 70 mM L-glutamic acid, 20 mM KCl, 10 mM KH₂PO₄, pH adjusted to 7.4 with 1 M KOH). The isolated myocytes were subsequently resuspended in RPMI medium plus B-27 supplement (Thermo Fisher Scientific), incubated with 50 µM water-soluble cholesterol (Sigma‒Aldrich) for 45 min and stained with the ER staining kit Cytopainter (Abcam) according to the manufacturer’s instructions.

### Statistical analysis

Cell biology and biochemical experiments, including immunoblotting, co-immunoprecipitation, immunohistochemistry, and image analysis, were conducted in an unblinded manner for *n* ≥ 2 independent experiments to ensure reproducibility, with each experiment corresponding to a separate iPSC cardiac differentiation unless otherwise specified. Statistical analyses were performed using GraphPad Prism 9.4.1. The Shapiro‒Wilk test was employed to assess Gaussian distribution. For two-group comparisons, an unpaired t-test was used for normally distributed data, while the Mann‒Whitney test was used for non-normally distributed data. Statistical differences among more than two groups were assessed by an ordinary one-way ANOVA followed by Tukey’s multiple comparison test (for normally distributed data) or Kruskal‒Wallis test followed by Dunn’s multiple comparisons test (for non-normally distributed data). *P* < 0.05 were considered statistically significant. Data are presented as the mean ± standard error of the mean (SEM).

## Supplementary information

Ignatyeva et al, supplemental materials

Supplemental Movie S1

Supplemental Movie S2

Supplemental Movie S3

Supplemental Movie S4

Supplemental Movie S5

Supplemental Movie S6

Supplemental Movie S7

Supplemental Movie S8

Supplemental Movie S9

Supplemental Movie S10

Supplemental Movie S11

Supplemental Movie S12

Supplemental Movie S13

Supplemental Movie S14

Supplemental Movie S15

Supplemental Movie S16

Data S1

## Data Availability

The authors declare that all data generated in this study are included in the published article and its Supplementary Data files. Additional methods are available in the Supplementary Material. Raw cryo-electron tomography data have been deposited in the figshare public repository (10.6084/m9.figshare.31677724).
