# Supplementary material for: Targeting intracellular cholesterol imbalance rescues sarcomere–ER contact site signaling and ER remodeling in dilated cardiomyopathy
Source: Signal Transduct Target Ther. 2026 Jun 17;11:237. doi: 10.1038/s41392-026-02731-3 (PMC13273072; doi:10.1038/s41392-026-02731-3)
Supplement: Supplementary file 1 — Ignatyeva et al, supplemental materials [file 41392_2026_2731_MOESM1_ESM.docx]

Supplementary Materials for

Targeting intracellular cholesterol imbalance rescues sarcomere–ER contact site signaling and ER remodeling in dilated cardiomyopathy

Nadezda Ignatyeva^1,2^, Cleophas Cheruiyot^1,2^, Hafiza Nosheen Saleem^1,2^, Ruheen Wali^1,2^, Daria Plota^1,2^, Wenjing Zhang^1,2^, Sophie Schön^1,2^, Soeren Brandenburg^1,2,3^, Zhengyi Yang^4^, Anna Steyer^4^, Henning Urlaub^5,6^, Torsten Rasmussen^7^, Jens Mogensen^8^, Julius Ryan D. Pronto^2,9^, Yannic Döring^2,9^, Michael H. Radke^10,11^, Halyna Shcherbata^12,13^, Stephan E. Lehnart^1,2,14^, Andreas Janshoff^15,16^, Samuel Sossalla^1,3,17^, Torben Ruhwedel^18^, Wiebke Moebius^14,18^, Karl Toischer^1,2,3^, Britta Brügger^19^, Volker Haucke^20,21,22^, Michael Gotthardt^22,23,24^, Niels Voigt^2,9,14^, Antje Ebert^1,2,*^

Correspondence to: [antje.ebert@med.uni-goettingen.de](mailto:antje.ebert@med.uni-goettingen.de)

**This PDF file includes:**

Materials and Methods

Figures S1 to S15

Tables S1 to S4

**Other Supplementary Materials for this manuscript include the following:**

Movies S1 to S16

Data S1

Materials and Methods

Characterization of iPSCs

The protocols involving iPSC studies were approved by the Goettingen University Ethical Board (No. 7/5/24, 15/2/20, 20/9/16An) and the Odense University Ethical Board (Projekt ID S-20140073HLP). Informed consent was obtained from all participants, and all research was performed in accordance with relevant guidelines and regulations. Human iPSCs were characterized in line with our previously reported methods ^1-3^. Human iPSCs were plated on 10 mm glass coverslips (Epredia, USA) 3-4 days before the experiment. Cells were fixed with 4% PFA (Thomas Scientific) in PBS (Gibco, USA), permeabilized with 0.3% Triton X-100 (Carl Roth, Germany) in PBS for 30 min and blocked with 5% BSA (Sigma-Aldrich) in PBS at room temperature. Incubation with primary antibodies was conducted overnight at 4°C. Primary antibodies were against stem cell markers Oct3/4 (POU5F1, polyclonal goat IgG (AF1759-SP), R&D Biosystems) and podocalyxin (TRA-1-81, mouse IgM (09-0011), Stemgent Pelobiotech). Secondary antibodies Alexa Fluor 568 donkey anti-goat IgG (Thermo Fisher Scientific) and Alexa Fluor 488 rat anti-mouse IgM 406521 (Biolegend, USA) were added afterwards. DAPI was used to stain cell nuclei. Slides were mounted with FluroMount medium (Thermo Fisher Scientific). Images were acquired using a confocal microscope (Carl Zeiss, LSM 710, Goettingen, Germany) equipped with 63× plan apochromat oil immersion objective using ZEN software (Carl Zeiss). Western blotting was performed using an antibody against Oct3/4 (goat AF1759-SP, Novus Biologicals). GAPDH was used as a loading control.

Characterization of iPSC-CMs

Human iPSCs-CMs were characterized as we reported previously ^1-3^. Human iPSC-CMs were subjected to immunostaining, as described above, using antibodies against cardiac markers troponin T (TNNT2, MS-295-P1, Epredia) and sarcomeric α-actinin (ACTN2, 14221-1-AP, Proteintech), or myosin heavy chain 7 (MYH7, 22280-1-AP, Proteintech). Western blotting was performed using the antibodies against myosin regulatory light chain 2 (MLC2a, 311 011AT1, Synaptic Systems), troponin T (TNNT2, MS-295-P1, Epredia), and tropomyosin 1 (TPM1, ab7785, Abcam). GAPDH was used as a loading control.

Immunostaining and co-localization analysis

Human iPSC-CMs were subjected to immunostaining as described above, using CKAP4 antibody (16686-1-1AP, Proteintech) in combination with ACTN2 (Sigma, A7811) antibody for sarcomere-ER colocalization studies. SERCA2 ATPase antibody (Abcam) in combination with KDEL (10С3) antibody (sc-58774, SantaCruz), or SERCA2A antibody (sc-376235, SantaCruz) in combination with REEP5 antibody (Proteintech) were used for SR/ER colocalization studies. Subsequent steps, including incubation with secondary antibodies and STED imaging, were conducted as described above. Quantification of Manders’ coefficients for SR/ER colocalization (supplementary Fig. 6) was performed via ImageJ plugin “Colocalization Threshold”. Quantification of Manders’ coefficients for CKAP4/ACTN2 colocalization (supplementary Fig. 13) was performed via ImageJ plugin “JACoP BIOP” ^4^.

Cell viability assay

Human iPSC-CMs were passaged 2 days prior to treatment with 2 μM and 10 μM cholesterol-methyl-β-cyclodextrin 24 h or 48 h, 1 mM and 10 mM mevalonolactone for 24 h or 48 h, or 50 nM and 200 nM pitavastatin calcium for 24 h or 72 h. Cell imaging was performed using ECLIPSE Ts2 inverted microscope (Nikon Instruments, USA), Basler acA2000-50gm camera and Pylon Viewer software (Basler, Germany). Cell viability was analyzed using a LUNA-II automated cell counter (Logos Biosystems, South Korea) according to manufacturer's instruction.

Cryo-electron microscopy

Human iPSC-CMs were seeded onto glow-discharged gold EM grids (Au 200 mesh, R1.2/20 SiO₂ support film; Quantifoil Micro Tools GmbH, Germany). After a two-day recovery, the cells were drug-treated with either 2 µM water-soluble cholesterol (Sigma-Aldrich) or 200 nM pitavastatin calcium (Santa Cruz Biotechnology) for 24 h. The grids were then plunge-frozen using a Leica GP1 automatic plunge freezer and stored in liquid nitrogen until further analysis. The grids were screened for cell distribution and sample thickness on a Titan Krios G1 (Thermo Fisher Scientific) with a Cs-corrector and a 967 BioQuantum Special energy filter and K2 camera (Gatan) using EPU (Thermo Fisher Scientific). The cells were milled with a Cryo-Focused-Ion-Beam Scanning Electron Microscopy (Cryo-FIB-SEM) Aquilos 2 (ThermoFisher Scientific). Two layers of platinum were deposited on the grid to protect the sample and ensure conductivity. Lamellae were milled with gallium ion beam, current 3 nA-30 pA, to a thickness of approximately 250 nm, which allowed for collection of cryo-ET data of a cellular volume slice with good contrast. Tilt series data for the images shown in supplementary Fig. 7, supplementary Fig. 9, and supplementary Fig. 12g-h was collected using a Titan Krios G3i (Thermo Fisher Scientific) with an X-FEG and a BioQuantum energy filter + K3 camera (Gatan), controlled by the Tomography software (Thermo Fisher Scientific) at a magnification of 26.000× (5760 × 4092 pixels, nominal pixel size = 3.4 Å). A dose symmetric data collection scheme was used from a pre-tilt depending on milling angle (10-15°), and +/- 48° tilt range in steps of 2°, for a total dose of ~120 e/Å^2^. Reconstructed tomograms and 3D models were generated using IMOD software ^5^.

References

1. Ebert, A. D. et al. Characterization of the molecular mechanisms underlying increased ischemic damage in the aldehyde dehydrogenase 2 genetic polymorphism using a human induced pluripotent stem cell model system. *Sci. Transl. Med.* **6**, 255ra130 (2014).
2. Dai, Y. et al. Troponin destabilization impairs sarcomere–cytoskeleton interactions in iPSC-derived cardiomyocytes from dilated cardiomyopathy patients. *Sci. Rep.* **10**, 209 (2020).
3. Dai, Y. et al. An alternative mechanism of subcellular iron uptake deficiency in cardiomyocytes. *Circ. Res.* **133**, e19–e46 (2023).
4. Bolte, S. & Cordelières, F. P. A guided tour into subcellular colocalization analysis in light microscopy. *J. Microsc.* **224**, 213–232 (2006).
5. Kremer, J. R., Mastronarde, D. N. & McIntosh, J. R. Computer visualization of three-dimensional image data using IMOD. *J. Struct. Biol.* **116**, 71–76 (1996).


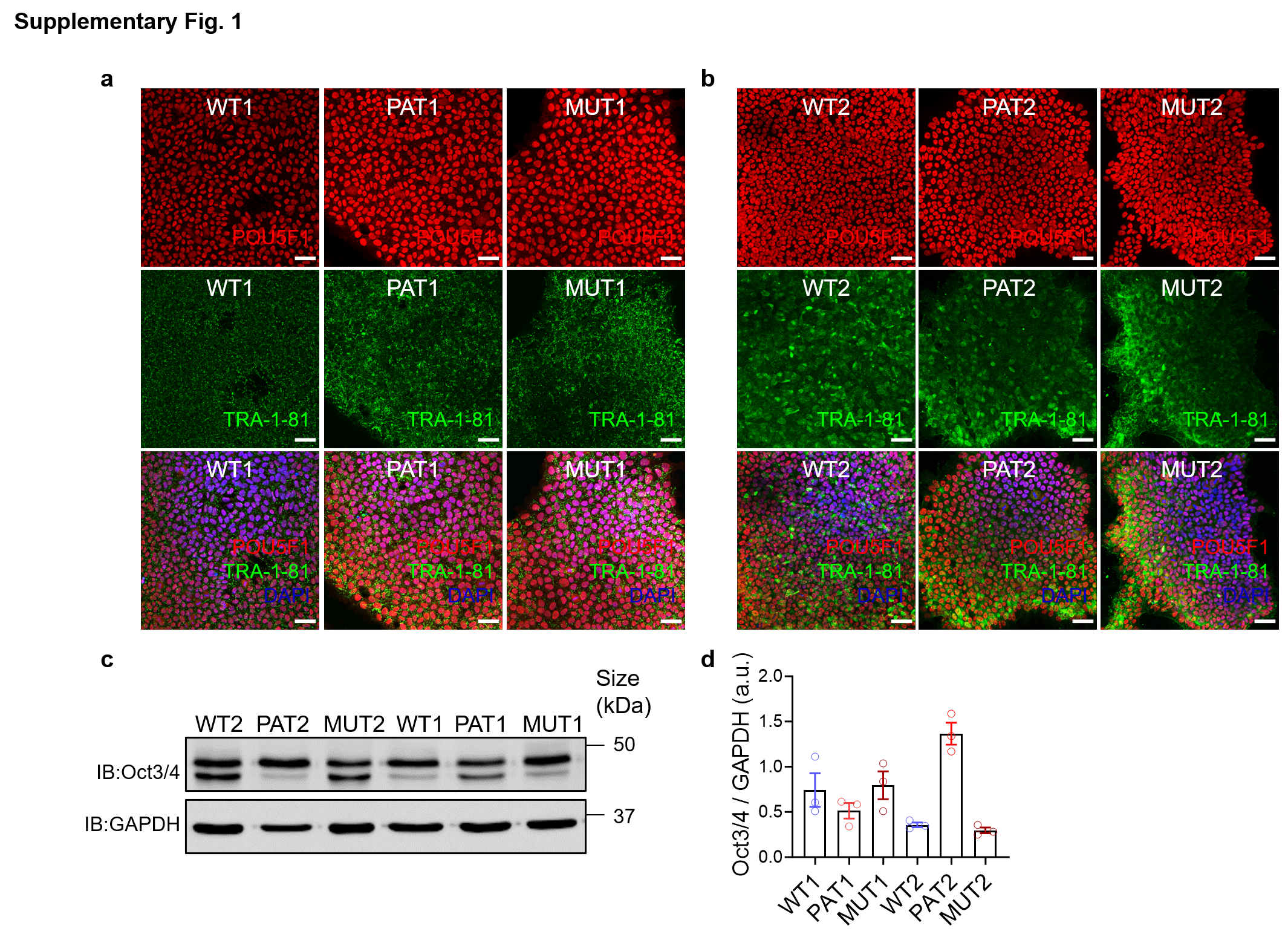
Figure. S1.

Characterization of DCM patient-specific and Cas9 mutation-introduced iPSCs versus WT controls. DCM (PAT1, PAT2, MUT1, MUT2) iPSCs and WT (WT1, WT2) iPSCs show regular levels of pluripotency markers. **a-b**, Representative confocal images following POU5F1- and TRA-1-81-specific immunostaining together with DAPI staining. Scale bar, 50 µm. **c-d**, iPSCs display comparable protein levels of pluripotency marker Oct3/4. c, Representative membrane scans for Oct3/4 immunoblotting. **d**, Quantification of c. Data are normalized by GAPDH. Per group, n=1 experiment, n=3 technical replicates. Data are presented as the mean±SEM. A.u. indicates arbitrary units; PAT1, DCM patient-specific TPM1-L185F iPSC; PAT2, DCM patient-specific TnT-R173W iPSC; MUT1, iPSC containing CRISPR/Cas9-introduced TPM1-L185F-mutation; MUT2, iPSC containing CRISPR/Cas9-introduced TnT-R141W-mutation; WT, wild-type; POU5F1, POU class 5 homeobox 1; TRA-1-81, podocalyxin.


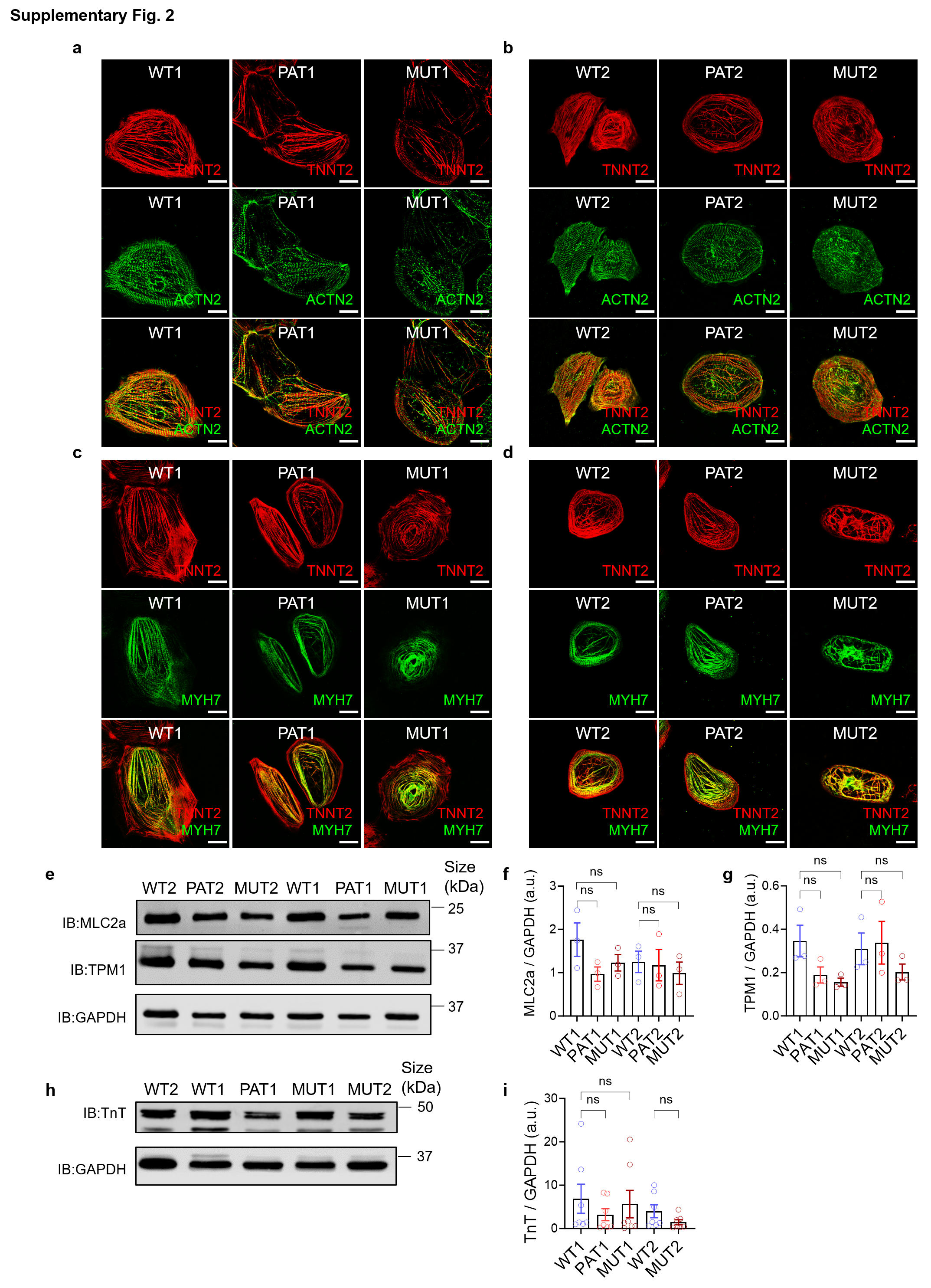
Figure. S2.

Characterization of DCM patient-specific and Cas9 mutation-introduced iPSC-CMs versus WT controls. DCM (PAT1, PAT2, MUT1, MUT2) iPSC-CMs and WT (WT1, WT2) iPSC-CMs show regular levels of cardiac markers. **a-b**, Representative confocal images following ACTN2- and TNNT2-specific immunostaining. Scale bar, 20 µm. **c-d**, Representative confocal images following MYH7- and TNNT2-specific immunostaining. Scale bar, 20 µm. **e**-**j**, iPSC-CMs display comparable protein levels of cardiac markers MLC2a, TPM1 and TnT. **e-g**, Representative membrane scans for MLC2a (**e**), TPM1 (**e**), and TnT (**h**) immunoblotting. **f-g**, Quantification of **e**. Data are normalized by GAPDH. Per group, n=1 experiment, n=3 technical replicates. ns, not significant (Kruskal-Wallis test, Dunn‘s post-hoc test). **i**, Quantification of **h**. Data are normalized by GAPDH. Per group, n=7 independent experiments. ns, not significant (Kruskal-Wallis test, Dunn‘s post-hoc test). Data are presented as the mean±SEM. A.u. indicates arbitrary units; PAT1, DCM patient-specific TPM1-L185F iPSC-CMs; PAT2, DCM patient-specific TnT-R173W iPSC-CMs; MUT1, iPSC-CMs containing CRISPR/Cas9-introduced TPM1-L185F-mutation; MUT2, iPSC-CMs containing CRISPR/Cas9-introduced TnT-R141W-mutation; WT, wild-type; TNNT2, cardiac troponin T; ACTN2, sarcomeric α-actinin; MYH7, myosin heavy chain 7; MLC2a, myosin light chain 2a; TPM1, tropomyosin 1; TnT, cardiac troponin T.


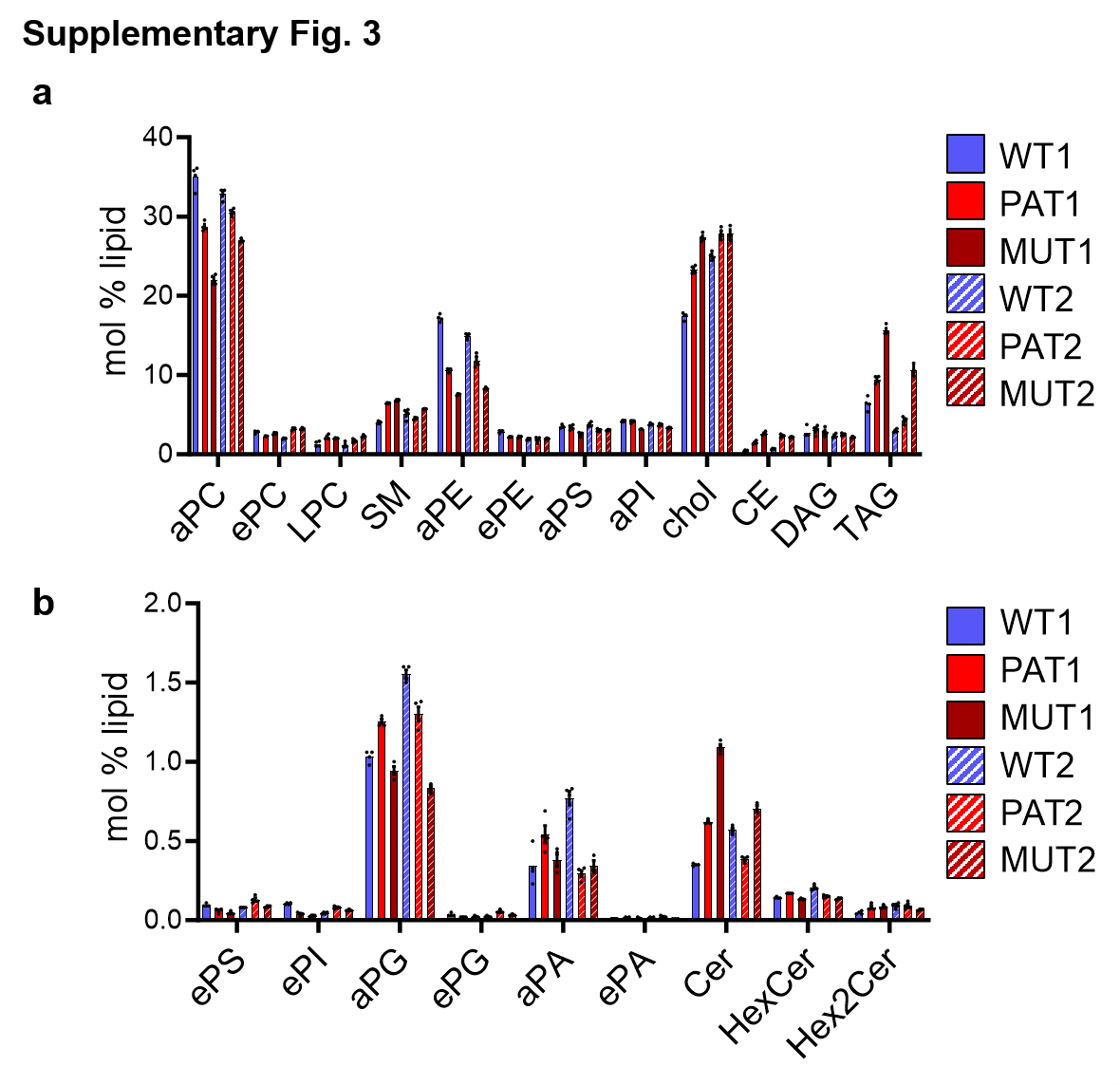


Figure. S3.

Lipid class profiles for DCM patient-specific and Cas9 mutation-introduced iPSCs versus WT controls (related to Fig. 1a). Lipid class profile for high (a) and low (b) abundant lipids via quantitative lipidomics analyses in DCM (PAT1, MUT1, PAT2, MUT2) iPSC-CMs compared with WT (WT1, WT2) iPSC-CMs. PAT1, DCM patient-specific TPM1-L185F iPSC-CMs; PAT2, DCM patient-specific TnT-R173W iPSC-CMs; MUT1, iPSC-CMs containing CRISPR/Cas9-introduced TPM1-L185F-mutation; MUT2, iPSC-CMs containing CRISPR/Cas9-introduced TnT-R141W-mutation; WT, wild-type; aPC, diacyl-phosphatidylcholine; ePC, acyl-phosphatidylcholine; LPC, lyso-phosphatidylcholine; SM, sphingomyelin; aPE, diacyl-phosphatidylethanolamine; ePE, acyl-phosphatidylethanolamine; aPS, diacyl-phosphatidylserine; aPI, diacyl-phosphatidylinositol; Chol, cholesterol; CE, cholesterol ester; DAG, diglyceride; TAG, triglyceride; ePS, acyl-phosphatidylserine; ePI, acyl-phosphatidylinositol; aPG, diacyl-phosphatidylglycerol; ePG, acyl-phosphatidylglycerol; aPA, diacyl-phosphatidate; ePA, diacyl-phosphatidate; Cer, ceramide; HexCer, hexosylceramide; Hex2Cer, dihexosylceramide.


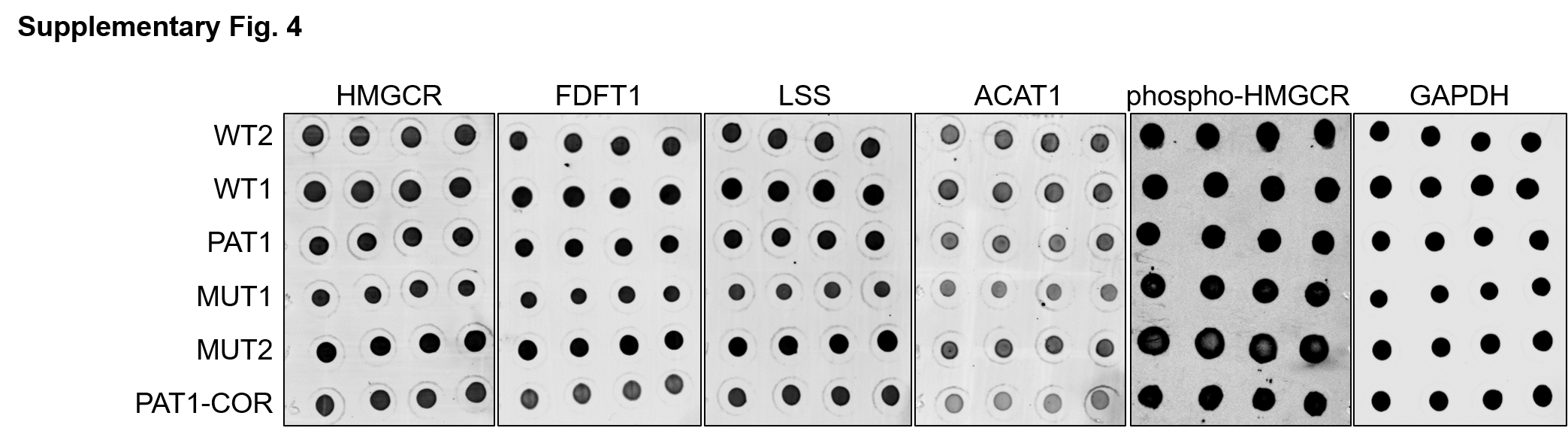
Figure. S4.

Immunoblot assessment of protein levels for key enzymes involved in cholesterol biosynthesis and turnover in DCM iPSC-CMs versus WT controls (related to Fig. 1j-n). Representative membrane scans for HMGCR, FDFT1, LSS, ACAT1, phospho-HMGCR, and GAPDH immunoblotting. PAT1, DCM patient-specific TPM1-L185F iPSC-CMs; MUT1, iPSC-CMs containing CRISPR/Cas9-introduced TPM1-L185F-mutation; MUT2, iPSC-CMs containing CRISPR/Cas9-introduced TnT-R141W-mutation; PAT1-COR, CRISPR/Cas9 TPM1-L185F mutation-corrected iPSC-CMs; WT, wild-type; HMGCR, 3-hydroxy-3-methylglutaryl-coenzyme A reductase; FDFT1, squalene synthase 1; LSS, lanosterol synthase; ACAT1, acyl-coenzyme A:cholesterol acyltransferase 1; phospho-HMGCR, 3-hydroxy-3-methylglutaryl-coenzyme A reductase phosphorylated at Ser872.


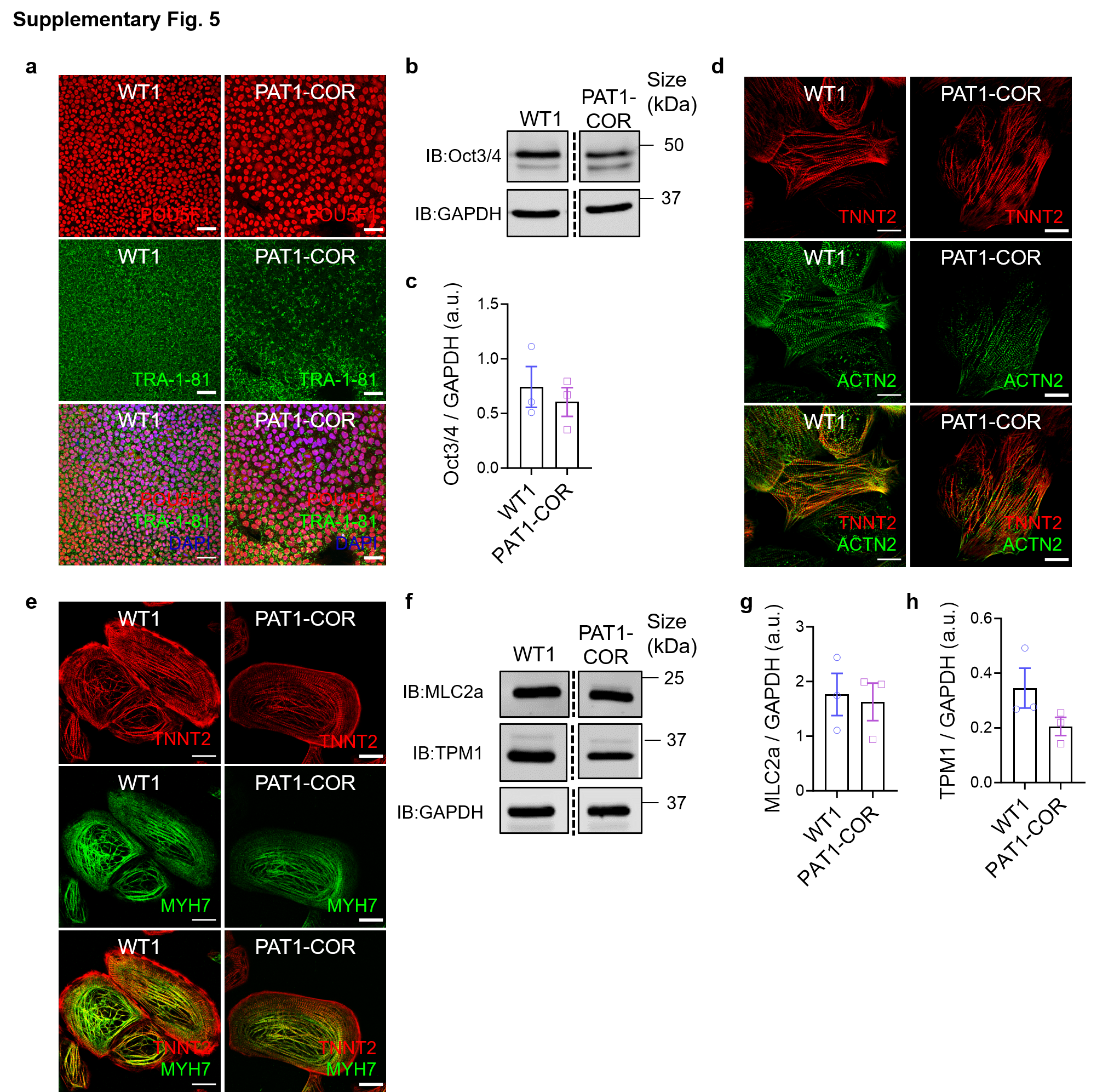
Figure. S5.

Characterization of CRISPR/Cas9 TPM1-L185F mutation-corrected iPSC and iPSC-CMs. **a-c**, CRISPR/Cas9 TPM1-L185F mutation-corrected (PAT1-COR) iPSCs show regular levels of pluripotency markers. **a**, Representative confocal images following POU5F1- and TRA-1-81-specific immunostaining together with DAPI staining. Scale bar, 50 µm. **b**-**c**, PAT1-COR and PAT1 iPSCs display comparable protein levels of pluripotency marker Oct3/4. **b**, Representative membrane scans for Oct3/4 immunoblotting. **c**, Quantification of **b**. Data are normalized by GAPDH. Per group, n=1 experiment, n=3 technical replicates. Data for PAT1 iPSC are the same as presented in supplementary Fig. 1d. **d-i**, CRISPR/Cas9 TPM1-L185F mutation-corrected (PAT1-COR) iPSC-CMs show regular levels of cardiac markers. **d**, Representative confocal images following ACTN2- and TNNT2-specific immunostaining. Scale bar, 20 µm. **e**, Representative confocal images following MYH7- and TNNT2-specific immunostaining. Scale bar, 20 µm. **f**-**h**, PAT1-COR and PAT1 iPSCs-CMs display comparable protein levels of cardiac markers MLC2a and TPM1. **f**, Representative membrane scans for MLC2a and TPM1 immunoblotting. **g-h**, Quantification of **f**. Data are normalized by GAPDH. Per group, n=1 experiment, n=3 technical replicates. Data for PAT1 iPSC-CMs are the same as presented in supplementary Fig. 2e-g. Data are presented as the mean±SEM. A.u. indicates arbitrary units; PAT1, DCM patient-specific TPM1-L185F iPSC and iPSC-CMs; PAT1-COR, CRISPR/Cas9 TPM1-L185F mutation-corrected iPSC and iPSC-CMs; POU5F1, POU class 5 homeobox 1; TRA-1-81, podocalyxin; TNNT2, cardiac troponin T; ACTN2, sarcomeric α-actinin; MYH7, myosin heavy chain 7; MLC2a, myosin light chain 2; TPM1, tropomyosin 1.


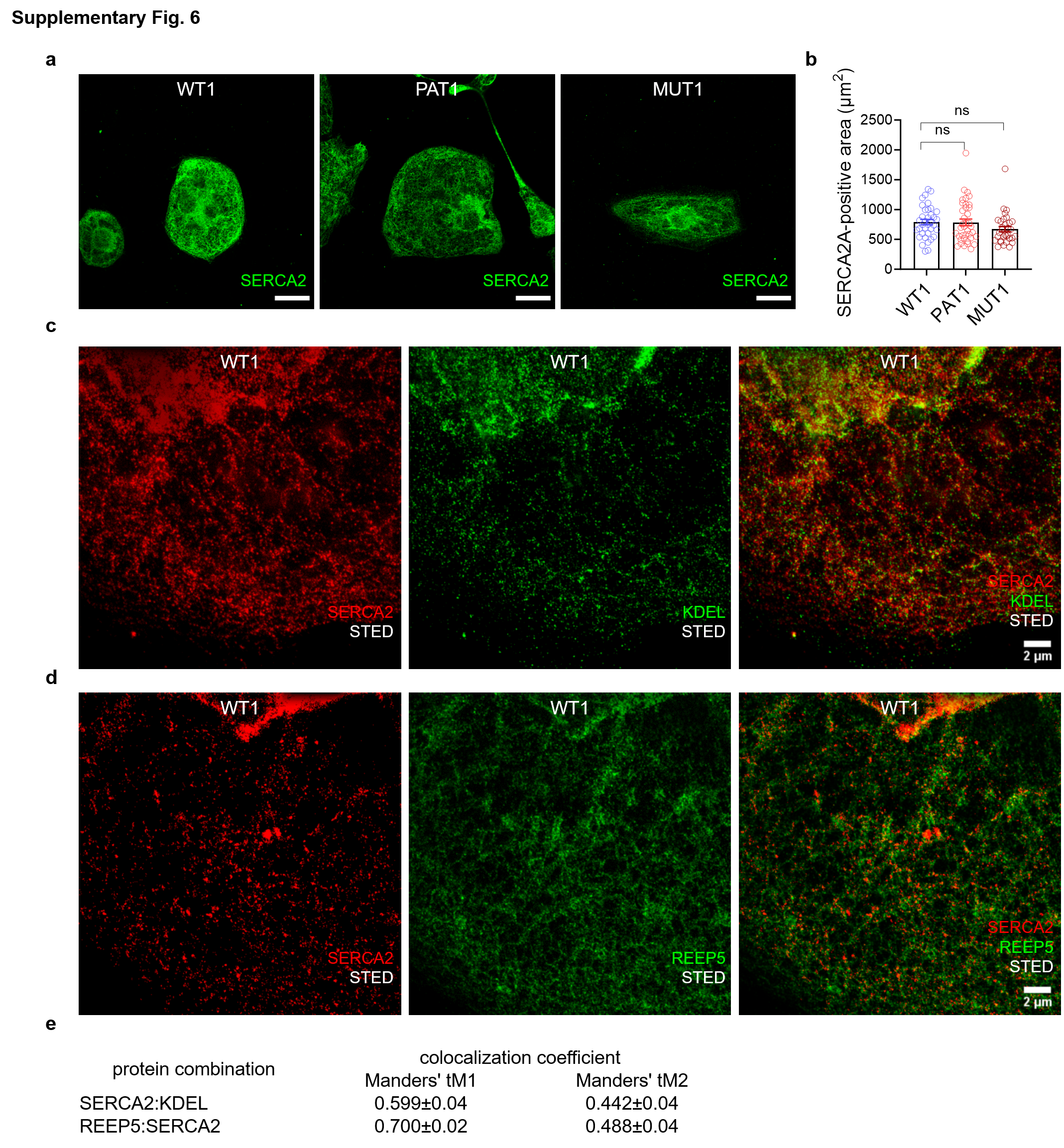
Figure. S6.

The intracellular area covered by the sarcoplasmic reticulum (SR) is not affected in DCM (MUT) iPSC-CMs. **a-b**, SR surface area is not significantly different in DCM (PAT1, MUT1) iPSC-CMs compared with WT (WT1) iPSC-CMs. **a**, Representative confocal images following SERCA2-specific immunostaining. Scale bar, 20 µm. **b**, Quantification of **a**. ns, not significant for PAT1 vs WT1 and MUT1 vs WT1 (Kruskal-Wallis test and Dunn's multiple comparisons test)*.* Per group, n=2 experiments; n=36 cells (WT1), 38 cells (PAT1), 32 cells (MUT1). **c**-**e**, Colocalization of SR and ER markers. **c**, Representative STED images following SERCA2- and KDEL-specific immunostaining. Scale bar, 2 µm. **d**, Representative STED images following SERCA2- and REEP5-specific immunostaining. Scale bar, 2 µm. **e**, Quantification of **c**-**d** via ImageJ plugin “Colocalization Threshold”. PAT1, DCM patient-specific TPM1-L185F iPSC-CMs; MUT1, iPSC-CMs containing CRISPR/Cas9-introduced TPM1-L185F-mutation; WT, wild-type; SERCA2A, sarcoplasmic/endoplasmic reticulum Ca^2+^‐ATPase 2a; REEP5, receptor expression-enhancing protein 5.


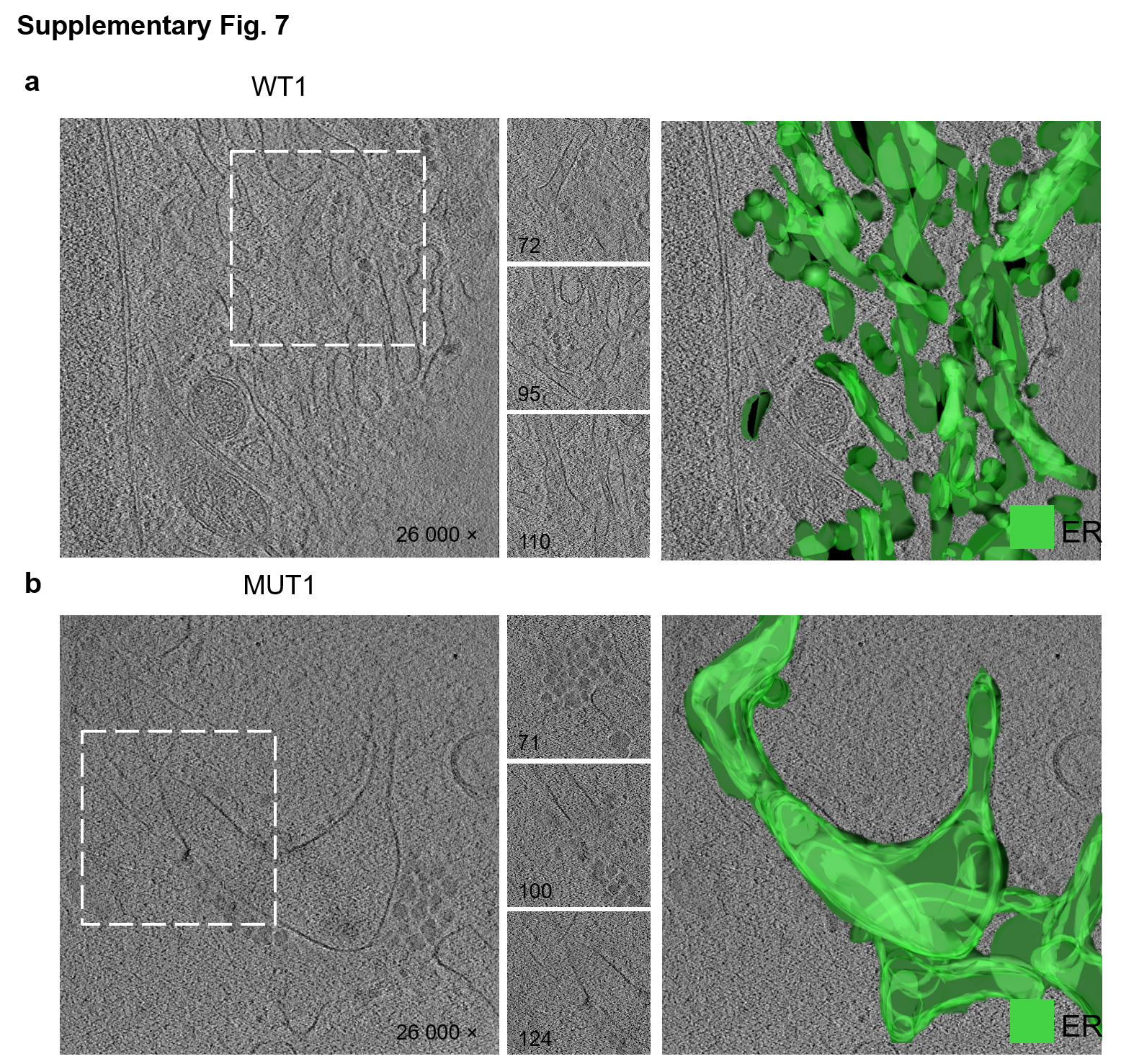


Figure. S7.

Analysis of ER membrane curvature in TPM1-L185F (MUT1) iPSC-CMs compared with isogenic control iPSC-CMs by cryo-electron tomography. Representative z-slices from reconstructed tomograms illustrating the ER in WT1 iPSC-CMs (**a**) and MUT1 iPSC-CMs (**b**). Enlarged panels show different z-slices through the same tomogram; slice numbers are indicated in the lower left corners. Segmentation of the ER membranes is overlaid to highlight ER structure. Per group: n = 1 experiment; n = 163 tomograms (WT1) and n = 51 tomograms (MUT1). MUT1, iPSC-CMs containing CRISPR/Cas9-introduced TPM1-L185F-mutation; WT, wild-type.


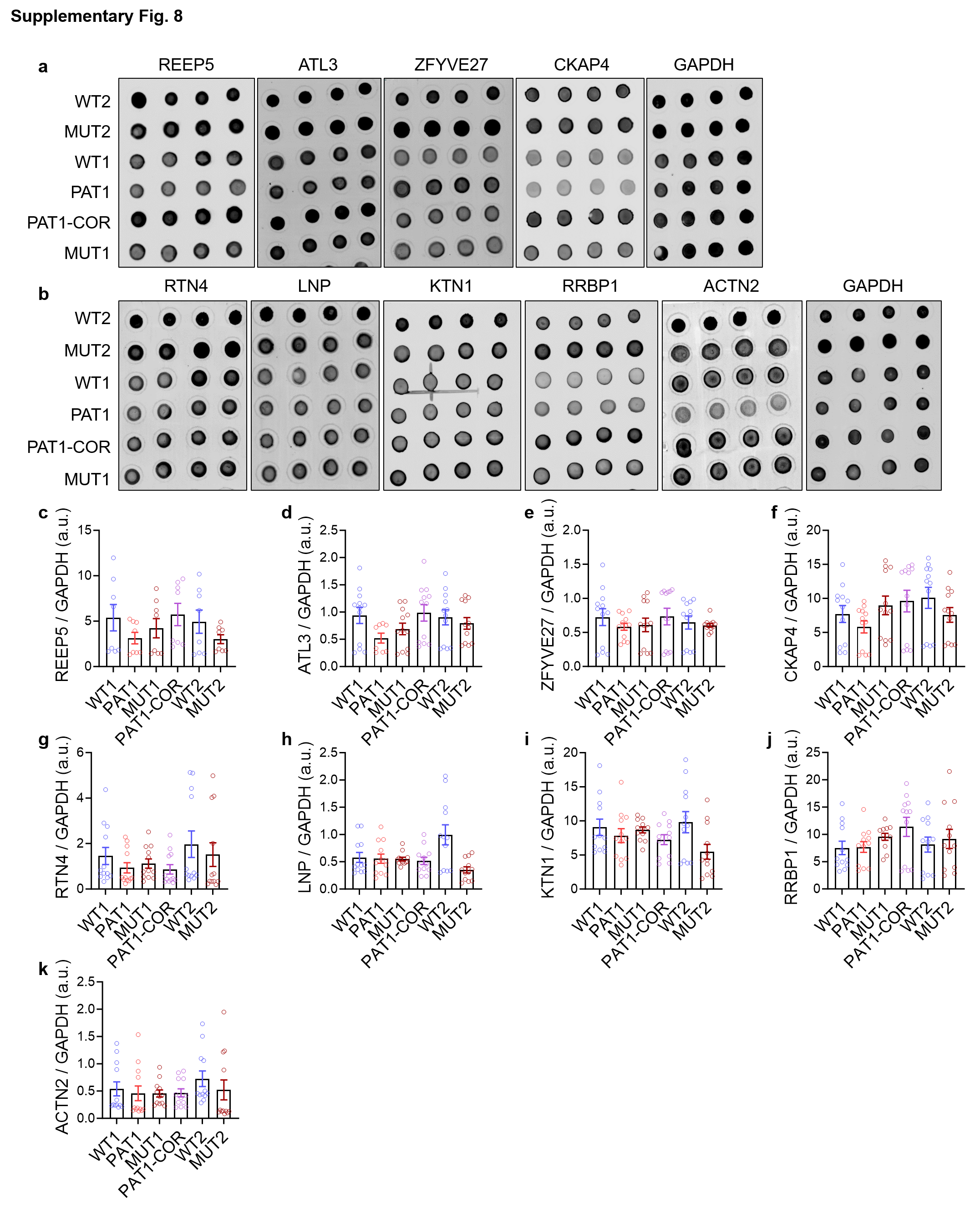
Figure. S8.

Immunoblot assessment of protein levels for ER structural and curvature-defining proteins in DCM (MUT) iPSC-CMs versus WT controls (related to Fig. 3h-p). **a**, Representative membrane scans for REEP5, ATL3, ZFYVE27, CKAP4, and GAPDH immunoblotting. **b**, Representative membrane scans for RTN4, LNP, KTN1, RRBP1, ACTN1, and GAPDH immunoblotting. **c-k**, Quantification of **a**-**b**. PAT1, DCM patient-specific TPM1-L185F iPSC-CMs; PAT2, DCM patient-specific TnT-R173W iPSC-CMs; MUT1, iPSC-CMs containing CRISPR/Cas9-introduced TPM1-L185F-mutation; MUT2, iPSC-CMs containing CRISPR/Cas9-introduced TnT-R141W-mutation; PAT1-COR, CRISPR/Cas9 TPM1-L185F mutation-corrected iPSC-CMs; WT, wild-type; REEP5, receptor expression-enhancing protein 5, RTN4, reticulon-4, ATL3, atlastin-3, LNP, ER junction formation protein lunapark, ZFYVE27, protrudin, KTN1, kinectin-1, RRBP1, ribosome-binding protein 1, CKAP4, cytoskeleton-associated protein 4, ACTN2, sarcomeric α-actinin.


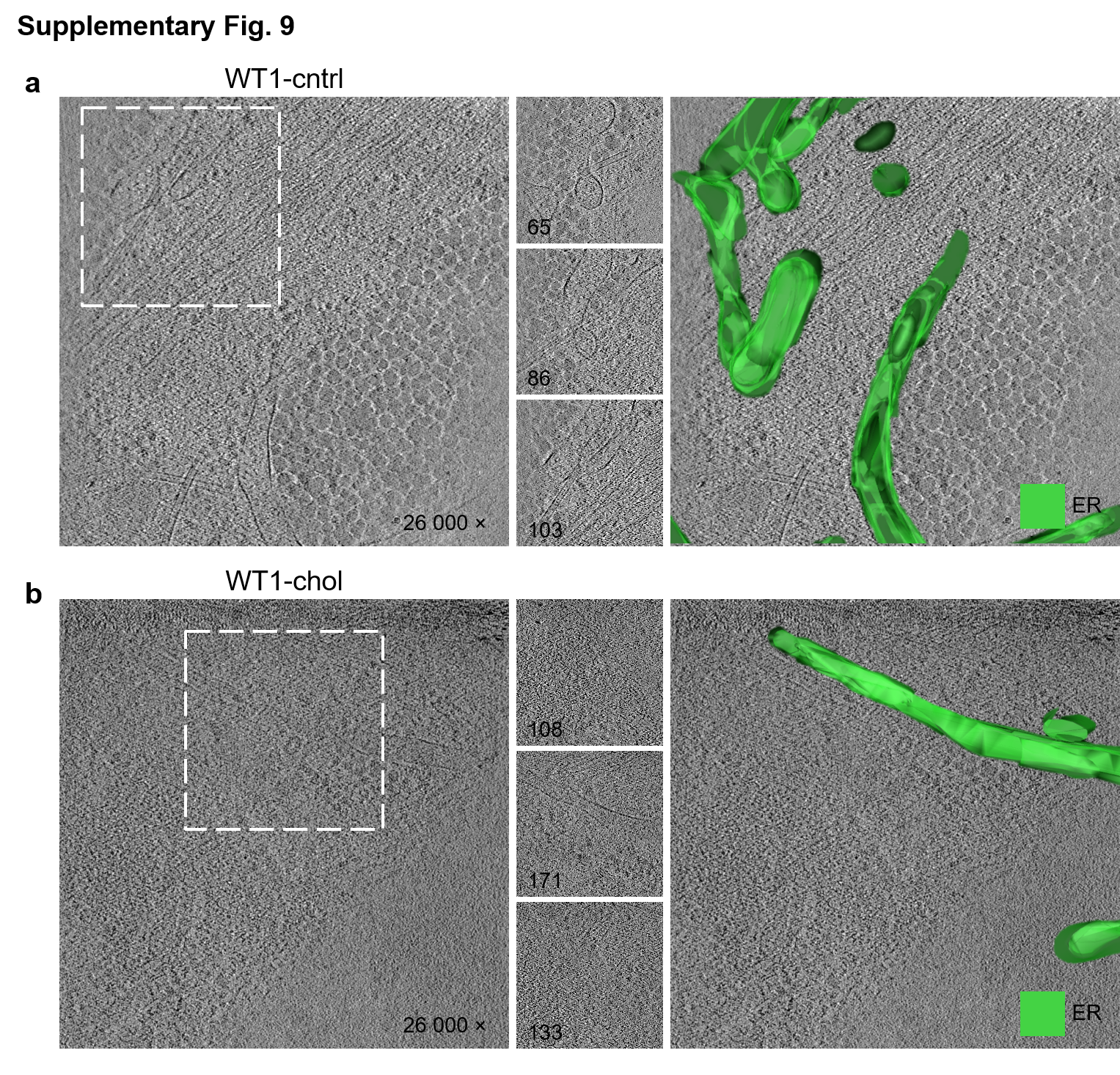


Figure. S9.

Analysis of ER membrane curvature in healthy control iPSC-CMs treated with 2 µM water soluble cholesterol (WT1-chol) compared with control vehicle (WT1-cntrl) by cryo-electron tomography. Representative z-slices from reconstructed tomograms illustrating the ER in WT1-cntrl iPSC-CMs (**a**) and WT1-chol iPSC-CMs (**b**). Enlarged panels show different z-slices through the same tomogram; slice numbers are indicated in the lower left corners. Segmentation of the ER membranes is overlaid to highlight ER structure. Per group: n = 1 experiment; n = 163 tomograms (WT1-cntrl) and n = 110 tomograms (WT1-chol). WT, wild-type; chol, cholesterol.


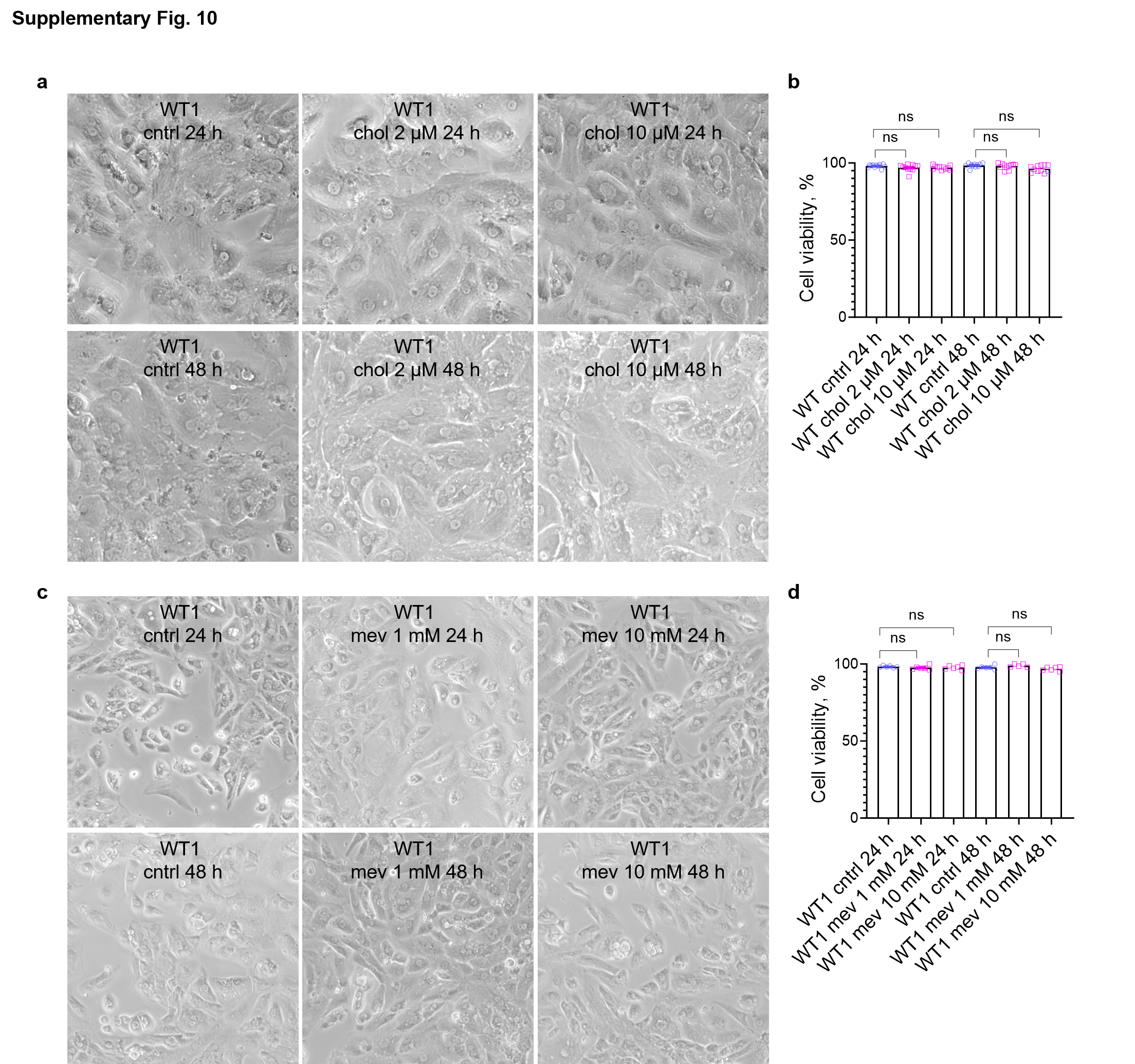
Figure. S10.

Cell viability is not altered following cholesterol or mevalonolactone treatment in iPSC-CMs, compared to control vehicle. **a**, Representative images for heathy control (WT1, WT2) iPSC-CMs treated with 2 μM or 10 μM water-soluble cholesterol (chol) or control vehicle (cntrl) at different time points (24 h or 48 h). **b**, Cell viability is not significantly altered in chol-treated WT iPSC-CMs vs control vehicle. WT1, n=1 experiment; n=3 technical replicates. WT2, n=2 experiments; n=4 technical replicates. ns, not significant for WT chol 2 μM 24 h vs WT cntrl 24 h, WT chol 10 μM 24 h vs WT cntrl 24 h, WT chol 2 μM 48 h vs WT cntrl 48 h, and WT chol 10 μM 48 h vs WT cntrl 48 h (Kruskal-Wallis test and Dunn's multiple comparisons test). **c**, Representative images for heathy control (WT1) iPSC-CMs treated with 1 mM or 10 mM mevalonolactone (mev) or control vehicle (cntrl) at different time points (24 h or 48 h). **d**, Cell viability is not significantly altered in mev-treated WT1 iPSC-CMs vs control vehicle. Per group, n=2 experiments; n=3 technical replicates. ns, not significant for WT1 mev 1 mM 24 h vs WT1 cntrl 24 h, WT1 mev 10 mM 24 h vs WT1 cntrl 24 h, WT1 mev 1 mM 48 h vs WT1 cntrl 48 h, and WT1 mev 10 mM 48 h vs WT1 cntrl 48 h (Kruskal-Wallis test and Dunn's multiple comparisons test). Data are presented as the mean±SEM. The number of independent experiments equals the number of independent cardiac differentiations. WT, wild-type; chol, cholesterol; mev, mevalonolactone.


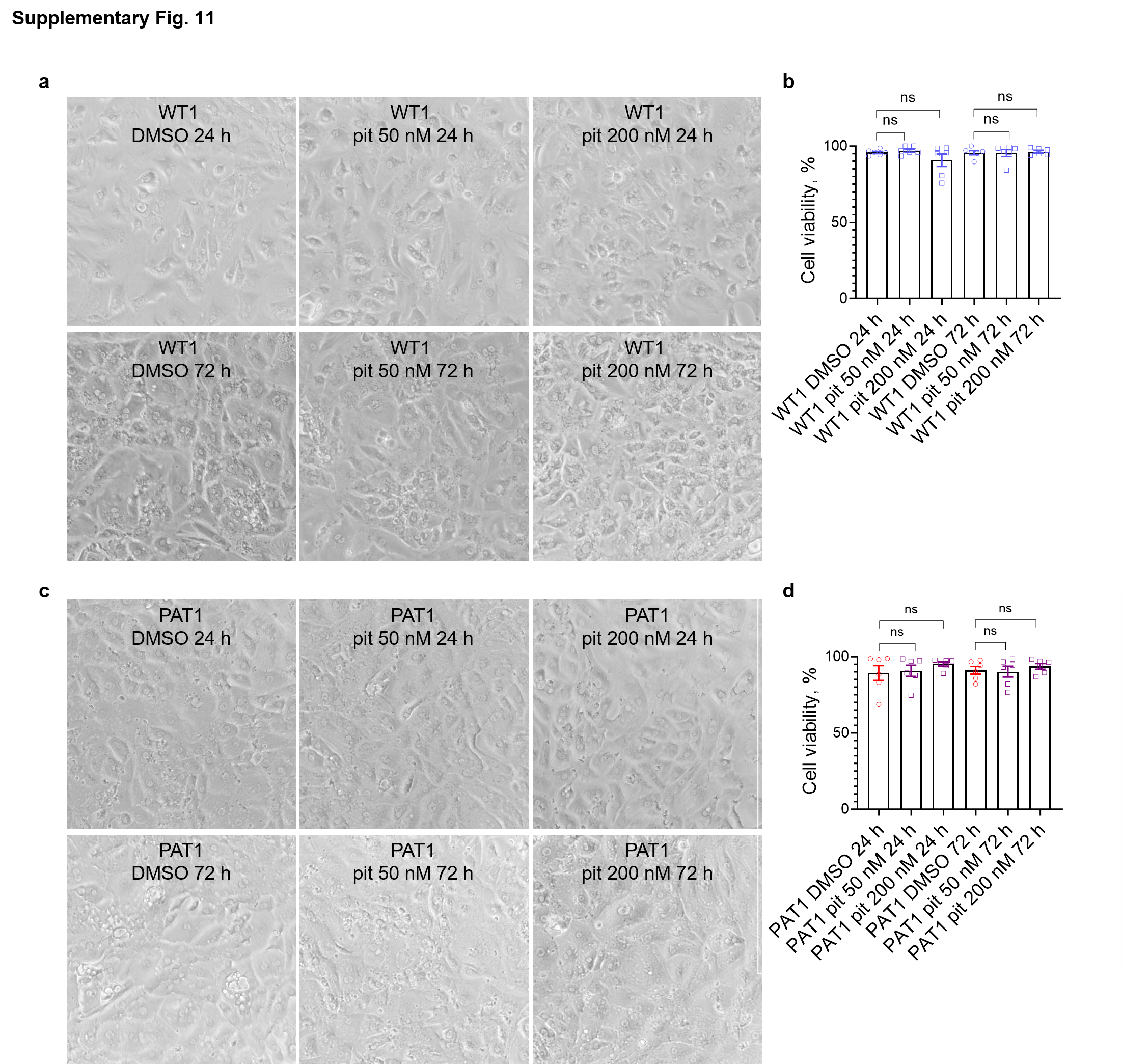
Figure. S11.

Cell viability is not altered following pitavastatin treatment in iPSC-CMs, compared to control vehicle. **a**, Representative images for heathy control (WT1) iPSC-CMs treated with 50 nM or 200 nM pitavastatin (pit) or control vehicle (DMSO) at different time points (24 h or 72 h). **b**, Cell viability is not significantly altered in pit-treated WT1 iPSC-CMs vs control vehicle. Per group, n=2 experiments; n=3 technical replicates. ns, not significant for WT1 pit 50 nM 24 h vs WT1 DMSO 24 h, WT1 pit 200 nM 24 h vs WT1 DMSO 24 h, WT1 pit 50 nM 72 h vs WT1 DMSO 72 h, and WT1 pit 200 nM 72 h vs WT1 DMSO 72 h (Kruskal-Wallis test and Dunn's multiple comparisons test). **c**, Representative images for DCM (PAT1) iPSC-CMs treated with 50 nM or 200 nM pitavastatin (pit) or control vehicle (DMSO) at different time points (24 h or 72 h). **d**, Cell viability is not significantly altered in pit-treated PAT1 iPSC-CMs vs control vehicle. Per group, n=2 experiments; n=3 technical replicates. ns, not significant for PAT1 pit 50 nM 24 h vs PAT1 DMSO 24 h, PAT1 pit 200 nM 24 h vs PAT1 DMSO 24 h, PAT1 pit 50 nM 72 h vs PAT1 DMSO 72 h, and PAT1 pit 200 nM 72 h vs PAT1 DMSO 72 h (Kruskal-Wallis test and Dunn's multiple comparisons test). Data are presented as the mean±SEM. The number of independent experiments equals the number of independent cardiac differentiations. PAT1, DCM patient-specific TPM1-L185F iPSC-CMs; WT, wild-type; pit, pitavastatin.


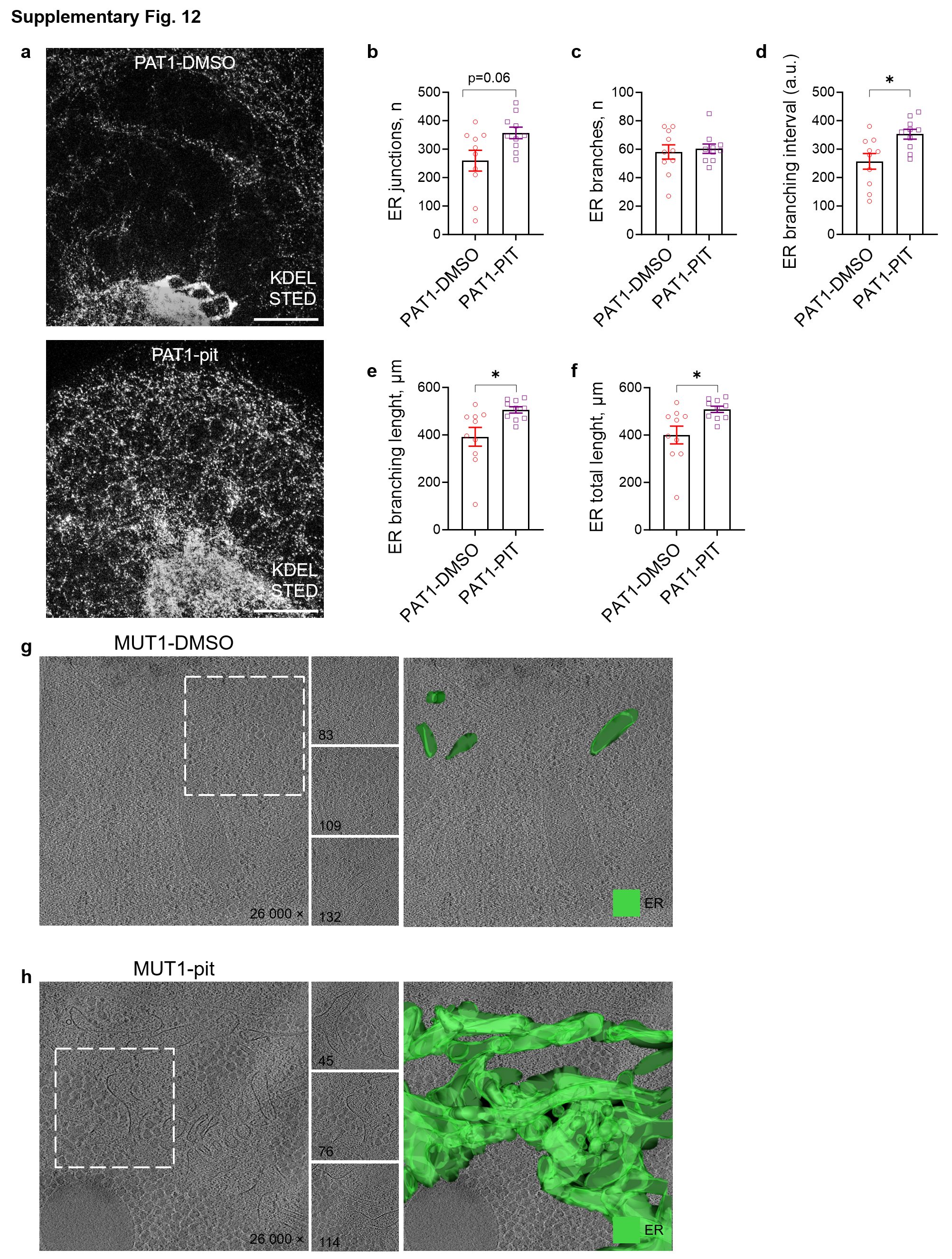
Figure. S12.

Analysis of ER membrane curvature in pitavastatin-treated DCM (MUT) iPSC-CMs compared with control vehicle iPSC-CMs at the nanoscale level using STED and cryo-electron tomography. **a**, STED for KDEL immunostaining reveals that patho-phenotypic ER architecture is rescued in pit-treated DCM patient-derived iPSC-CMs (PAT1) compared with control vehicle iPSC-CMs. Representative magnified STED images (zoom factor=5×) are shown. Scale bar, 5 µm. **b-f**, Quantitative analyses of ER organization using images shown in **a**. **b**, Number of ER junctions following pit treatment (Mann-Whitney test). **c**, Number of ER branches following pit treatment (Mann-Whitney test). **d**, Increased ER branching interval following pit treatment. *P<0.05 for PAT1-pit vs PAT1-DMSO (Mann-Whitney test). **e**, Increased ER branching length following pitavastatin treatment. *P<0.05 for PAT1-pit vs PAT1-DMSO (Mann-Whitney test). **f**, Increased ER total length following pitavastatin treatment. *P<0.05 for PAT1-pit vs PAT1-DMSO (Mann-Whitney test). Data are presented as the mean±SEM. Per group, n=10 images (PAT1-DMSO), 10 images (PAT1-pit). One field of view per cell was imaged. **g-h**, Representative z-slices from reconstructed tomograms illustrating the ER in MUT1-DMSO iPSC-CMs (**g**) and MUT1-pit iPSC-CMs (**h**). Enlarged panels show different z-slices through the same tomogram; slice numbers are indicated in the lower left corners. Segmentation of the ER membranes is overlaid to highlight ER structure. Per group: n = 1 experiment; n = 23 tomograms (MUT1-DMSO) and n = 34 tomograms (MUT1-pit). A.u. indicates arbitrary units; PAT1, DCM patient-specific TPM1-L185F iPSC-CMs; MUT1, iPSC-CMs containing CRISPR/Cas9-introduced TPM1-L185F-mutation; pit, pitavastatin.


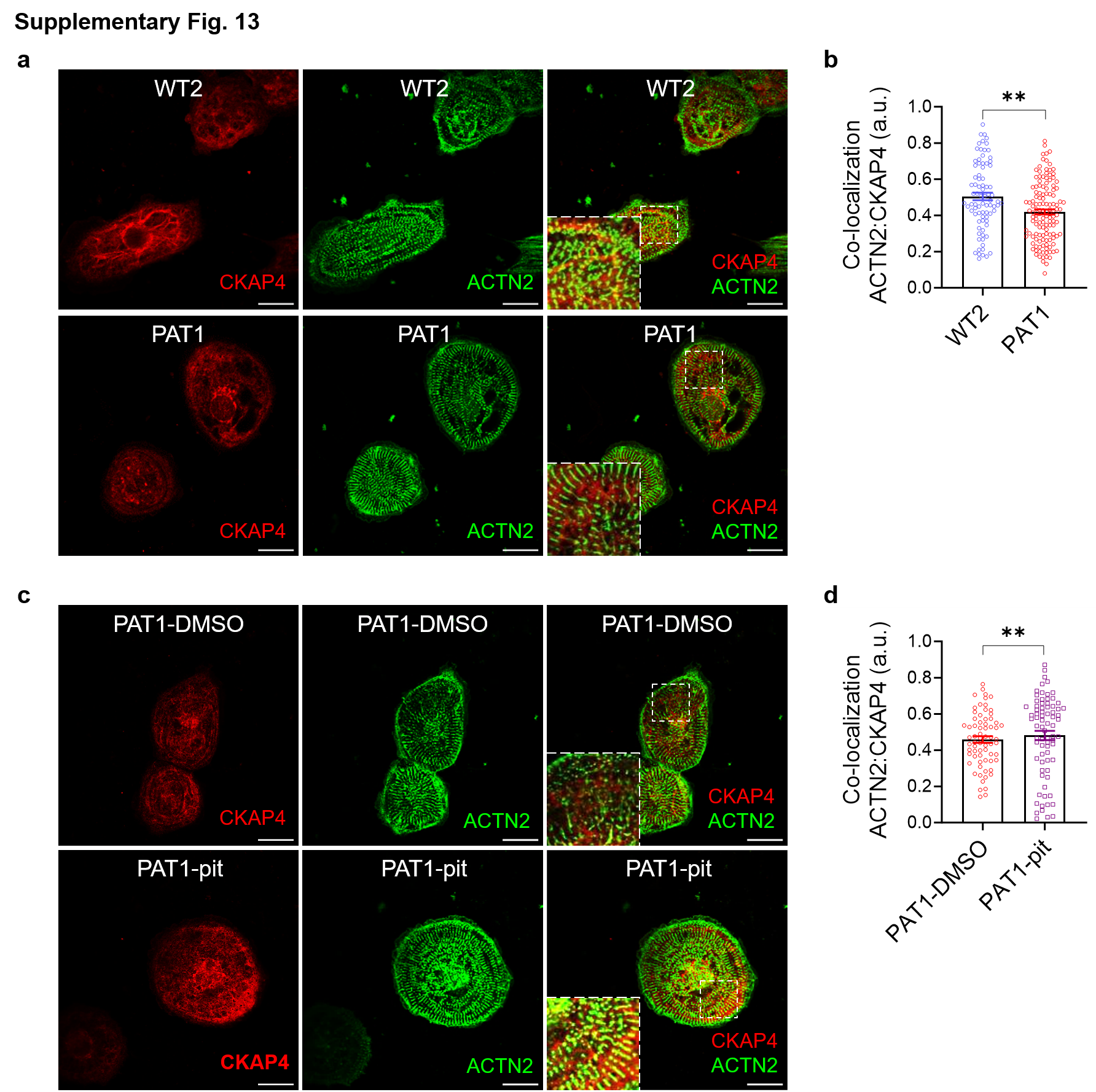


Figure. S13.

CKAP4-dependent ER-interactions with the sarcomere cytoskeleton are reduced in DCM (MUT) iPSC-CMs and rescued following pitavastatin treatment. **a**, Representative confocal images for CKAP4 and ACTN2 immunostaining in patient-specific iPSC-CMs with mutation TPM1-L185F (PAT1) compared with healthy control iPSC-CMs (WT2). Scale bar, 20 µm. **b**, Colocalization (Manders') analysis of CKAP4 overlap with ACTN2 for images shown in (**a**). **P<0.01 (Mann-Whitney test). WT2: n=5 experiments; n=82 images; PAT1: n=6 experiments, 131 images. **c**, Representative confocal images for CKAP4 and ACTN2 immunostaining in pit-treated patient-specific iPSC-CMs (PAT1-pit) and control vehicle (PAT1-DMSO). Scale bar, 20 µm. **d**, Colocalization (Manders') analysis of CKAP4 overlap with ACTN2 for images shown in (**c**). **P<0.01 (Mann-Whitney test). Per group, n=4 experiments; n=68 images (PAT1-cntrl), n=76 images (PAT1-pit). A.u. indicates arbitrary units; PAT1, DCM patient-specific TPM1-L185F iPSC-CMs; WT, wild-type; pit, pitavastatin; CKAP4, cytoskeleton-associated protein 4, ACTN2, sarcomeric α-actinin.


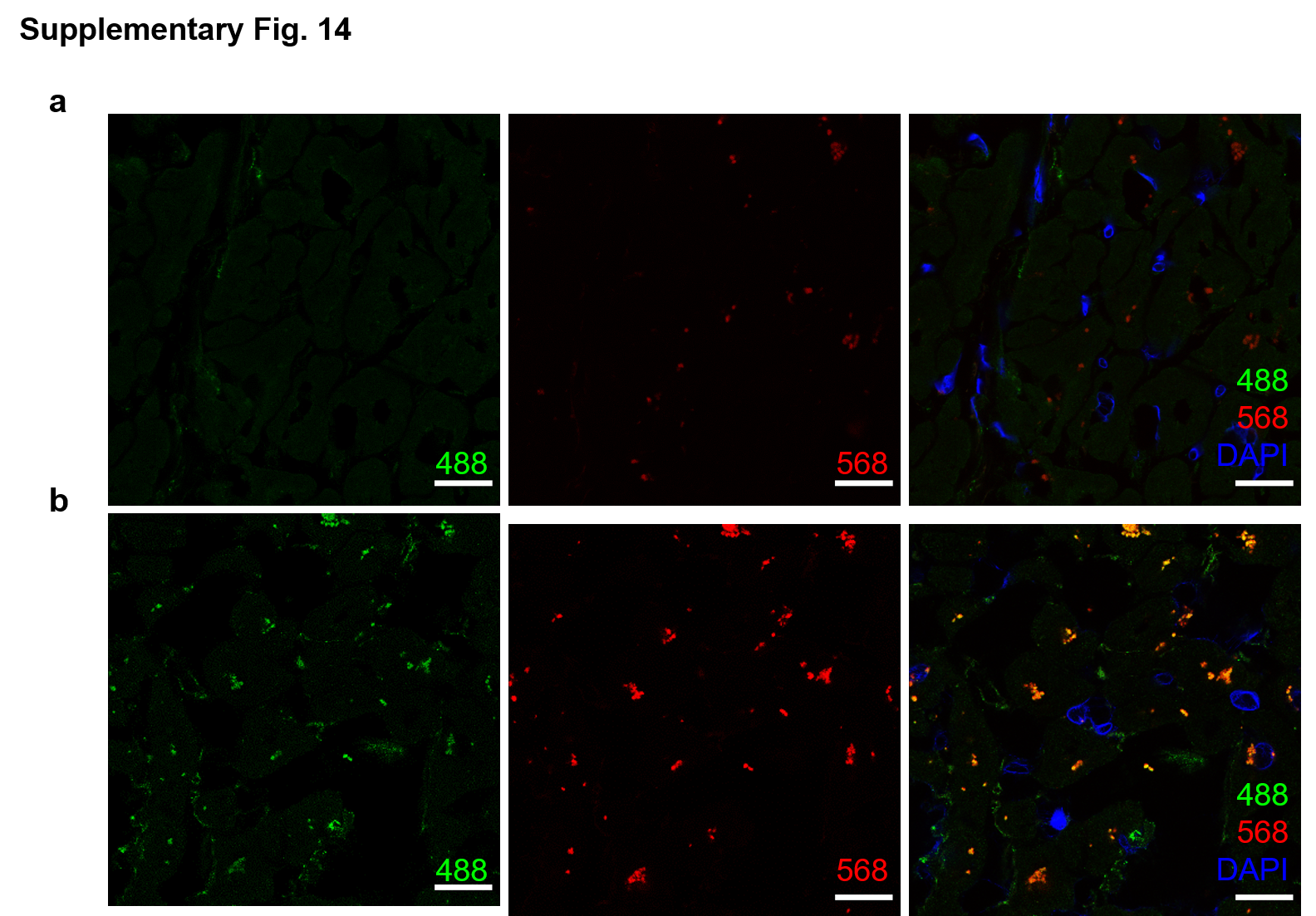


Figure. S14.

Negative controls for immune-detection of proteins in human heart tissue staining (related to Fig. 7g-h). Negative controls are shown for immunostaining for the ER marker Sec61β (green). **a**, Representative confocal images for left ventricle (LV) tissue from a control donor following immunostaining with an unspecific rabbit IgG antibody and secondary Alexa Fluor 488 goat anti-rabbit antibody together with DAPI (blue). Scale bar, 20 µm. **b**, Representative confocal images for LV tissue from a control donor following immunostaining with a Sec61β rabbit antibody and secondary Alexa Fluor 488 goat anti-mouse antibody together with DAPI (blue). Scale bar, 20 µm. Per group, n=1 experiment.


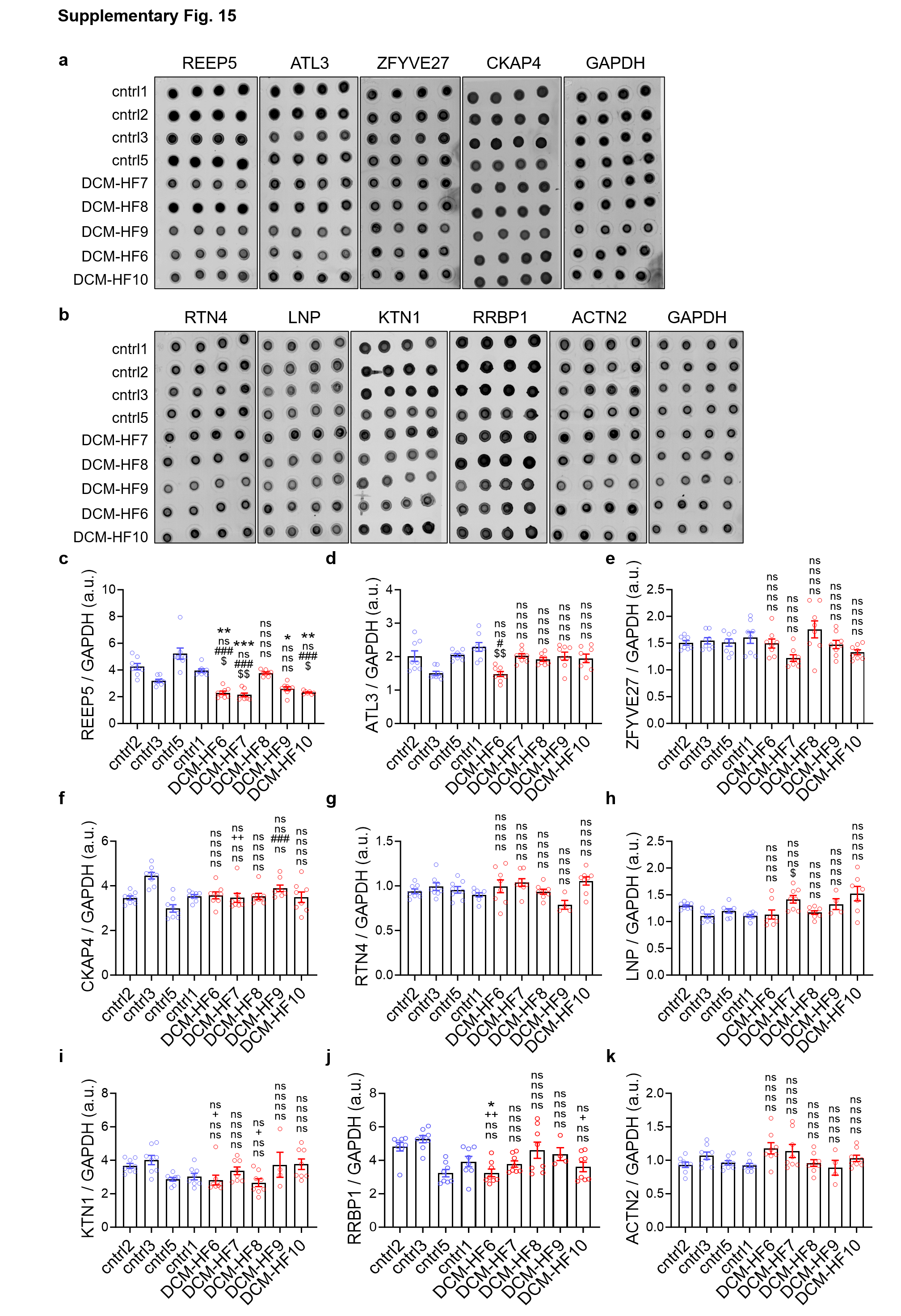
Figure. S15.

Immunoblot assessment of protein levels for ER structural and curvature-defining proteins in adult heart tissues from DCM patients with end-stage heart failure versus donor controls (related to Fig. 8a-i). a, Representative membrane scans for REEP5, ATL3, ZFYVE27, CKAP4, and GAPDH immunoblotting. **b**, Representative membrane scans for RTN4, LNP, KTN1, RRBP1, ACTN1, and GAPDH immunoblotting. **c-k**, Quantification of **a**-**b**. Patient HF8 received 40 mg simvastatin once a day in the evening. ***P<0.001 for HF7 vs cntrl2 (REEP5), **P<0.01 for HF6 and HF10 vs cntr2 (REEP5), *P<0.05 for HF9 vs cntrl2 (REEP5), HF6 vs cntrl2 (RRBP1). ++P<0.01 for HF7 vs cntrl3 (CKAP4), HF6 vs cntrl3 (RRBP1), +P<0.05 for HF6 and HF8 vs cntrl3 (KTN1), HF10 vs cntrl3 (RRBP1). ###P<0.001 for HF6, HF7 and HF10 vs cntrl5 (REEP5), HF9 vs cntrl5 (CKAP4), #P<0.05 for HF6 vs cntrl5 (ATL3). $$P<0.01 for HF7 vs cntrl1 (REEP5), HF6 vs cntrl1 (ATL3), $P<0.05 for HF6 and HF10 vs cntrl1 (REEP5). ns, not significant by an ordinary one-way ANOVA and Tukey's multiple comparison test (ZFYVE27, RTN4, ACTN2) or by Kruskal-Wallis test and by Dunn's multiple comparisons test (REEP5, ATL3, CKAP4, LNP, KTN1, RRBP1). Per group, n=2 independent experiments, n=4 technical replicates, except for HF9, which has n=1 experiment for proteins RTN4, LNP, KTN1, RRBP1, ACTN2. Data are presented as the mean±SEM. REEP5, receptor expression-enhancing protein 5, ATL3, atlastin-3, ZFYVE27, protrudin, CKAP4, cytoskeleton-associated protein 4, RTN4, reticulon-4, LNP, ER junction formation protein lunapark, KTN1, kinectin-1, RRBP1, ribosome-binding protein 1, ACTN2, sarcomeric α-actinin.

| iPSC-CM line | Mean Diff. | | Summary | | Adjusted *p* value | |
| --- | --- | --- | --- | --- | --- | --- |
|  | aPC | | | | | |
| WT1 vs. PAT1 | 6.283 | | *** | | <0.0001 | |
| WT1 vs. MUT1 | 13 | | *** | | <0.0001 | |
| WT2 vs. PAT2 | 2.338 | | ** | | 0.0029 | |
| WT2 vs. MUT2 | 5.938 | | *** | | <0.0001 | |
|  | ePC | | | | | |
| WT1 vs. PAT1 | 0.5275 | | *** | | <0.0001 | |
| WT1 vs. MUT1 | 0.1805 | | ns | | 0.1758 | |
| WT2 vs. PAT2 | -1.198 | | *** | | <0.0001 | |
| WT2 vs. MUT2 | -1.245 | | *** | | <0.0001 | |
|  | LPC | | | | | |
| WT1 vs. PAT1 | -0.86 | | *** | | 0.0007 | |
| WT1 vs. MUT1 | -0.6986 | | ** | | 0.0047 | |
| WT2 vs. PAT2 | -0.5475 | | * | | 0.0286 | |
| WT2 vs. MUT2 | -1.108 | | *** | | 0.0001 | |
|  | SM | | | | | |
| WT1 vs. PAT1 | -2.438 | | *** | | <0.0001 | |
| WT1 vs. MUT1 | -2.8 | | *** | | <0.0001 | |
| WT2 vs. PAT2 | 0.6175 | | * | | 0.0365 | |
| WT2 vs. MUT2 | -0.5908 | | ns | | 0.0726 | |
|  | aPE | | | | | |
| WT1 vs. PAT1 | 6.56 | | *** | | <0.0001 | |
| WT1 vs. MUT1 | 9.647 | | *** | | <0.0001 | |
| WT2 vs. PAT2 | 3.105 | | *** | | <0.0001 | |
| WT2 vs. MUT2 | 6.658 | | *** | | <0.0001 | |
|  | ePE | | | | | |
| WT1 vs. PAT1 | 0.68 | | *** | | 0.0001 | |
| WT1 vs. MUT1 | 0.6626 | | *** | | 0.0002 | |
| WT2 vs. PAT2 | 0.03 | | ns | | 0.9986 | |
| WT2 vs. MUT2 | -0.04917 | | ns | | 0.9931 | |
|  | aPS | | | | |  |
| WT1 vs. PAT1 | 0.1 | ns | | 0.9622 | |  |
| WT1 vs. MUT1 | 0.9332 | *** | | 0.0001 | |  |
| WT2 vs. PAT2 | 0.7275 | ** | | 0.0018 | |  |
| WT2 vs. MUT2 | 0.6867 | ** | | 0.0059 | |  |
|  | aPI | | | | |  |
| WT1 vs. PAT1 | 0.0575 | ns | | 0.9344 | |  |
| WT1 vs. MUT1 | 1.03 | **** | | <0.0001 | |  |
| WT2 vs. PAT2 | 0.0725 | ns | | 0.8619 | |  |
| WT2 vs. MUT2 | 0.4408 | *** | | 0.0005 | |  |
|  | chol | | | | |  |
| WT1 vs. PAT1 | -5.873 | *** | | <0.0001 | |  |
| WT1 vs. MUT1 | -9.908 | *** | | <0.0001 | |  |
| WT2 vs. PAT2 | -2.825 | *** | | <0.0001 | |  |
| WT2 vs. MUT2 | -2.777 | *** | | <0.0001 | |  |
|  | CE | | | | |  |
| WT1 vs. PAT1 | -1.053 | *** | | <0.0001 | |  |
| WT1 vs. MUT1 | -2.195 | *** | | <0.0001 | |  |
| WT2 vs. PAT2 | -1.663 | *** | | <0.0001 | |  |
| WT2 vs. MUT2 | -1.508 | *** | | <0.0001 | |  |
|  | DAG | | | | |  |
| WT1 vs. PAT1 | -0.3225 | ns | | 0.7812 | |  |
| WT1 vs. MUT1 | -0.04214 | ns | | 0.9999 | |  |
| WT2 vs. PAT2 | -0.1575 | ns | | 0.9792 | |  |
| WT2 vs. MUT2 | 0.1792 | ns | | 0.9749 | |  |
|  | TAG | | | | |  |
| WT1 vs. PAT1 | -3.023 | *** | | <0.0001 | |  |
| WT1 vs. MUT1 | -9.225 | *** | | <0.0001 | |  |
| WT2 vs. PAT2 | -1.353 | * | | 0.0178 | |  |
| WT2 vs. MUT2 | -7.704 | *** | | <0.0001 | |  |

Table S1.

Statistical evaluation of lipidomics analyses for highly abundant lipids using one-way ANOVA and Šídák's post-hoc test (related to Fig. 1b and supplementary Fig. 3a). PAT1, DCM patient-specific TPM1-L185F iPSC-CMs; PAT2, DCM patient-specific TnT-R173W iPSC-CMs; MUT1, iPSC-CMs containing CRISPR/Cas9-introduced TPM1-L185F-mutation; MUT2, iPSC-CMs containing CRISPR/Cas9-introduced TnT-R141W-mutation; WT, wild-type; aPC, diacyl-phosphatidylcholine; ePC, acyl-phosphatidylcholine; LPC, lyso-phosphatidylcholine; SM, sphingomyelin; aPE, diacyl-phosphatidylethanolamine; ePE, acyl-phosphatidylethanolamine; aPS, diacyl-phosphatidylserine; aPI, diacyl-phosphatidylinositol; Chol, cholesterol; CE, cholesterol ester; DAG, diglyceride; TAG, triglyceride; ns, not significant.

| **Patient ID** | | **Patients conditions** |
| --- | --- | --- |
| DCM-HF1 | end-stage HF due to DCM | |
| DCM-HF2 |  |  |
| DCM-HF3 |  |  |
| DCM-HF4 |  |  |
| DCM-HF5 |  |  |
| DCM-HF6 |  |  |
| DCM-HF7 |  |  |
| DCM-HF8 |  |  |
| DCM-HF9 |  |  |
| DCM-HF10 |  |  |
| DCM-HF11 |  |  |
| Cntrl1 | | healthy donors without heart disease |
| Cntrl2 | |  |
| Cntrl3 | |  |
| Cntrl4 | |  |
| Cntrl5 | |  |

Table S2.

Information on adult human left ventricular tissue samples from DCM patients who received heart transplantation and from healthy donors corresponding to Fig. 7, Fig. 8a-l and supplementary Fig. 15. Patient DCM-HF8 received simvastatin.

|  | HF patients with DCM (IF) | HF patients with DCM (Immunoblotting) | HF patients with DCM (Lipidomics) |
| --- | --- | --- | --- |
| Count (n) | 5 | 5 | 4 |
| Clinical characteristics | | | |
| Indication for open-heart surgery | Heart transplantation | Heart transplantation | Heart transplantation |
| Age (years) | 35 ± 16.9 | 57.0 ± 7.9 | 41.8 ± 13.3 |
| Sex, female, n (%) | 5 (83.3) | 2 (40) | 2 (50) |
| Body mass index (kg/m²) | 24.2 ± 7 | 26.5 ± 4.5 | 27.3 ± 7 |
| LV ejection fraction (%) | 17.6 ± 8.3 | 20.6 ± 3.7 | 23.3 ± 4.7 |
| LV assist device, n (%) | 4 (66.7) | 3 (60) | 4 (100) |
| ICD, n (%) | 1 (16.7) | 5 (100) | 2 (50) |
| Coronary artery disease, n (%) | 0 (0) | 0 (0) | 0 (0) |
| Prior myocardial infarction, n (%) | 0 (0) | 0 (0) | 0 (0) |
| Prior PCI, n (%) | 0 (0) | 0 (0) | 0 (0) |
| Prior CABG, n (%) | 0 (0) | 0 (0) | 0 (0) |
| Hypertension, n (%) | 0 (0) | 1 (20) | 0 (0) |
| Hyperlipidemia, n (%) | 1 (16.7) | 3 (60) | 0 (0) |
| Diabetes, n (%) | 0 (0) | 1 (20) | 0 (0) |
| Atrial fibrillation, n (%) | 2 (33.3) | 3 (60) | 2 (50) |
| Peripheral vascular disease, n (%) | 0 (0) | 0 (0) | 0 (0) |
| Prior cerebral ischemia event, n (%) | 0 (0) | 1 (20) | 0 (0) |
| Chronic pulmonary disease, n (%) | 0 (0) | 0 (0) | 0 (0) |
| Medication | | | |
| ACE inhibitors | 3 (50) | 2 (40) | 4 (100) |
| AT1 blockers | 2 (33.3) | 1 (20) | 0 (0) |
| Beta-blockers | 4 (66.7) | 3 (60) | 4 (100) |
| Calcium channel inhibitors | 0 (0) | 0 (0) | 0 (0) |
| Digitalis | 0 (0) | 1 (20) | 0 (0) |
| Lipid-lowering drugs | 0 (0) | 1 (20) | 0 (0) |
| Mineralocorticoid-receptor antagonists | 3 (50) | 2 (40) | 3 (75) |
| SGLT2-Inhibitor | 1 (16.7) | 0 (0) | 0 (0) |
| Vericiguat | 1 (16.7) | 0 (0) | 0 (0) |
| Other diuretics | 3 (50) | 4 (80) | 3 (75) |

Table S3.

Characteristics and clinical history of heart failure (DCM-HF) patients and control donors corresponding to Fig. 7, Fig. 8a-l and supplementary Fig. 15. Patient DCM-HF8 received simvastatin.

| Category | Parameter | Patient 1 | Patient 2 | Patient 3 |
| --- | --- | --- | --- | --- |
| General | Sex | male | male | male |
|  | Age, y | 64 | 62 | 62 |
|  | Body mass index, kg/m² | 29.3524 | 28.0899 | 26.1224 |
| Intervention | CAD | + | + | + |
|  | MVD/AVD | - | - | - |
|  | CAD+MVD/AVD | - | - | - |
| Anamnesis | Hypertension | + | + | + |
|  | Diabetes | - | - | - |
|  | Hyperlipidemia | - | + | - |
|  | NYHA (I/II/III) | II | I | - |
|  | Stroke | - | - | - |
|  | TIA | - | - | - |
| Clinical chemistry | Creatinin, µmol/L | 0.99 | 1.07 | 0.75 |
| Echocardiography | LAD, mm | 40 | 34 | 50 |
|  | LVEDD, mm | 40 | 45 | 45 |
|  | LVEF, % | 50 | 44 | 50 |
| Medication | Digitalis | - | - | - |
|  | ACE inhibitors | - | - | + |
|  | AT1 blockers | + | + | - |
|  | β-Blockers | + | + | - |
|  | Diuretics | - | + | - |
|  | Nitrates | - | - | - |
|  | Lipid-lowering drugs | + | + | + |

Table S4.

Clinical characteristics of patients used for human cardiomyocyte isolation corresponding to Fig. 8m-n. ACE, angiotensin-converting enzyme; AT, angiotensin receptor; CAD, coronary artery disease; LAD, left atrial diameter; LVEDD, left ventricular end-diastolic diameter; LVEF, left ventricular ejection fraction; NYHA, New York Heart Association Functional Classification; MVD/AVD, mitral/aortic valve disease; TIA, transient ischemic attack. Continuous and categorical data are presented as actual values. “+” and “-” signifies presence or absence of selected parameters, respectively.

Movie S1.

Reconstructed tomogram for WT iPSC-CM (corresponding to Fig. 3c).

Movie S2.

ER segmentation for WT iPSC-CM (corresponding to Fig. 3c).

Movie S3.

Reconstructed tomogram for DCM (MUT) iPSC-CM (corresponding to Fig. 3d).

Movie S4.

ER segmentation for DCM (MUT) iPSC-CM (corresponding to Fig. 3d).

Movie S5.

Reconstructed tomogram for WT1 iPSC-CM (corresponding to supplementary Fig. 7a).

Movie S6.

ER segmentation for WT1 iPSC-CM (corresponding to supplementary Fig. 7a).

Movie S7.

Reconstructed tomogram for MUT1 iPSC-CM (corresponding to supplementary Fig. 7b).

Movie S8.

ER segmentation for MUT1 iPSC-CM (corresponding to supplementary Fig. 7b).

Movie S9.

Reconstructed tomogram for WT1-cntrl iPSC-CM (corresponding to supplementary Fig. 9a).

Movie S10.

ER segmentation for WT1-cntrl iPSC-CM (corresponding to supplementary Fig. 9a).

Movie S11.

Reconstructed tomogram for WT1-chol iPSC-CM (corresponding to supplementary Fig. 9b).

Movie S12.

ER segmentation for WT-chol iPSC-CM (corresponding to supplementary Fig. 9b).

Movie S13.

Reconstructed tomogram for MUT1-DMSO iPSC-CM (corresponding to supplementary Fig. 12g).

Movie S14.

ER segmentation for MUT1-DMSO iPSC-CM (corresponding to supplementary Fig. 12g).

Movie S15.

Reconstructed tomogram for MUT1-pit iPSC-CM (corresponding to supplementary Fig. 12h).

Movie S16.

ER segmentation for MUT1-pit iPSC-CM (corresponding to supplementary Fig. 12h).

Data S1. (separate file)

Uncropped western blot membranes.
