# Supplementary material for: Targeting intracellular cholesterol imbalance rescues sarcomere–ER contact site signaling and ER remodeling in dilated cardiomyopathy
Source: Signal Transduct Target Ther. 2026 Jun 17;11:237. doi: 10.1038/s41392-026-02731-3 (PMC13273072; doi:10.1038/s41392-026-02731-3)

Full unedited membrane scans for Fig. 4c

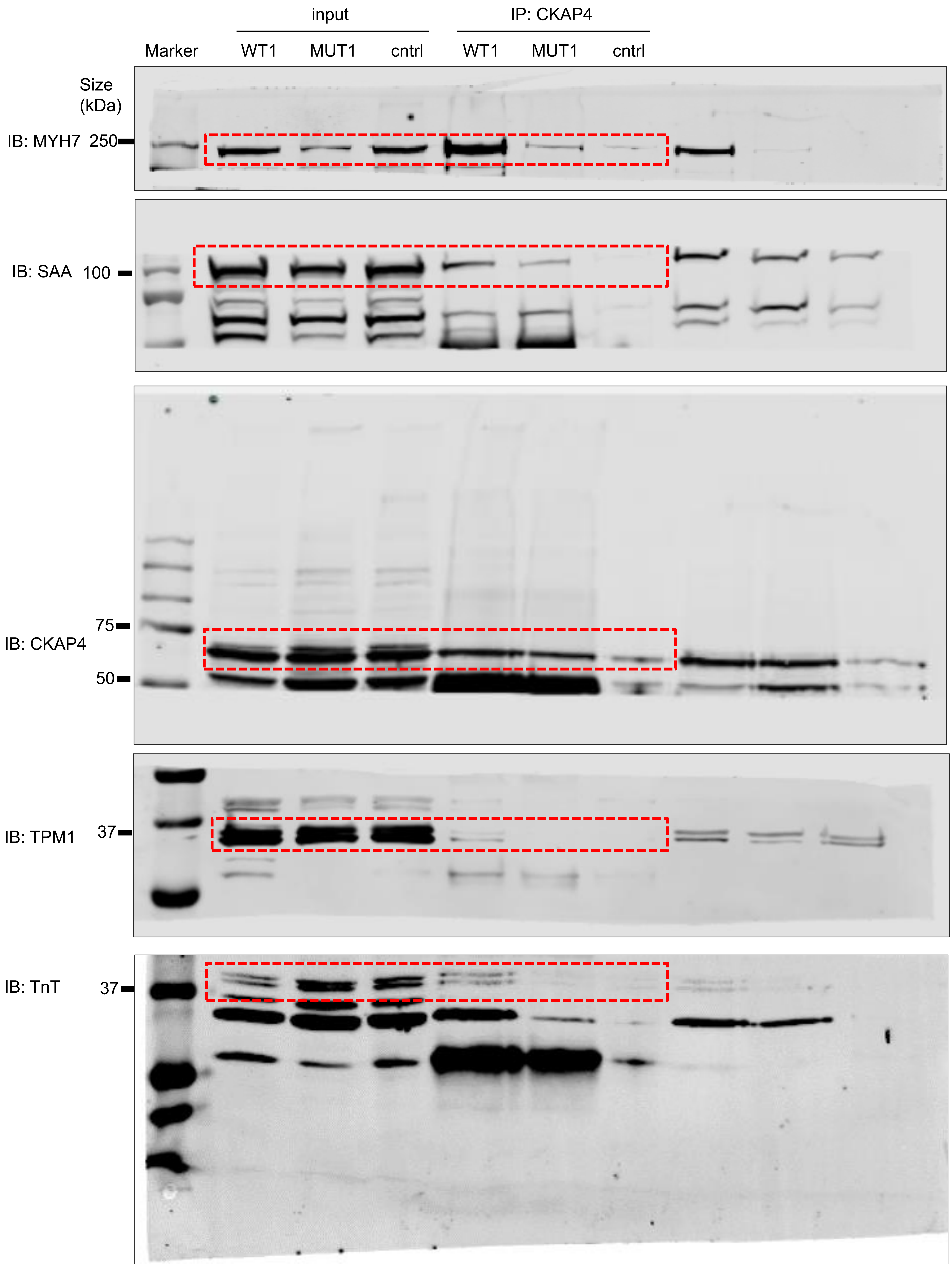

Full unedited membrane scans for supplementary Fig. 1c

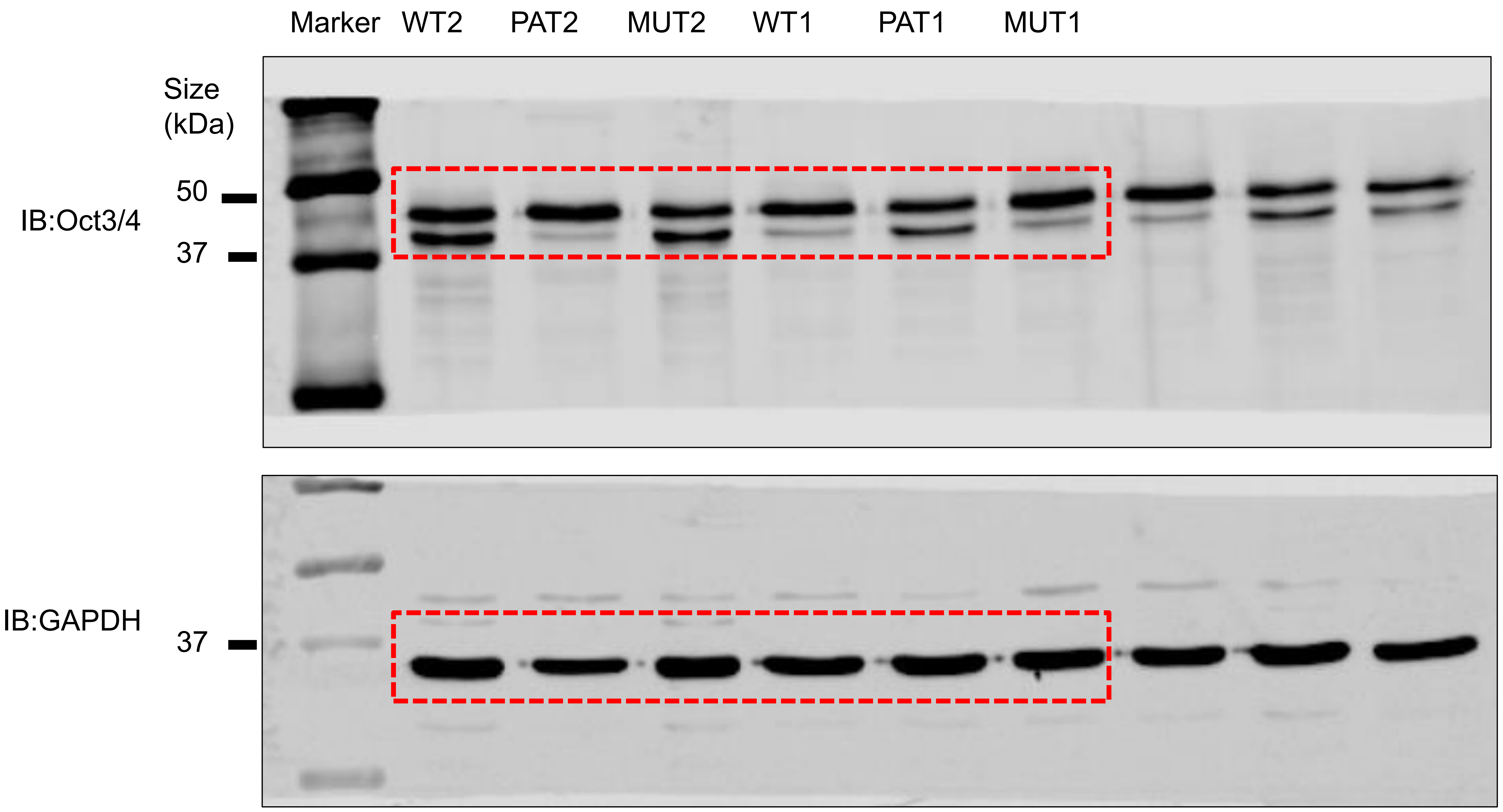

Full unedited membrane scans for supplementary Fig. 2e, 2h

e

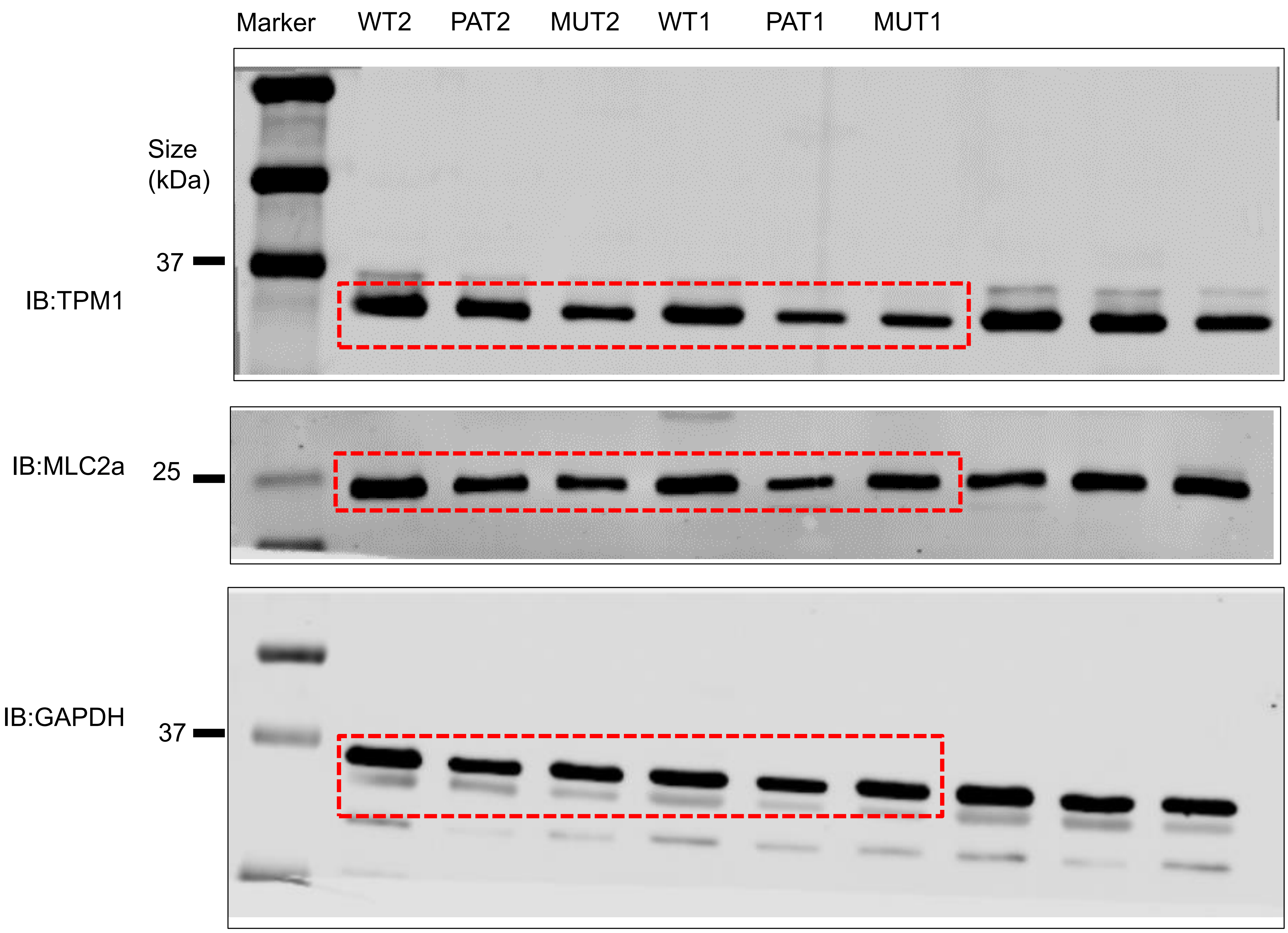

h

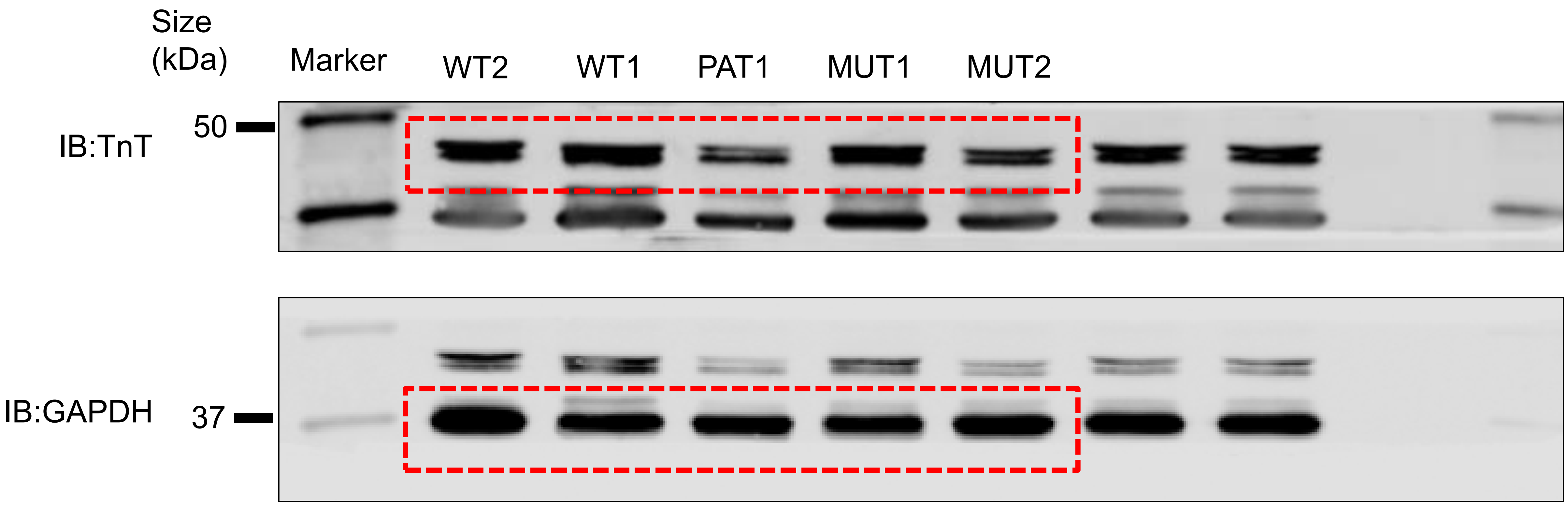

Full unedited membrane scans for supplementary Fig. 5b, 5f

b

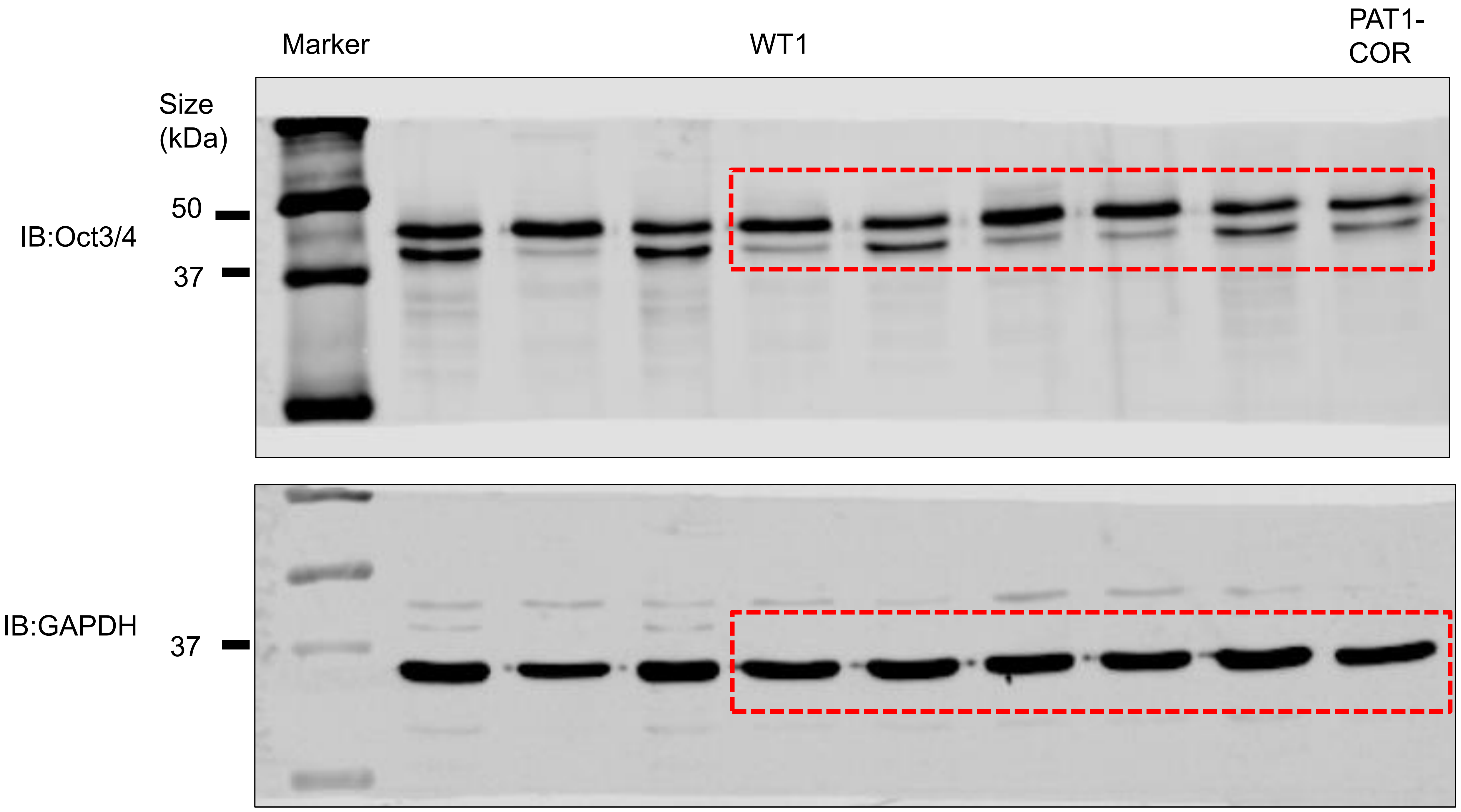

f

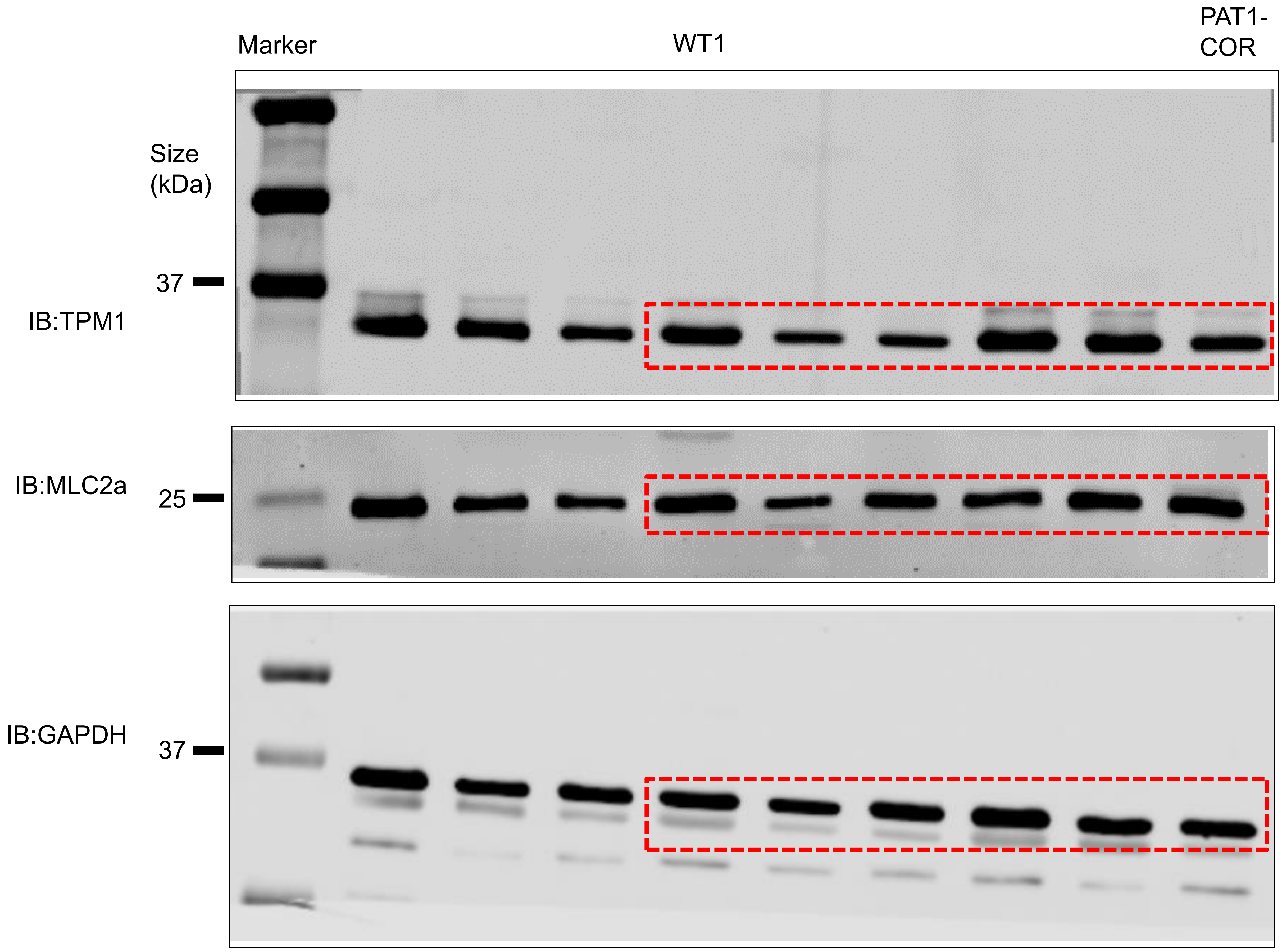

Supplement: Supplementary file 18 — Data S1 [file 41392_2026_2731_MOESM18_ESM.pdf]
